# Evidences for a role of two Y-specific genes in sex determination in *Populus deltoides*

**DOI:** 10.1038/s41467-020-19559-2

**Published:** 2020-11-18

**Authors:** Liangjiao Xue, Huaitong Wu, Yingnan Chen, Xiaoping Li, Jing Hou, Jing Lu, Suyun Wei, Xiaogang Dai, Matthew S. Olson, Jianquan Liu, Mingxiu Wang, Deborah Charlesworth, Tongming Yin

**Affiliations:** 1 The Key Laboratory of Tree Genetic Improvement and Biotechnology of Jiangsu Province and Education Department of China, Nanjing Forestry University, 200137 Nanjing, China; 2 Department of Biological Sciences, Texas Tech University, Lubbock, TX 79409 USA; 3 Key Laboratory of Bio-Resource and Eco-Environment of Ministry of Education, College of Life Sciences, Sichuan University, 610065 Chengdu, China; 4 Institute of Evolutionary Biology, University of Edinburgh, Charlotte Auerbach Road, Edinburgh, EH9 3FL UK

**Keywords:** Sexual selection, Evolutionary biology, Plant genetics, Plant molecular biology

## Abstract

Almost all plants in the genus *Populus* are dioecious (i.e. trees are either male or female), but it is unknown whether dioecy evolved in a common ancestor or independently in different subgenera. Here, we sequence the small peritelomeric X- and Y-linked regions of *P. deltoides* chromosome XIX. Two genes are present only in the Y-linked region. One is a duplication of a non-Y-linked, female-specifically expressed response regulator, which produces siRNAs that block this gene’s expression, repressing femaleness. The other is an LTR/Gypsy transposable element family member, which generates long non-coding RNAs. Overexpression of this gene in *A. thaliana* promotes androecium development. We also find both genes in the sex-determining region of *P. simonii*, a different poplar subgenus, which suggests that they are both stable components of poplar sex-determining systems. By contrast, only the duplicated response regulator gene is present in the sex-linked regions of *P. davidiana* and *P. tremula*. Therefore, findings in our study suggest dioecy may have evolved independently in different poplar subgenera.

## Abstract (web summary)

Dioecy has evolved independently from hermaphroditic ancestors in different plant lineages. Here, the authors assemble *Populus deltoides* male and female genomes, and show the putative roles of a femaleness gene and a maleness gene in sex determination, which suggests independent evolution in different poplar species.

## Introduction

Most flowering plants produce bisexual or ‘perfect’ flowers (hermaphroditism), but ~10% of angiosperm species bear unisexual flowers, either with male and female flowers on the same plant (monoecy) or on separate individuals (dioecy)^1^. Dioecy has evolved independently hundreds of times from hermaphroditic ancestors, in multiple plant lineages^1^, and recent advances have allowed details of the genetic mechanisms of sex determination to be understood in several dioecious plants^2–5^. A theoretical model for the origin of sex chromosomes involves a transition from functional hermaphroditism (including monoecy) to dioecy via mutations in two linked genes, affecting female and male functions^6,7^. Recent empirical studies in garden asparagus (*Asparagus officinalis* L.)^5^ and kiwifruit (*Actindia rufa*  × *A. chinensis*)^8,9^ have revealed fully Y-linked sex-determining genes that support this hypothesis^4^.

A two-gene system is not inevitable. First, both mutations could occur in the same gene. Second, a single-gene sex determination, involving a gene that dominantly suppresses female function and promotes male function can be experimentally created in monoecious plants, including maize^10^ and melon (*Cucumis melo*)^11^, by mutations in unlinked but interacting genes, one of which acts in a sex-specific manner. Sex determination in persimmon (*Diospyros lotus*, a tree) is a naturally evolved single-gene system, involving a non-coding RNA locus, *OGI*, suppressing femaleness^4^. However, two mutations were necessary for its evolution, and the target gene *MeGI* is also inferred to have changed during the evolution of females^12^.

Different genetic factors therefore clearly control sex determination in unrelated flowering plant lineages, consistent with independent origins of dioecy^5^. Unlike the ‘cryptic dioecy’ in asparagus and kiwifruit, whose flowers bear apparently normal organs of the opposite sex, sex organ abortion in poplars and willows (family *Salicacese*) occurs early, before the initiation of stamen or carpel primordia, and dioecy may have evolved from a monoecious ancestral state, consistent with these plants bearing ‘catkins’ of small unisexual florets. In the genus *Populus*, chromosome XIX carries the sex determining locus^13–18^. Genetic mapping studies revealed that the locations of the sex determining gene differ in genera *Populus* and its sister genus *Salix*^19–23^, suggesting independently evolved sex determination within the family Salicaceae. Multiple candidate sex determining genes have emerged from genomic association studies and/or transcriptome expression analyses^24–28^, including a recent study in a poplar species, *P. tremula* that revealed a sex determining role for one of these candidates, which was named *ARR17* (*Arabidopsis Response Regulator 17*)^29^. In the present study, in another poplar species *P. deltoides*, we characterize the regulation and functions of candidate sex-determining genes. Our results suggest how dioecy may have evolved in *P. deltoides*, and support the view that dioecy evolved independently in *P. deltoides* and *P. tremula*.

## Results

### Mapping the sex-determining locus and reconstructing X and Y haplotypes

**Fig. 1:**
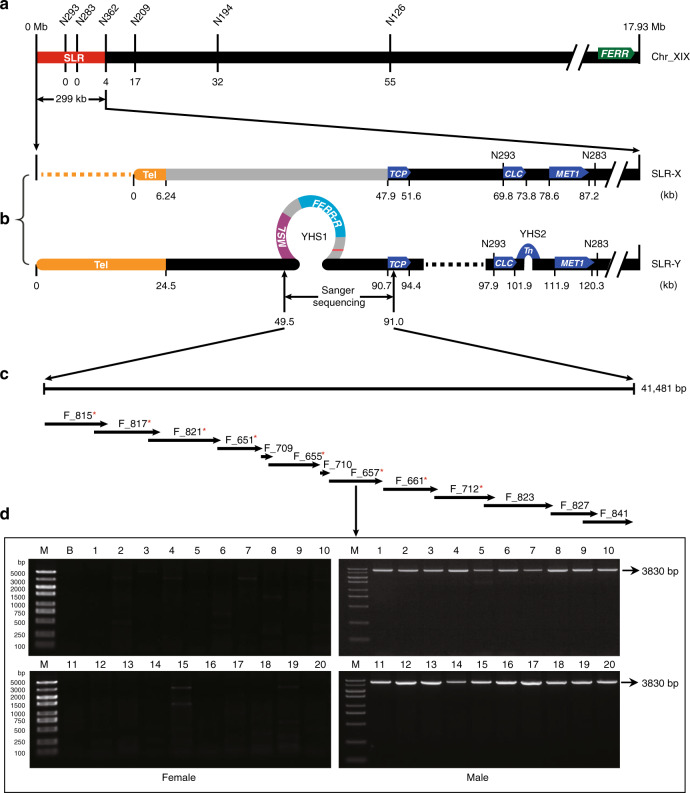
Reconstructed haplotypes in the SLR of P. *deltoides* chromosome XIX. **a** Genetic map and physical positions of the SLR (red bar) and the telomere. SSR makers are shown on the top and the numbers of observed recombination events are shown under the chromosome diagram, with zero in both the genetic and physical maps at the telomere end of the chromosome. The *FERR* gene is located at the right end of chromosome XIX, the end most distant from the telomere of this telocentric chromosome. **b** SLR-X and SLR-Y haplotypes reconstructed from genome sequences of our sequenced male (see main text). The yellow bars at the left indicate the X and Y telomeres, and physical distances (kb) from the telomere end are shown under each bar. The dashed lines represent deleted sequences, whereas the loops on SLR-Y represent the two Y-specific hemizygous sequence (YHS) described in the text. The gray portion on SLR-X indicates a region described in the text, where divergence between SLR-X and SLR-Y is higher than elsewhere in the SLR. **c** The Y-specific region rebuilt from 13 PCR amplified fragments (fragment names are shown above the arrows). Red asterisks indicate the fragments are further amplified with natural stands of *P. deltoides*. **d** Agarose gel electrophoresis profile for fragment F_657 in females and males. Similar results were obtained in two independent experiments. M molecular marker, B blank control. Source data underlying **d** are provided as a Source Data file.

Linkage analysis using simple sequence repeat (SSR) markers (Supplementary Table 1) located the *P. deltoides* sex-determination locus near the telomeric end of chromosome XIX (Supplementary Fig. 1), and confirmed male heterogamety (XY sex determining system). To identify the sex-determining gene(s), we sequenced and de novo assembled the genomes of a poplar female and one of its male offspring. The assembly for the female is 431 Mb, with contig N50 of 1.4 Mb, and 414 Mb with contig N50 of 2.8 Mb for the male. BUSCO analyses showed that 93.5% and 96.1% of plant conserved single-copy genes are complete in the female and male assemblies, respectively (Supplementary Table 2). Our SSR markers located the sex-determining locus to a 299-kb sex-linked region (SLR) between the telomeric region and marker N362 (Fig. 1a); we refer to the recombining region proximal to N362 as pseudo-autosomal (the PAR).

Because the sequenced male inherited his X chromosome from his sequenced female parent, we could use single-nucleotide polymorphisms (SNPs) to infer the complete SLR-X and SLR-Y haplotypes in the sex-linked region (Fig. 1b). The X and Y haplotypes’ telomeric repeat regions were assembled into two contigs, of sizes 104 kb and 141 kb, both well supported by mapped raw PacBio reads; the annotated repetitive sequences total 6.2 kb for the X telomeric repeat region, versus much longer (24.5 kb) for the Y haplotype (Fig. 1b). Annotation predicted 41 genes in the SLR-X haplotype and 26 in SLR-Y. At least 16 genes with assigned functions were found in both the SLR-X and -Y, including a cluster of 5 tandem genes encoding leucine-rich repeats (LRR) receptor-like protein kinases (Supplementary Table 3). Supplementary Figure 3 shows the alignment of homologous genes between SLR-X and -Y. Divergence between genic sequences on the SLR-X and -Y is highest for genes neighboring the telomeric repeat regions, and some are unalignable (Fig. 1b and Supplementary Fig. 2). Taken together, the differentiation of Y and X haplotypes across the 299 kb region is strong evidence for a completely sex-linked region at the telomeric end of chromosome XIX.

### Identifying sex determining genes

**Fig. 2:**
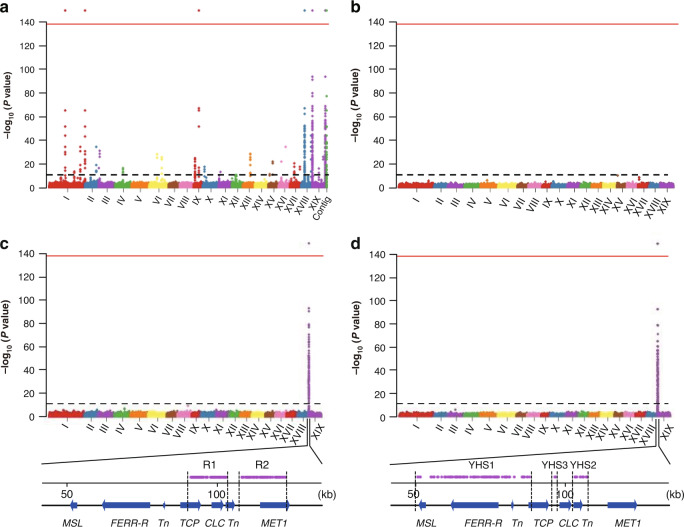
Manhattan plots of GWAS analysis of *P. deltoides* sex phenotypes. The *y*-axis shows the negative logarithm of *P* values from Wald Chi-Squared Test implemented in GEMMA. The black dashed line above the *x*-axis indicate the cut-off *P* value = 1e-9 (corresponding Bonferroni significance = 0.01). The red line at the top of each diagram indicate cut-off *P* value = 1e-137 (corresponding to Bonferroni significance = 1e-130). The Roman numerals under the *x*-axis indicate the chromosome identity. **a** shows results of SNPs GWAS using the female genome as the reference (‘Contig’ on the *x*-axis represents the unplaced Contig01665). **b** shows results of coverage GWAS using the female genome as the reference. **c** shows results of SNPs GWAS using SLR-Y as reference. **d** shows results of coverage GWAS using SLR-Y as reference. R1, R2, YHS1-YHS3 at the bottom of **c** and **d** indicate regions contain GWAS signals completely associated with sexes. Note that the *FERR-R* and *MSL* genes are not shown as significant in **c**, because the SNP analysis is not relevant to these genes, since they are absent from the X haplotype.

To identify the *P. deltoides* sex determining factors, we performed a genome-wide association study (GWAS) based on SNPs, using 49 female and 46 male trees (Supplementary Table 4). Genome resequencing generated a total of 1.15 Tb of Illumina reads with sequence depths of at least 20× for each of the 95 trees (Supplementary Table 4). With the sequenced female as the reference, we detected 435 SNPs with genotypes matching the individuals’ sexes, assuming male heterogamety (SNPs that are homozygous in all females in our samples, but heterozygous in all the males; Fig. 2a and Supplementary Table 5). We refer to such SNPs as SEMSs (SNPs exactly matching with sexes). 315 SEMSs are within three SLR genes, T-complex protein 1 subunit gamma (*TCP*), Chloride channel protein CLC-c (*CLC*), and DNA-methyltransferase 1 (*MET1*). In total, 120 are elsewhere, of which 78 are in a chromosome XIX PAR gene (Fig. 1a) that belongs to a gene family of response regulators (*RR*) in the cytokinin signaling pathway^30^, and specifically to the *A. thaliana* type-A *RR* genes. We named this gene *FERR* (female-specifically expressed *RESPONSE REGULATOR*), based on evidence for its function described below. Phenotypes previously reported for mutations in two members of this *A. thaliana* gene family, *arr16* and *arr17*, include effects on plant photomorphogenesis, cell division activity and root hydrotropism, but not changes in floral organs^31–33^. However, a recent study demonstrated involvement of such a gene in female functions and sex determination in *P. tremula*^29^, and named the *FERR* ortholog *ARR17*^29^ (although, as discussed below, it is not the ortholog of the *A. thaliana ARR17* gene).

The other 42 SEM variants are in genes on three other chromosomes and one unplaced contig, Contig01665 (Fig. 2a); 27 are in an autosomal gene, *HEMA1*, on chromosome IX, and 15 in non-coding sequences or genes with unknown functions (Supplementary Table 5). Previous studies in *P. balsamifera*^26^ and *P. trichocarpa*^28^, using the assembled genome sequence of a *P. trichocarpa* female as the reference^34^, also found SNPs associated with sex on multiple chromosomes.

However, using a female genome as the reference will produce false positive SEMSs, because reads from Y-linked regions that are missing from the female genome will erroneously map to homologous sequences elsewhere in the genome. Examination of our *P. deltoides* non-SLR SEMSs indeed revealed sequence similarity with the SLR-Y (Supplementary Table 6). Use of the SLR-Y as our reference for GWAS analysis eliminated all the non-SLR SEMSs (Fig. 2c), leaving only the three SLR genes, *TCP*, *CLC*, and *MET1* genes as sex-determining gene candidates.

### Y-hemizygous genes

As mentioned above, some segments of the X and Y haplotypes are unalignable (Supplementary Fig. 2); these indicate sex-specific regions that could include further candidate sex-determining genes. To examine this possibility, we performed coverage-based GWAS in our 95 *P. deltoides* samples (see Methods section). With the SLR-Y as the reference, we detected three Y-specific hemizygous sequences (YHSs), two large ones, YHS1 in Fig. 1 (34.8 kb), the 4.3 kb YHS2, and one of only 100 bp (YHS3, in an intergenic region, which is not considered further). These sequences are present in all male trees and absent in all females (Fig. 2d). No female-specific sequences were detected using the SLR-X as reference (Fig. 2b). The YHS1 sequence was validated by amplifying and Sanger sequencing 13 overlapping fragments in the sequenced male (Fig. 1c, d). This yielded a 41,481-bp sequence identical to the SLR-Y sequence, which is therefore complete, and includes no gaps. We tested 20 trees of each sex from the GWAS samples for the presence of YHS1 using PCR primers designed to amplify eight separate fragments (Fig. 1c); PCR amplification succeeded in all the males but no females yielded amplification products (Fig. 1d), confirming male specificity.

Three gene models were predicted in YHS1, and one in YHS2 (Fig. 1b and Supplementary Table 3). Sequence analysis indicated that three gene models are transposable elements, but one gene (EVM0039006 in YHS1) is the duplication of *FERR* described above. We named the *FERR* duplicate *FERR-R*, standing for its inferred *FERR* repressor function, as deduced from experiments described below. A duplication of *FERR* was also recently found in the *P. tremula* sex-determining region (which is located in a different chromosome XIX region from that of *P. deltoides*), and was named *ARR17* inverted repeat^29^. EVM0039005 (also within YHS1) was found to produce long non-coding transcripts (see details below), and was named *MSL*, for male-specific lncRNA. Before describing our evidence about these functions, we first describe data about expression during flower development that eliminates the protein-coding genes in the fully sex-linked region as candidate sex determining genes.

### Expression patterns of fully sex-linked genes

**Fig. 3:**
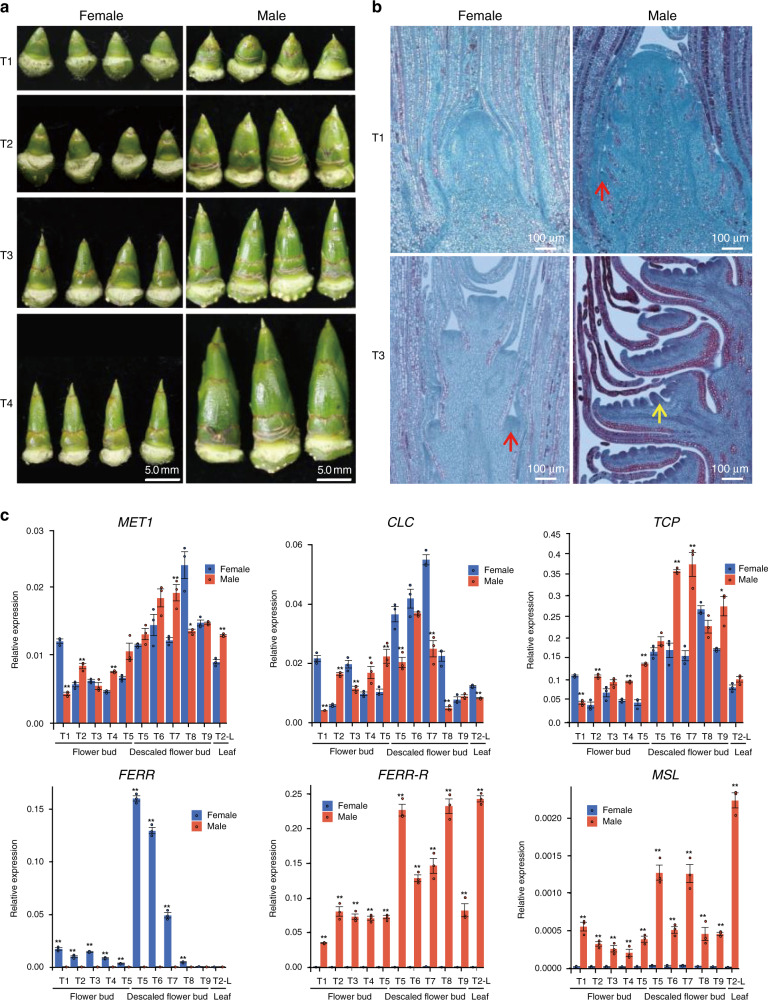
Development of flower buds and gene expression profiles. **a** Morphology of the male and female flower buds at developmental stages T1–T4 defined in the text. **b** Longitudinal sections of male and female inflorescences at T1 and T3. The red arrows point to the floret primordia, and the yellow arrow denotes the anther primordium. Three independent samples were observed with similar results. **c** Gene expression profiles in the 8 flower development stages described in the text. Values shown are the mean ± SD from three biological replicates (*P < 0.05; **P < 0.01, two-sided Student’s *t* test). The data for **c** are provided as a Source Data file.

Poplars have small florets, without petals or sepals, and many florets are attached to the rachis of morphologically different male or female catkins (Supplementary Fig. 4). A single male floret consists of a group of stamens inserted on a disk, while a female floret has a single-celled ovary seated in a cup-shaped disk (Supplementary Fig. 4). Poplars bloom in early spring before the flush of leaves, but female and male flower primordia start differentiating in June of the previous year (Fig. 3a). Four stages of sex organ development related to occurrence of doiecy are recognized^35^, T1 (before the initiation of stamen or carpel primordia), T2 (early stamen or carpel development), pre-meiosis (stage T3), and post-meiosis (stage T4); the Methods section defines the stages. Longitudinal sections of flower buds (Fig. 3b and Supplementary Fig. 5) showed that *P. deltoides* male and female flower primordia are distinguishable starting from early June (T1) and early July (T3), respectively. The sex-determining genes must therefore act at these early stages.

Our RNA-seq experiments detected no expression of two of the Y-specific transposable elements, eliminating them from consideration as candidate sex determining genes. In contrast, *TCP*, *CLC*, *MET1, FERR-R*, and *MSL* were expressed in all four early flower development stages. qRT-PCR bioassays showed that none of these genes has expression limited to flower tissue, and none shows a consistent sex difference in expression (Fig. 3c). The three protein-coding genes, *TCP*, *CLC*, and *MET1*, are present in both the SLR-X and -Y, making them unlikely candidates for the sex determining genes, given their lack of consistent sex differences in expression. However, the two SLR-Y hemizygous genes, *FERR-R* and *MSL*, are present only in male *P. deltoides*. Despite being expressed in all four early flower development stages, and not exclusively in flower tissue, these two non-protein-coding genes are considered as candidate sex determining genes in *P. deltoides*. We next describe expression and function data that support this view.

### *FERR-R* is a femaleness suppressor that generates siRNAs suppressing *FERR* function

**Fig. 4:**
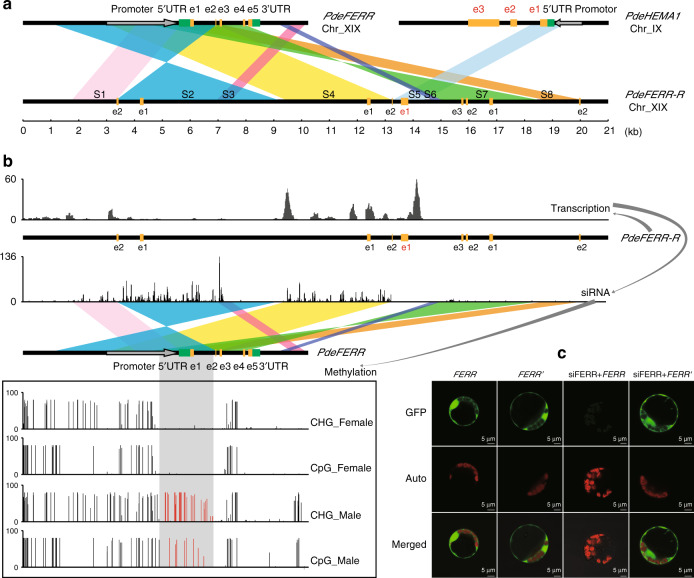
Origin of *FERR-R* and its function in repressing *FERR*. **a** Sequence homology analysis for *FERR*-*R*. e1–e5 represent the five exons of *FERR* (in black) or *HEMA1* (in red). S1–S8 indicate the duplicated segments described in the text. **b** Abundance of *FERR*-R transcripts and the *FERR*-*R* generated siRNAs, and the differential methylation of *FERR* in the two sexes. The gray shadow shows the region methylated only in males, and the red vertical bars indicate the methylation levels in this region. **c** Transient expression experiment in poplar protoplasts. siFERR is siRNA generated by *FERR*-*R*. *FERR*′ is the siRNA-resistant version of *FERR*. Three independent experiments were performed with similar observation.

The SLR-Y hemizygous gene *FERR-R* shows homology with *FERR* (a PAR gene) and with the autosomal *HEMA1* gene (Fig. 4a shows the alignments). Seven segments (S1, S2, S3, S4, S6, S7, and S8) show homology with *FERR*; these include all regions of *FERR* (the promoter region, 5′-UTR, exon 1, exon 2, exon 3, the first three introns, and two downstream segments) in a rearranged order compared with the progenitor copy, and with parts, including some of the promoter, and exon 1, appearing more than once in the chimeric *FERR-R* sequence (Fig. 4a). The *FERR-R* segment S5, between *FERR*-derived segments S4 and S5, shows homology to the *HEMA1* 5′-UTR and exon 1 (Fig. 4a).

Expression data from strand-specific lncRNA-Seq and small RNA-Seq revealed that the male-specific *FERR-R* copy is transcribed into long transcripts that generate small interfering RNAs (siRNAs; Fig. 4b). In other organisms, siRNAs have been found to guide the methylation of homologous DNA through RNA-directed DNA methylation^36^. We found that, in *P. deltoides*, siRNAs generated by *FERR-R* could guide methylation at the promoter, 5′-UTR, exon 1, and the first intron of the *FERR* gene (Fig. 4b). Bisulfite sequencing showed that methylation of the corresponding regions in *FERR* occurred specifically in males (Fig. 4b). In *P. balsamifera*, male-specific DNA methylation was also detected in the promoter and first intron of the homologous gene, *PbRR9*^26^. Besides inducing siRNA-directed DNA methylation, siRNAs produced by *FERR-R* were also found to target *FERR* exons 1 to 3, suggesting that *FERR-R* might also trigger siRNA-guided cleavage of *FERR* transcripts. Transient expression experiment in poplar mesophyll protoplasts confirmed that cleavage indeed occurred, and involved interaction between *FERR-R* and *FERR*. Green fluorescence signals were observed in poplar protoplasts transformed with *FERR*, *FERR*′ (a siRNA-resistant version of *FERR*), and after co-transformation with siFERR+*FERR*′, but not after co-transformation with siFERR+*FERR* (Fig. 4c). Supplementary Figure 6 shows a model of the interaction between *FERR*-*R* and *FERR*, based on the observations just described.

qRT-PCR revealed that *FERR* is expressed only during the initiation of female flower primordia and the early development of female flowers in *P. deltoides*. In the early stages of flower development, the scales form a large proportion of bud tissue, and the flowers are too small to separate into different tissues before stage T5. *FERR* is expressed in whole flower buds (including scales) of stages T1 to early stage T5. In stage T5 female flower buds, removal of the scales increased *FERR* expression more than 50-fold, indicating that high *FERR* levels in earlier stages were obscured by the presence of scale tissue. After stage T5, *FERR* expression decreased to a low level by stage T8 (Fig. 3c). The effects of the *FERR-R* duplication described above act by affecting expression of *FERR*, and must therefore be specific to early carpel development, even though, as described above, expression of *FERR-R* is not specific to early bud stages.

**Fig. 5:**
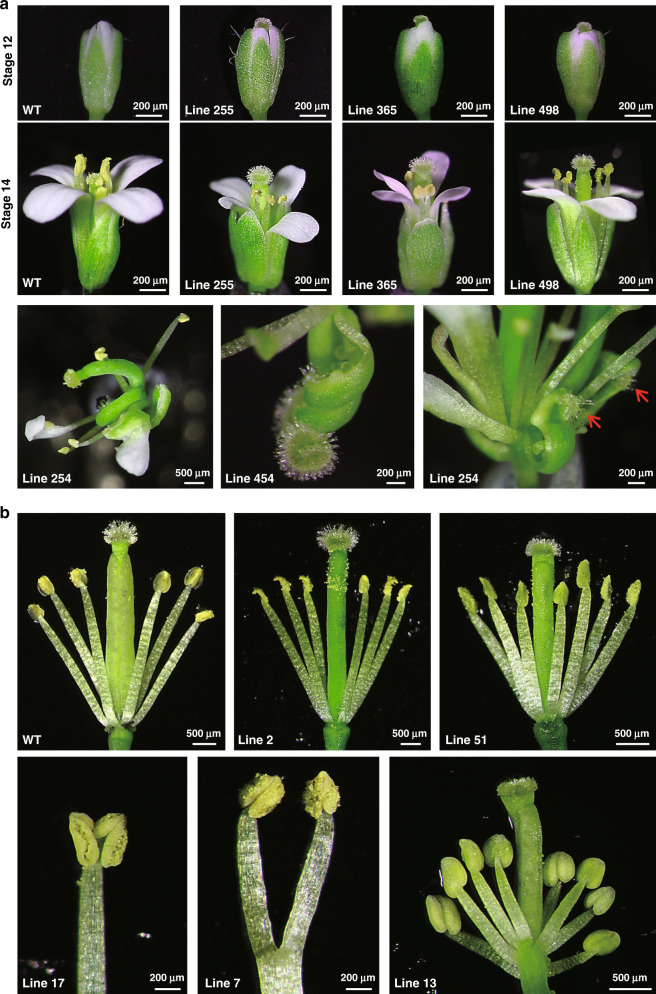
Floral characterization of wild type and transgenic *Arabidopsis* plants. Each line number represents an independent transgenic line. **a** The first and second row show the stigma hypertrophy and exsertion commonly observed in *FERR*-overexpressing plants; the third row shows extreme floral phenotypes observed in two *FERR*-overexpressing lines. A flower with two bent pistils (left) and one with carpel-like sepals (right, red arrow) were found in line 254, and line 454 (middle) produced a flower with one bent pistil and an unfused carpel. Due to the abnormal floral structures, it is difficult to stage the flowers in the third row from the top. However, by relying on the developmental stage of petals, stamens, and sepals, we classified these flowers as late stages 15–16. **b** Dissected flowers of wild type (WT) and *MSL*-overexpressing plants. WT flowers have four long and two short stamens (tetradynamous stamen), whereas the flowers from *MSL*-overexpressing plants had six long stamens (line 2), extra stamens (lines 51 and 13), or reduplicated Y-shaped stamens (lines 7 and 17).

*FERR*-like genes resembling *A. thaliana ARR17* were among candidate sex determining genes in several previous studies of poplar and willow species (*PbRR9* in *P. balsamifera*^26,37^, *PtRR9* or *PtRR11* in *P. trichocarpa*^38^, *ARR17* in *P. tremula*^29^, *RR* in *Salix purpurea*^22^). Phylogenetic analysis of type-A *RR* genes (Supplementary Fig. 7) shows, however, that neither the *P. deltoides FERR* (EVM0009215.1) nor *P. trichocarpa FERR* (Potri.019G133600.3) is orthologous to the *A. thaliana ARR17* gene (the closest sequence is another gene in this family, EVM0036439.1). To investigate FERR’s function, we therefore made transgenic *A. thaliana* overexpressing *P. deltoides FERR*. These had normal androecium development, but often showed stigma exaggeration, in extreme cases producing flowers with two pistils or carpel-like sepals (Fig. 5a). We, therefore, propose that *FERR* is a female-specifically expressed *P. deltoides* response regulator (*RR*) that promotes female functions during the initiation of female flower primordia and early carpel development. Such a function is consistent with our evidence above that the Y-linked *FERR-R* gene suppresses *FERR* functions in *P. deltoides* males, and corresponds to the hypothesized female suppressor, or *Su*^F^, involved in the evolution of dioecy^7^. In our model, the pre-duplication *FERR* gene of the ancestor promoted female functions, and suppressing these created males in the dioecious descendant species. Type-A *RR* genes have a very complex regulatory network, not currently well characterized, and, although transcriptomic data from our transgenic *A. thaliana* overexpressing *FERR* does detect genes with altered expression (Supplementary Data 1 and Supplementary Table 7), they do not illuminate *FERR*’s function.

### *MSL* is a candidate gene that generates long non-coding RNAs promoting maleness

Raw reads matching the *MSL* sequence in our male genome sequence were extracted and assembled to confirm the male-specificity in our GWAS samples (Supplementary Fig. 8a, b), and PCR amplification succeeded in all the males but no females yielded amplification products, confirming male-specificity (Supplementary Fig. 8c). Based on a de novo repeat library constructed from *P. deltoides* genome sequences (see Methods section), *MSL* is annotated as a transposable element in the LTR/Gypsy transposon family. Consistent with being a transposable element, we found large numbers of partial sequences with homology to the 5′ end of the *MSL* sequence (Supplementary Fig. 9), on all *P. deltoides* chromosomes (Supplementary Data 2, *MSL* homology regions in *Populus* and *Salix* species). However, the only complete copy was the one in YHS1. *MSL* sequences are completely absent from the *A. thaliana* and *Oryza sativa* genomes. Although most other poplar and willow species have only partial sequences (Supplementary Data 2), a complete *MSL* is present in the genome of a male *P. simonii* (in a different subgenus from *P. deltoides*, see Supplementary Data 2). This sequence is also in a YHS at the peritelomeric end of chromosome XIX, as in *P. deltoides*, and is absent from females of this species, and could be involved in sex determination in these two species.

A function for a transposable element is not implausible, as such elements generate lncRNAs in many species^39–43^. qRT-PCR revealed continuous expression of *MSL* in male *P. deltoides* (Fig. 3c). Transcripts are detected in lncRNA-seq, but not general RNA-Seq (Supplementary Fig. 10a), indicating that *MSL* indeed produces lncRNAs transcripts lacking a 3′ poly-A tail. To test for a phenotypic effect of *MSL*, we over-expressed it in *A. thaliana*. Overexpression of *PdeMSL* in *A. thaliana* (to levels 5- to 20-fold higher than levels in *P. deltoides*) did not affect the pistils, and seed set was unaffected, but did affect the androecium, commonly resulting in flowers with six long stamens, or seven or occasionally 8 stamens, stamens bearing two anthers, or branched stamens (Fig. 5b), versus the four long and two short stamens of wild-type *A. thaliana* flowers. These androecium-specific effects suggest that the *MSL* gene may promote maleness in *P. deltoides*. Supporting this, GO analysis revealed that genes with significantly increased expression in our transgenic lines (Supplementary Data 3, gene expression data from *A. thaliana* overexpressing *MSL*, Supplementary Table 7), show significant enrichment for pollen development functions (Supplementary Fig. 11).

### Sex determination also involves *FERR* duplication in *P. davidiana*

*P. deltoides* belongs to subgenus *Aigeiros* in the genus *Populus*. To test whether the hemizygous YHS1 region is present in other poplars, we sequenced the genome of a male *P. davidiana*, in the same subgenus, *Leuce*, as *P. tremula* and *P. tremuloides* (in an earlier-branching section of *Populus* than *Aigeiros*^44^); sex-determining regions of all three subgenus *Leuce* species map to the pericentromeric region of chromosome XIX^17,29^. Coverage analysis of genome sequences of 49 *P. davidiana* females and 47 males, using the male sequence as the reference, detected a 126 kb YHS on a contig (ctg345, Supplementary Fig. 12) that again includes a *FERR-R*-like gene (named *PdaFERR-R*). *PdaFERR-R* shares twelve duplicated segments with its putative progenitor, *PdaFERR*, but, unlike the *P. deltoides* duplication, only exon 1 of *FERR* is duplicated, and no *HEMA1* exons are present (Supplementary Fig. 12). Aligning the sequences flanking *P. davidiana’*s YHS to *P. deltoides* and *P. trichocarpa* genomes revealed that this YHS is in the pericentromeric region of chromosome XIX. The different locations in species in the two subgenera support the hypothesis that their dioecy evolved independently^29^, yet involved a similar mechanism.

Neither *P. davidiana*, nor *P. tremula* has a complete *MSL* sequence, and therefore probably have no active *PdeMSL* orthologs. Partial *MSL* sequences were found in contigs in many genome regions, but no lncRNA transcripts were detected from the *P. davidiana* chromosome XIX peritelomeric and pericentromeric regions copies (Supplementary Fig. 10b).

## Discussion

The development of female and male flowers in plants usually involves genes with spatially highly specific expression within the plant body or the flower primordia, and specific developmental timing. Most sex-limited and sex-biased genes in plants are not carried on the sex chromosomes, and their expression levels are probably controlled by an upstream sex-determining gene or genes^31,45^. Many genes probably function in the development of sexual dimorphisms of poplars, but we propose that *FERR-R* is the upstream female-suppressor gene. In *P. deltoides* XX females, *FERR* function is active due to the absence of the *FERR-R* gene, which is male-specific (being present only as a Y-linked copy in the YHS1 of the Y haplotype, and absent from the X-linked region). Although *FERR-R* expression is not confined to the flower development stage when sex determination occurs, temporal specificity is provided by *FERR*, which is expressed only during the initiation of carpel primordia and early female flower development, and our transformation experiments in *A. thaliana* demonstrate that it is a female promotor. *FERR* belongs to the type-A *RR* gene family of transcription repressors in cytokinin signaling^30^, and altering cytokinin signaling has been reported to affect flower development^46^. *RR* genes have been suggested as sex determiners in other plants, including kiwifruit^8^, *S. purpurea*^22^ and *Ginkgo*^47^. Finally, a recent study showed that, in *P. tremula* (in subgenus *Leuce*, like *P. davidiana*), knockout of the ortholog of the *P. deltoides FERR* gene (*ARR17* in *P. tremula*), in female trees converted them into males, indicating that *P. tremula* has a single-gene sex determination system^29^.

However, the hypothesis that *FERR-R* suppresses femaleness, creating males, does not account for the evolution of females from the cosexual ancestor from which dioecious poplars evolved. This ancestor must have had a functional female-promoting *FERR* gene, or a similar *ARR17* homolog. Its loss of activity through partial duplication could have created male, which would produce a population with cosexuals and males (termed androdioecy). Androdioecy is, however, extremely rare, and requires very unusual conditions to evolve^7^. Even if such an evolutionary change did occur, another change is still necessary to generate females. This would require a mutation causing loss of male functions, to convert the cosexuals into females. A mutation within the same gene as the femaleness suppressor is formally possible, resulting in a single-gene sex determination system. However, it seems unlikely that the *FERR* gene could also produce male-sterility mutations, given *FERR-R*’s mode of action to create males, involving siRNA-guided cleavage of *FERR* transcripts, and our evidence that (in *A. thaliana*) over-expression affects only the pistils. A second gene therefore seems to be required to explain the evolution of dioecy in poplars.

Two possibilities exist for this second gene. First, a mechanism similar to the one discovered in persimmon is possible: a first mutation in an existing gene creates females, and then the gene involved becomes duplicated, suppressing the first mutation’s male-suppressing effects, and thus creating males. In this scenario, the second mutation is a dominant femaleness suppressor, and hence the first mutation becomes redundant for sex determination, and can become fixed in the species, leaving presence/absence of the duplication in control of sex organ development. A second possibility is that the *MSL* sequence is a domesticated transposable element that produces lncRNAs that promote male functions. If the cosexual ancestor possessed this gene, its loss would create females, but the gene would remain present in the male-determining region (as observed in the fully Y-linked region of *P. deltoides*). Our findingthat *MSL* over-expression affects the *A. thaliana* androecium, but not the gynoecium, and alters expression of genes with male functions, is consistent with this possibility.

To test this possibility further, we asked whether *MSL* sequences are shared between different poplar species. Presence of a TE sequence at the same location in multiple species is unlikely, because TE insertions are rarely fixed in all individuals of a species. Our finding of a complete *MSL* sequence in *P. simonii* (in a different subgenus, *Tacamahaca*, from the one, *Aigeiro*s, that includes *P. deltoides*, see Supplementary Data 2), therefore suggests that that this sequence may have a function in these species. The retention of a complete sequence of such an insertion for a long evolutionary time, corresponding to the divergence of two subgenera, is unexpected, and could signify that it has a plant function, such as increasing male functions. Alternatively a mutation that increased male functions could have arisen on chromosome XIX after the chromosome acquired its male-determining locus. If it arose on a Y haplotype that happened to carry the *MSL* insertion, the resulting selective sweep would have led to this haplotype replacing the ancestral version (though the maintenance of its complete sequence remains surprising). However, function of *MSL* is evident in heterologously expressed *A. thaliana* in this study. Without a test of its function in poplar, such as a knockout, we cannot currently determine its role in androecia development in poplar. Moreover, findings in this study show that *MSL* cannot be essential for male functions in all Salicaceae species, given that other poplar and willow species have only partial sequences, and that the knockout of a single gene in *P. tremula*, *ARR17*, converted female trees into males^29^.

In *P. davidiana*, in the same subgenus as *P. tremula* (subgenus *Leuce*), only partial *MSL* homologous sequences are present, and the *FERR-R* duplication is in the pericentromeric region of chromosome XIX (Supplementary Data 2). These two differences from the findings in subgenera *Aigeiro*s (*P. deltoides*) and *Tacamahaca* (*P. simonii*) suggest that dioecy evolved independently in these taxa, and in subgenus *Leuce*. It is unknown how females of *P. davidiana*, or *P. tremula* evolved. If a gene like *MSL* was involved, it might no longer be essential after *FERR-R* appeared, and have been lost. It will be important in the future to locate the sex determining loci in more poplar species, to determine whether a peritelomeric location generally correlates with the presence of a complete *MSL* gene in the region, and a pericentromeric location with its absence. Nevertheless, the single-gene and possible two-gene systems in different sections of *Populus* make this genus particularly interesting for studying the evolution of sex determination in plants.

Many sex chromosomes, including the mammalian, bird, and Drosophila Ys, have evolved suppressed crossing over, sometimes followed by loss of large numbers of genes present on the X (and on the Y ancestor), causing hemizygosity in males, a process known as genetic degeneration^48,49^. The *P. deltoides* sex-determining genes also appear to be in a non-recombining region, but it is physically much smaller than in these animals, and even than the regions in plants such as papaya^50,51^, kiwifruit^52^, and asparagus^5^. Our analysis revealed that *FERR-R* originated by segmental duplication of the *FERR* gene (perhaps following a male-sterility mutation involving deletion of the *MSL* gene from the genome region into which the duplicate copy inserted, or perhaps followed by insertion of an *MSL* sequence into the region). The resulting hemizygosity of the *P. deltoides* Y-specific region is thus not due to degeneration, but to these two separate mutations. The hemizygosity can explain why the region carrying the sex-determining gene does not recombine between the SLR-Y haplotype and the X counterpart. Intriguingly, a hemizygous region was also found in asparagus^5^, and also in the date palm, *Phoenix dactylifera* (whose femaleness suppressor in males may also act via expressing a truncated competitor)^53^, suggesting that this deletion/insertion model represents a previously unrecognized route for the evolution of plant fully Y- and X-linked regions, similar to the evolution of hemizgous non-recombining regions controlling flower morphs in distylous plants^54,55^. Epigenetic regulation of reproductive genes is common in sex determination mechanisms in plants and animals^56^, including in the sex-determining mechanism by which monoecious plants develop male or female flowers in differing developmental contexts, rather than under control of different plant genotypes^57^. Specifically, miRNAs and lncRNAs have been found to be involved^58,59^, including an RNAi process in the plant *Diospyros kaki* (persimmon), in which a duplicated gene called *OGI* encodes a small RNA targeting the autosomal *MeGI* gene, regulating anther fertility, and determining individuals’ sex^4^. The sex-determining function of the *FERR* duplications now discovered in poplars of different sections involves both RNAi and DNA methylation processes, and, in *P. deltoides* and *P. simonii*, it is possible that the *MSL* transposable element might have become domesticated and evolved a function promoting androecium development.

## Methods

### Plant materials

To map the sex locus, an intraspecific F_2_ population of *P. deltoides* was established in 2012 by crossing two randomly selected siblings from an F_1_ full-sib family. A total of 1077 offspring (550 females and 527 males) of the F_2_ population were planted (6 × 6 m spacing) and maintained at Sihong Forest Farm in Jiangsu, China. To obtain the reference genomes for female and male *P. deltoides*, we sequenced the maternal parent and a randomly selected male progeny from the F_2_ mapping pedigree. For genome-wide association study (GWAS), we sequenced the genomes of 49 unrelated females and 46 unrelated males (referenced as GWAS samples of *P. deltoides*), which were selected from the *P. deltoides* germplasm collections maintained at Sihong Forest Farm. The *P. deltoides* germplasms plantation (6 × 6 m spacing) was established with 12 ramets for each clone following a completely random block design in 1998.

To characterize the developmental processes of flower buds, we collected the female and male flower buds from a female and a male tree in *P. deltoides* germplasms at nine different times: T1 (June 3), T2 (June 18), T3 (July 3), T4 (July 18), T5 (August 3), T6 (August 18), T7 (September 3), T8 (December 1), and T9 (January 15). To quantify the expression levels of genes, flower buds at the corresponding times were separately collected from three ramets of the female and male *P. deltoides* for biological replications. In the early developmental stages, it is difficult to harvest enough tissue for molecular experiments, and thus the flower buds with scales were used for T1–T5. For T5–T9, the descaled flower buds were used in molecular experiments. Since flower tissue only accounts for a small portion of the flower bud, we measured expression by using both the scaled and descaled flowers at T5 for comparison purposes.

Apart from *P. deltoides*, we also explored sex determination in *P. davidiana*, a more primitive species belonging to the subgenus *Leuce*^44^. Leaf samples of *P. davidiana* were collected from a natural population along Datong River in Qinghai, China in spring 2019. GWAS samples of *P. davidiana* included 49 females and 47 males.

### Sequencing, assembly, and annotation of *P. deltoides* genomes

Young leaves of the maternal parent and a randomly selected male progeny were collected for DNA extraction and sequencing libraries construction. The detailed description of sequencing, assembly, and annotation processes are provided in Supplementary Methods 1 and 2.

### Mapping the sex locus of *P. deltoides*

To map the sex locus, complete genetic maps were built for the female and male parents with 94 randomly selected offspring by using AFLP markers following the two-way pseudo-testcross strategy. Generation of AFLP markers was performed following the description in Yin et al.^60^. With the established genetic maps, whole-genome scan for sex locus was performed to locate its general position. Referring to the poplar consensus map^3^, we developed simple sequence repeat (SSR) markers in the vicinity region of the sex locus. The designed SSRs were then genotyped and mapped on the AFLP maps to saturate the target chromosomal region. In the mapped SSRs, we further selected six closely sex-linked SSRs to conduct fine local mapping. The selected SSRs were genotyped with 1077 flowered progenies to confine the sex locus in a more precise interval. To ensure the accuracy of sex phenotyping, the sex of each tree was separately recorded by three teams in two rounds of observation. For the few trees where results from three teams differed, further branches were collected to definitively determine the sex.

### Validation of the SLR haplotypes

To validate the reconstructed SLR haplotypes for the Y-specific region as described in Supplementary Method 3, sequence-specific primers were designed according to SLR-Y, and these primers were used to amplify DNAs extracted from the sequenced male. All PCR reactions were performed with PrimeSTAR® GXL Premix (Takara, China) according to the user manual. Details of the primers and PCR conditions were listed in Supplementary Table 1. The amplification products of each reaction were purified using the AxyPrep DNA Gel Extraction Kit (Axygen Scientific, USA), cloned into pEASY-Blunt vector (Transgen Biotech, China), and then sequenced on the Sanger sequencing platform ABI 3730xl (Applied Biosystems, USA). The obtained sequences were assembled into an integrated contig and aligned to SLR-Y to evaluate the reliability of the reconstruction for the Y-specific region. The conservation of the Y-specific region was further confirmed by PCR amplification with Y-specific primer pairs of 651F/R, 655F/R, 657F/R, 661F/R, and 712F/R (Supplementary Table 1) against DNAs extracted from 20 male and 20 female *P. deltoides*, which were randomly selected from the GWAS samples. The amplification products were examined by electrophoresis on 1% agarose. To identify duplication segments of *FERR* gene in SLR haplotypes, genomic sequence of *FERR* was applied to query the haplotype sequences using blast software.

### GWAS analysis of sex determination

Genomic resequencing was performed for the GWAS samples using Illumina platform. After removing low-quality reads and trimming of adapter sequences, the clean reads were mapped onto the reference genomes. Two versions of references were applied for GWAS. The consensus sequence of the female genome was selected in the first version, whereas the second version SLR-X was substituted by SLR-Y. Freebayes v0.9.10^61^ was applied to call variants including SNPs, short Indels, and multiple nucleotide polymorphisms. The setting ‘–use-best-n-alleles 6’ was applied for Freebayes to limit the total numbers of alleles at each site. For the sake of brevity, all these variants were referred to as SNPs in our analyses. The variants (SNPs) were screened at population level using plink v1.9^62^ with settings ‘–maf 0.05–geno 0.1’ and converted into gene dose file using qctool v2.0.1. GEMMA v0.98.1 software^63^ was used to perform SNP-based GWAS (snp-GWAS) between SNPs and sex phenotypes using linear mixed model (LMM) model.

In read-coverage based GWAS (rb-GWAS), 100-bp windows were generated across the whole genome to calculate the read depths. To test whether the presence or absence of the genomic fragments is associated with individuals’ sexes, the windows were grouped into three read-coverage categories: 0 for no read, 1 for coverage 1–2, and 2 for coverage ≥3 (indicating a reliable number of reads, a widely used threshold to screen reliable mapping to a reference genome sequence). Only primary (best) locations were selected for read counting. The windows associated with sex phenotypes were identified using GEMMA following the description as in snp-GWAS.

### Characterizing flower development and quantifying genes expression

Flower buds at different times (T1–T4) were collected and fixed using formalin-acetic acid. The samples were further dehydrated in a graded concentration of ethanol and embedded in paraplast. Serial sections were prepared by employing a Leica microtome. The sections were then mounted on microscope slides for staining with 1% safranin and examined using Carl Zeiss Imager M2 microscope (Zeiss, Germany).

RNA prep Pure Plant Kit (Tiangen, China) was used to extract RNAs from flower buds and leaf tissues. TransScript One-Step gDNA Removal and cDNA Synthesis SuperMix (TransGen Biotec, China) were used to reversely transcribe RNA to cDNA. cDNAs transcribed by Oligo dT primer were used to analyze the relative expression of coding genes, and those transcribed with random primers were used to analyze the relative expression of lncRNAs. AceQ qPCR SYBR Green Master Mix (Vazyme, China) was used to perform Quantitative real-time PCR (qRT-PCR) on a 7500 Fast Real-Time PCR System (Applied Biosystems, USA). In each 20 μL reaction volume, 100 ng of cDNA was used as templates. The PCR parameters used were as follows: 95 °C for 3 min, 40 cycles of 95 °C for 15 s, 60 °C for 15 s, and 72 °C for 30 s. Gene-specific primers were designed for *MET1*, *CLC*, *TCP*, *FERR*, *FERR-R*, and *MSL* (Supplementary Table 1). *Populus UBIQUITIN* (*PtUBQ*) gene was selected as an internal reference^64^. The relative expression levels were calculated using the 2^−ΔΔCT^ method^65^. The mean values and standard errors were calculated based on expression data of three biological replicates.

### Quantifying the digital expression of genes and analyzing the interaction between *FERR-R* and *FERR*

To quantifying the digital expression of genes and to explore gene regulation patterns, Illumina sequencing experiments (Illumina NovaSeq 6000, USA) were performed at levels of mRNA, lncRNA, small RNA and DNA methylation. RNA-Seq was performed to quantify the expression of the four protein-coding genes (*MET1*, *CLC*, *TCP*, and *FERR*); whereas strand-specific lncRNA-Seq was applied for measuring the transcription levels of the two non-protein-coding genes (*FERR-R*, *MSL*), as lncRNA-Seq worked for transcripts with and without polyA tails. The samples applied for sequencing were listed in Supplementary Table 8. Libraries were constructed using TruSeq RNA Library Prep Kit v2 (mRNA), TruSeq Stranded Total RNA Library Prep Kit(lncRNA), TruSeq Small RNA Library Preparation Kit (small RNA), and TruSeq Methyl Capture EPIC Library Prep Kit (DNA methylation) following the manufacturers’ instructions. The quantitative analysis processes of Illumina reads are described in Supplementary Method 4. The assembly of *MSL* sequences for *Populus deltoides* trees using Illumina sequencing reads was described in Supplementary Method 5.

### Experimental verification of interaction between *FERR-R* and *FERR*

Isolation of *Populus* mesophyll protoplasts was performed using the PEG-Mediated plant protoplast transformation kit (Shanghai Maokang Biotechnology, China) with 3% (w/v) cellulose R10 (Yakult Pharmaceutical, Japan) and 0.8% (w/v) macerozyme R10 (Yakult Pharmaceutical, Japan). The middle section of expanded *Populus* leaves (micropropagated *Populus* clone ‘Nanlin 895’) were cut into 0.5–1 mm fine strips, digested for 30 min in the dark using a desiccator for Vacuum infiltrate, and succeeded with digestion in the dark for 5 h without shaking^66^. The protoplasts were harvested by filtering through a 70-μm pore nylon cloth and then suspended in the transfection buffer.

The sequence of one siRNA generated from *FERR-R* locus was designed onto the backbone of AtMIR172a, driven by CaMV 35S promoter. The artificial miRNA generated in the construction mimics the siRNA of 21 nt from *FERR-R*. The artificial miRNA precursor sequence was synthesized on a Dr. Oligo384 (Biolytic, USA) and constructed into the vector p2GWF7 using Gateway technology. The full length of the *FERR* was amplified by using PrimeSTAR Max (TaKaRa, China) from cDNA of female flower bud. Site-directed synonymous mutagenesis of *FERR* was performed using the single-tube ‘megaprimer’ PCR method to generate the resistant version of *FERR* (referred to as *FERR*’). *FERR*’ transcript was synthesized with synonymous substitutions in the complementary sequences of artificial siRNA, which was also included in the experiment to test the functional specificity between *FERR-R* and *FERR*. Pro35S::PtFERR-GFP and Pro35S::FERR′-GFP were co-transfected with Pro35S::siFERR into *Populus* protoplasts. GFP fluorescence was captured using CarlZeiss LSM710 confocal microscope (Zeiss, Germany).

### Confirming the functions of *FERR-R* and *MSL* in transformed Arabidopsis

Binary vector p2301-35Splus was used in the transgenic experiments. The vector was created by sequentially cloning of *Hind*III-*Sma*I fragment and *EcoR*I-*Sac*I fragment of pBI121 (AF485783.1) into pCAMBIA2301 (AF234316). The genomic DNAs of *MSL* and CDS of *FERR* were separately cloned into p2301-35Splus. *Arabidopsis* ecotype Columbia-0 (Col-0) was grown under white LED light (Philips, Netherlands) with 16 h-light and 8 h-dark cycles at 19–23 °C until transformation. The binary construct was introduced into *Agrobacterium tumefaciens* strain GV3101 (pMP90) using a freezing method. *Arabidopsis* wild-type plants were transformed using the floral dip method^67^. Screening of transgenic plants was processed on 1/2 MS media containing 50 mg/mL kanamycin, and kanamycin-resistant transgenic seedlings were further confirmed by GUS staining. Wild type Col-0 and transgenic *Arabidopsis* plants were grown in a growth chamber at 23 °C/15 °C day/night temperatures under a 16 h/8 h light/dark cycle. Fresh flowers at different developmental stages were collected for microscopic observation with an Olympus SZX10 (Olympus, Japan) when plants were 7 weeks old. Floral stages were defined according to Smyth et al.^68^. For each stage, five flowers were collected from the main inflorescence of the same plant for phenotype observation. The flower samples of transgenic plants and wild-type controls were also collected for RNA-Seq experiments. The differential expressed analysis in *A. thaliana* was performed using the same pipeline as for *Populus*. The reads were mapped to the *A. thaliana* genome sequence Araport11 (https://araport.org/). Four and eight biological replicates were collected for *FERR* and *MSL* transgenic plants, respectively. For each *FERR* or *MSL* transgenic experiment, three biological replicates of *A. thaliana* Col-0 plants were collected as wild-type controls. The results of these RNAseq data are summarized in Supplemental Data 1 and Data 3. The raw data have been deposited NCBI SRA under accession PRJNA659408.

### Study the sex determination in *P. davidiana*

To explore the sex determination in *P. davidiana*, we sequenced the genome of a male tree using PacBio Sequel II (PacBio, USA) and assembled the genome following the same pipeline as that for *P. deltoides*. We then performed genome resequencing for the GWAS samples of *P. davidiana* and detected the YHS in males of *P. davidiana* by using rb-GWAS analysis. Sequence annotation and evolutionary analysis for duplicated genes were performed following the same pipelines as that of *P. deltoides*.

### Reporting summary

Further information on research design is available in the Nature Research Reporting Summary linked to this article.

## Supplementary information

Supplementary Information

Peer Review File

Reporting Summary

Description of Additional Supplementary Files

Supplementary Data 1

Supplementary Data 2

Supplementary Data 3

## Data Availability

Data supporting the findings of this work are available within the paper and its Supplementary Information files. A reporting summary for this Article is available as a Supplementary Information file. The datasets and plant materials generated and analyzed during the current study are available from the corresponding author upon request. All the raw sequences are deposited to NCBI SRA under the following accessions: BioProject PRJNA598948 (genome sequence of *P. deltoides*: female tree), PRJNA599215 (genome sequence of *P. deltoides*: male tree), PRJNA628142 (genomic resequencing of *P. deltoides* natural population); PRJNA599218 (gene expression and regulation data of *P. deltoides*), PRJNA628187 (genome sequence of *P. davidiana*: male tree), PRJNA628188 (genomic resequencing *P. davidiana*), PRJNA628368 (gene expression data of *P. davidiana*), and PRJNA659408 (gene expression data of *Arabidopsis thaliana*). The genome assembly sequences have been deposited in NCBI WGS under the following accessions: JABCQW000000000 (*P. deltoides* female tree), JABEKP000000000 (*P. deltoides* male tree), and JABEKQ000000000 (*P. davidiana* male tree). The following databases are used in this study: KEGG (https://www.kegg.jp/kegg/download/), KOG (https://www.ncbi.nih.gov/pub/COG/KOG), NR (https://www.ncbi.nlm.nih.gov/blast/db/), SILVA rRNA database (https://www.arb-silva.de, release 104), TrEMBL (https://www.uniprot.org/pub/databases/), and tRNAdb (http://trna.bioinf.uni-leipzig.de/DataOutput/). The source data underlying Figs. 1d and 3c, as well as Supplementary Fig. 8c are provided as a Source Data file. Source data are provided with this paper.

## Source data

Source Data

## Code availability

The command lines and codes used in GWAS are available through https://github.com/liangjiaoxue/PoplarSLR (10.5281/zenodo.4076814)^69^.

## References

[1] Renner SS (2014). The relative and absolute frequencies of angiosperm sexual systems: dioecy, monoecy, gynodioecy, and an updated online database. Am. J. Bot..

[2] Liu Z (2004). A primitive Y chromosome in papaya marks incipient sex chromosome evolution. Nature.

[3] Yin T (2008). Genome structure and emerging evidence of an incipient sex chromosome in Populus. Genome Res..

[4] Akagi T, Henry IM, Tao R, Comai L (2014). A Y-chromosome-encoded small RNA acts as a sex determinant in persimmons. Science.

[5] Harkess A (2017). The asparagus genome sheds light on the origin and evolution of a young Y chromosome. Nat. Commun..

[6] Westergaard M (1958). The mechanisms of sex determination in dioecious flowering plants. Adv. Genet..

[7] Charlesworth B, Charlesworth D (1978). A model for the evolution of dioecy and gynodioecy. Am. Nat..

[8] Akagi T (2018). A Y-encoded suppressor of feminization arose via lineage-specific duplication of a cytokinin response regulator in kiwifruit. Plant Cell.

[9] Akagi T (2019). Two Y-chromosome-encoded genes determine sex in kiwifruit. Nat. Plants.

[10] Dellaporta SL, Calderon-Urrea A (1994). The sex determination process in maize. Science.

[11] Boualem A (2015). A cucurbit androecy gene reveals how unisexual flowers develop and dioecy emerges. Science.

[12] Akagi T, Charlesworth D (2019). Pleiotropic effects of sex-determining genes in the evolution of dioecy in two plant species. Proc. R. Soc. B.

[13] Markussen T, Pakull B, Fladung M (2007). Positioning of sex-correlated markers for Populus in a AFLP-and SSR-Marker based genetic map of Populus tremula×tremuloides. Silvae Genet..

[14] Gaudet M (2008). Genetic linkage maps of *Populus nigra* L. including AFLPs, SSRs, SNPs, and sex trait. Tree Genet. Genomes.

[15] Pakull B (2009). Genetic linkage mapping in aspen (*Populus tremula* L. and *Populus tremuloides* Michx.). Tree Genet. Genomes.

[16] Paolucci I (2010). Genetic linkage maps of *Populus alba* L. and comparative mapping analysis of sex determination across Populus species. Tree Genet. Genomes.

[17] Pakull B (2011). Genetic mapping of linkage group XIX and identification of sex-linked SSR markers in a *Populus tremula* × *Populus tremuloides* cross. Can. J. For. Res..

[18] Tuskan GA (2012). The obscure events contributing to the evolution of an incipient sex chromosome in *Populus*: a retrospective working hypothesis. Tree Genet. Genomes.

[19] Hou J (2015). Different autosomes evolved into sex chromosomes in the sister genera of *Salix* and *Populus*. Sci. Rep..

[20] Pucholt P, Rönnberg-Wästljung AC, Berlin S (2015). Single locus sex determination and female heterogamety in the basket willow (*Salix viminalis* L.). Heredity.

[21] Pucholt P, Wright AE, Conze LL, Mank JE, Berlin S (2017). Recent sex chromosome divergence despite ancient dioecy in the willow Salix viminalis. Mol. Biol. Evol..

[22] Zhou R (2020). A willow sex chromosome reveals convergent evolution of complex palindromic repeats. Genome Biol..

[23] Almeida P (2020). Genome assembly of the basket willow, *Salix viminalis*, reveals earliest stages of sex chromosome expansion. BMC Biol..

[24] Kersten B (2014). The sex-linked region in *Populus tremuloides* Turesson 141 corresponds to a pericentromeric region of about two million base pairs on *P. trichocarpa* chromosome 19. Plant Biol..

[25] Song Y (2013). Sexual dimorphic floral development in dioecious plants revealed by transcriptome, phytohormone, and DNA methylation analysis in *Populus tomentosa*. Plant Mol. Biol..

[26] Bräutigam K (2017). Sexual epigenetics: gender-specific methylation of a gene in the sex determining region of Populus balsamifera. Sci. Rep..

[27] Song Y, Tian M, Ci D, Zhang D (2015). Methylation of microRNA genes regulates gene expression in bisexual flower development in andromonoecious poplar. J. Exp. Bot..

[28] Geraldes A (2015). Recent Y chromosome divergence despite ancient origin of dioecy in poplars (*Populus*). Mol. Ecol..

[29] Müller NA (2020). A single gene underlies the dynamic evolution of poplar sex determination. Nat. Plants.

[30] Hellmann E, Gruhn N, Heyl A (2010). The more, the merrier: cytokinin signaling beyond Arabidopsis. Plant Signal. Behav..

[31] Vatén A, Soyars CL, Tarr PT, Nimchuk ZL, Bergmann DC (2018). Modulation of asymmetric division diversity through cytokinin and SPEECHLESS regulatory interactions in the Arabidopsis stomatal lineage. Dev. Cell.

[32] Chang J (2019). Asymmetric distribution of cytokinins determines root hydrotropism in Arabidopsis thaliana. Cell Res..

[33] Srivastava AK, Dutta S, Chattopadhyay S (2019). MYC2 regulates ARR16, a component of cytokinin signaling pathways, in Arabidopsis seedling development. Plant Direct.

[34] Tuskan GA (2006). The genome of black cottonwood, *Populus trichocarpa* (Torr. & Gray). Science.

[35] Diggle PK (2011). Multiple developmental processes underlie sex differentiation in angiosperms. Trends Genet..

[36] Matzke MA, Mosher RA (2014). RNA-directed DNA methylation: an epigenetic pathway of increasing complexity. Nat. Rev. Genet..

[37] Sanderson BJ, Wang L, Tiffin P, Wu Z, Olson MS (2019). Sex-biased gene expression in flowers, but not leaves, reveals secondary sexual dimorphism in *Populus balsamifera*. New Phytol..

[38] Zhou R (2020). Sequencing and analysis of the sex determination region of *Populus trichocarpa*. Genes.

[39] Kapusta A (2013). Transposable elements are major contributors to the origin, diversification, and regulation of vertebrate long noncoding RNAs. PLoS Genet..

[40] Johnson R, Guigó R (2014). The RIDL hypothesis: transposable elements as functional domains of long noncoding RNAs. RNA.

[41] Wang J (2014). Primate-specific endogenous retrovirus-driven transcription defines naive-like stem cells. Nature.

[42] Zhao T (2018). LncRNAs in polyploid cotton interspecific hybrids are derived from transposon neofunctionalization. Genome Biol..

[43] Carlevaro-Fita J (2019). Ancient exapted transposable elements promote nuclear enrichment of human long noncoding RNAs. Genome Res..

[44] Wang M (2020). Phylogenomics of the genus Populus reveals extensive interspecific gene flow and balancing selection. New Phytol..

[45] Jones-Rhoades MW, Bartel DP, Bartel B (2006). MicroRNAs and their regulatory roles in plants. Annu. Rev. Plant Biol..

[46] Li XG (2010). Cytokinin overproduction-caused alteration of flower development is partially mediated by CUC2 and CUC3 in Arabidopsis. Gene.

[47] Zhang, H. et al. Recent origin of an XX/XY sex-determination system in the ancient plant lineage Ginkgo biloba. *BioRxiv*10.1101/517946 (2019).

[48] Charlesworth B, Charlesworth D (2000). The degeneration of Y chromosomes. Philos. Trans. R. Soc. Lond. B.

[49] Charlesworth D, Charlesworth B, Marais G (2005). Steps in the evolution of heteromorphic sex chromosomes. Heredity.

[50] Wang J (2012). Sequencing papaya X and Yh chromosomes reveals molecular basis of incipient sex chromosome evolution. Proc. Natl Acad. Sci. USA.

[51] VanBuren R (2015). Origin and domestication of papaya Yh chromosome. Genome Res..

[52] Pilkington SM (2019). Genetic and cytological analyses reveal the recombination landscape of a partially differentiated plant sex chromosome in kiwifruit. BMC Plant Biol..

[53] Torres MF (2018). Genus-wide sequencing supports a two-locus model for sex-determination in Phoenix. Nat. Commun..

[54] Huu CN (2016). Presence versus absence of CYP734A50 underlies the style-length dimorphism in primroses. Elife.

[55] Shore JS (2019). The long and short of the S-locus in Turnera (Passifloraceae). New Phytol..

[56] Piferrer F (2013). Epigenetics of sex determination and gonadogenesis. Dev. Dyn..

[57] Parkinson SE, Gross SM, Hollick JB (2007). Maize sex determination and abaxial leaf fates are canalized by a factor that maintains repressed epigenetic states. Dev. Biol..

[58] Chuck G, Meeley R, Irish E, Sakai H, Hake S (2007). The maize tasselseed4 microRNA controls sex determination and meristem cell fate by targeting Tasselseed6/indeterminate spikelet1. Nat. Genet..

[59] Golicz AA, Bhalla PL, Singh MB (2018). lncRNAs in plant and animal sexual reproduction. Trends Plant Sci..

[60] Yin T-M, Wang X-R, Andersson B, Lerceteau-Köhler E (2003). Nearly complete genetic maps of *Pinus sylvestris* L. (Scots pine) constructed by AFLP marker analysis in a full-sib family. Theor. Appl. Genet..

[61] Garrison, E. & Marth, G. T. Haplotype-based variant detection from short-read sequencing. *arXiv* arXiv:1207.3907 (2012).

[62] Purcell S (2007). PLINK: a tool set for whole-genome association and population-based linkage analyses. Am. J. Hum. Genet..

[63] Zhou X, Stephens M (2012). Genome-wide efficient mixed-model analysis for association studies. Nat. Genet..

[64] Brunner AM, Yakovlev IA, Strauss SH (2004). Validating internal controls for quantitative plant gene expression studies. BMC Plant Biol..

[65] Livak KJ, Schmittgen TD (2001). Analysis of relative gene expression data using real-time quantitative PCR and the 2-ΔΔCT method. Methods.

[66] Yoo S-D, Cho Y-H, Sheen J (2007). Arabidopsis mesophyll protoplasts: a versatile cell system for transient gene expression analysis. Nat. Protoc..

[67] Clough SJ, Bent AF (1998). Floral dip: a simplified method for Agrobacterium-mediated transformation of Arabidopsis thaliana. Plant J..

[68] Smyth DR, Bowman JL, Meyerowitz EM (1990). Early flower development in Arabidopsis. Plant Cell.

[69] Xue, L. et al. Evidence for roles of two Y-specific genes in sex determination in Populus deltoides. *Github*10.5281/zenodo.4076814 (2020). 10.1038/s41467-020-19559-2 PMC7674411 33208755

