# Supplementary material for: Evidences for a role of two Y-specific genes in sex determination in Populus deltoides
Source: Nat Commun. 2020 Nov 18;11:5893. doi: 10.1038/s41467-020-19559-2 (PMC7674411; doi:10.1038/s41467-020-19559-2)
Supplement: Supplementary file 1 — Supplementary Information [file 41467_2020_19559_MOESM1_ESM.pdf]

Evidences for a role of two Y-specific genes in sex determination in *Populus deltoides*

Xue *et al.*

Supplementary Method 1. Sequencing and assembly of *Populus deltoides* genomes

For PacBio sequencing, high molecular weight DNA was extracted using the cetyltrimethylammonium bromide method[1]. g-TUBE (Covaris, USA) was used to shear DNAs of the female and male separately into fragments with an average size of 20 kb. The PacBio SMRT libraries were constructed using sheared DNAs, then sequenced following standard protocols (PacBio, USA). The initial assemblies of PacBio reads of the female were generated by using Wtdbg v2.5[2], Falcon v0.3.0[3], and Canu v1.8 softwares[4]. Quickmerge v0.2[5] was applied to combine the assemblies mainly based on Wtdbg assembly. The resulted sequences were further polished using Illumina genomic resequencing reads with Pilon v1.23 software[6]. Hi-C libraries were constructed for the female parent following the Proximo™ Hi-C plant protocol (Phase Genomics, USA) and applied for sequencing on an Illumina HiSeq X platform (Illumina, USA). The HindIII restriction enzyme was used for library construction and read pair filtering. Interactions were identified using HiC-Pro v2.10.0[7]. Uniquely mapped paired reads were screened using HiC-Pro's default parameters and grouped into several categories based on 1) distance between read ends, 2) mapping directions of read ends, and 3) distance of read ends to restriction enzyme sites. We excluded the following categories of interaction pairs 'dangling ends', 'self-circle ligation', 're-ligation', and 'dumped pairs (outside of the expected range)'. The remaining reads were considered 'valid' pairs and applied for genome scaffolding using Lachesis v20171221[8]. BUSCO v3.0.2[9] was used to evaluate the completeness of the genome assemblies (Supplementary Table 2) with plant conserved single-copy gene set. Default settings of the parameters were used for the software if not specified.

Supplementary Method 2. Genome annotation

A *de novo* repeat library was constructed using LTR_retriever v2.8.7[10], RepeatScout v1.0.5[11], and PILER-DF v2.4[12]. The resulted repeat elements were categorized using PASTEClassifier v1.0[13] and combined with Repbase[14], which were further imported to RepeatMasker (version 4.07)[15] to identify and cluster repetitive elements. Sequences with more than ten monomers simple repeats 'CCCTAAA' were identified as telomeres.

Evidence of multiple resources was used to predict protein-coding genes in the genome. RNASeq data were mapped to the reference genome using HISAT2 v2.0.5[16] and assembled into transcripts using Stringtie v1.3.6.[17]. The resulted transcripts were screened using TransDecoder v5.0.2[18] and GeneMarkS-T v5.1[19] for protein-coding genes. PASA v2.0.2[20] was used to predict gene structures of the transcripts. The *ab initio* prediction was performed using Genscan v1.0[21], Augustus v3.2.3[22], and SNAP v2013-11-29[23]. GeMoMa v1.5.3[24] was applied for homology-based predictions. All the annotations were integrated using EVM v1.1.1[25]. Infenal v1.1.2[26] was used to predict rRNAs using Rfam as reference. The tRNA genes were predicted using tRNAscan-SE v1.3.1[27]. Pseudogenes were predicted using GeneWise v2.4.1[28] after the filtering of protein-coding genes using GenBlastA v1.0.4[29]. The functions of genes were annotated through similarity search of databases including NR, KOG, KEGG, and TrEMBL. During genome annotation, default settings of software were applied expecting the limit of intron length if needed. The minimum and maximum of intron length were set as 20 bp and 10,000 bp respectively.

Supplementary Method 3. Constructing the SLR haplotypes

To compare the sequences in SLR between the X and Y chromatids, we reconstructed the haplotypes of SLR-X and SLR-Y as follows: Canu software[4] was applied to assemble the PacBio reads of the sequenced male. The purge_haplotigs tool v1.0.3[30] was used to screen primary contigs and allelic secondary contigs. With the sequenced female as reference, both the primary and the secondary contigs were mapped to the reference genome. The contigs located in SLR were assigned to X and Y haplotypes based on SNPs between contigs and the reference genome.

Supplementary Method 4. Quantitative analysis of Illumina reads

Before applying to the corresponding analysis pipeline, low-quality Illumina reads were removed, and adaptor sequences were trimmed using Trimmonatic v0.36[31]. In data analysis of RNASeq, lncRNASeq, and sRNASeq, the rRNA/tRNA contaminants were also removed by mapping of the reads to rRNA/tRNA sequences from public databases. The rRNA sequences of all plant species were downloaded from SILVA rRNA database (https://www.arb-silva.de， release 104), and the tRNA sequences of *Arabidopsis thaliana* and *P. trichocarpa* were downloaded from tRNAdb (http://trna.bioinf.uni-leipzig.de/DataOutput/). STAR v2.5.3a[32] was used to map the RNASeq and lncRNASeq reads onto the reference with setting '--alignMatesGapMax 20000 --alignIntronMax 10000', and DEseq2[33] was applied for differential expression analysis. The numbers of differentially expressed genes are shown in supplemental Table 7. Transcripts of lncRNA were assembled using Trinity V2.6.6[19] with the parameter settings for strand-specific reads ('--SS_lib_type RF'). The obtained sequences were applied to search NR database. Small RNA reads and Bisulfite sequencing (BS-seq) reads were mapped

onto genome reference using Bowtie2 v2.3.4.1[34] and Bismark v0.16.3[35], respectively. The alignment results were visualized using IGV v 2.4.14[36].

Supplementary Method 5. Assembly of *MSL* sequences for *Populus deltoides* population

To further confirm the specificity of *MSL* locus, the raw reads mapping the *MSL* locus were extracted from the bam files of all female and male individuals in the GWAS population. Contigs of each individual plant were further assembled using Velvet v1.2.10[37] and aligned with *MSL* sequence from PacBio assembly. The alignment results were visualized in using R package genoPlot v0.8.9[38] and shown in supplementary Fig. 8a and 8b.

Supplementary Table 1. Primers used in this study.

Primer	Forward primer sequence	Reverse primer sequence	Application
N025	CCCCCTTTAAATTTTACTGTAG	TTTTCTTGTTGAGTTGACGTT	Fine mapping
N062	TCCTGAATAGTACGCTACACA	ATTGATTGAAGGGCATACATA	Fine mapping
N084	CTGCTACAACTCCTCCAAATA	GTGACTCAGAAAACCCATAAA	Fine mapping
N110	AAAGACATTCCCTTTCTTGTC	GGGTTGTTTCCTTTTCCTT	Fine mapping
N126	TTTTTCTCTCTCACAAATGGA	CATTGACTATAATGCGGGTAG	Fine mapping
N194	TACCTCTCGAGTTGAAACAGA	AATGCATGAGGCAATAGTTAC	Fine mapping
N209	ATTCCAACACCAACTAACTCA	GACCCTCATTGTCATGGAT	Fine mapping
N362	TACATTGAATCAGCTCCAAAC	ATTCAAAGTTCAGGGATGAAA	Fine mapping
N283	ATCATCTGTTTTTACGGTTCA	TTAATAGGGAGTTGGAGGAAG	Fine mapping
N293	ATCCAAGTCCATTTTTCTTTC	CCTGTCTTTCTTTCGTTTGTA	Fine mapping
F_841	ATAAATCCTTACGACCGCCAG	GCCAGATCTAACTCACGCAGG	Clone of SLR
F_815	AACGAGTGCTTGAGTCAGAAA	AGGAGAGAGAAAGGGTGGCTA	Clone of SLR
F_817	CCACCGACAAGAGACAAAGAA	TGTCTGAACAAAACGCCATTAC	Clone of SLR
F_821	TGTTGATACGGATGACCACCT	CGGGATAACCCAATAAAAAGTA	Clone of SLR
F_651	AGAACTATCTCCTCCCCTGCG	CCGTGTGTTGTCTGTGTGGAG	Clone of SLR
F_709	ATTGCTGATCTTTTCGGTCTTT	AAATCACTTTTGTGAATTTCAGC	Clone of SLR
F_655	GAGAGTCTGGTACAGTATGCTGT	AACCACAAGTCGTTCAAGTATC	Clone of SLR
F_710	GTATGACAATCCCTCTCTCCCA	ATTTCTATTAGAGAGGCCCACG	Clone of SLR
F_657	TGTTGTGTCGCTTATGACCAC	CTGGCGGTTATGTGAGTATTG	Clone of SLR
F_661	CCACACAATACTCACATAACCGC	TTAGCAATTATCTCCGCCTCAT	Clone of SLR
F_712	AAAGAATGCCCCAAGGAGAAC	TACTTGTGTTGAGAAGAGGGGG	Clone of SLR
F_823	GCTGAAGCGTGACTCGATCAG	AAAGACTTCATCTCGCACACTTG	Clone of SLR
F_827	AGAAAGACGCATAACAACGGTG	GAACTGTGAGCGTCGTGTGAA	Clone of SLR
F_830	AGGACATCTAAACTCCCAGCT	TTCTTGTAGCCATCAATCTGC	Clone of SLR
F_833	AGAGAGCCAGTTTTTTACGAAT	CAGAAAGATAAGGAGCAACAACT	Clone of SLR
FERR	GTGTTTTGTTGAGGAGATTAGAC	CCCTTCTTGTCTCTCTTTCTG	Clone of *FERR*
MSL	TTATTGTAGAAATAAGGCCTATATTCG	AAATTTATTTATAACGATCATTATCTCTCT	Clone of *MSL*
P801	GTTGAGAGGTATGTGGTAGTGC	GTGTTTTCTCTACCACCACCTC	qPCR (*MET*)
P806	GAGAGAACCAGACAAATGCGG	CATCCCCAATTTTCTTAACCTC	qPCR (*TCP*)
P977	TTGCTATCCCATCTGGACTTT	CTTGTTGAAGTTATCAGCCACG	qPCR (*CLC*)
P809	AAACGGAAAGAGAGCATTGGA	TCATAACCTGTCATTCCTGGCA	qPCR (*FERR*)
P982	TCCTTGGTGTCTGAAAGTGTC	GGGAACCTTTGAAAAGTTGAA	qPCR (*MSL*)
P990	AAGTAGCAATAGAGGTGGCGA	CTCTAACCCAACAGCACATCTT	qPCR (*FERR-R*)
PtUBQ	GTTGATTTTTGCTGGGAAGC	GATCTTGGCCTTCACGTTGT	ubiquitin
FERR-infusion	GACTCTAGAGGATCCATGGCCAGCTCTTCTTCTTCC	GGAAATTCGAGCTCGGTACCTTATGCATGTTCGTCTTCTCCTGCAA	*FERR*_p2301-35S plus
MSL-infusion	GGGGACTCTAGAGGATCCTTATTGTAGAAATAAGGCCTATATTCG	GAAATTCGAGCTCGGTACCAAATTTATTTATAACGATCATTATCTCTCT	*MSL*_p2301-35S plus

Supplementary Table 2. Genes annotated on SLR-X and SLR-Y, showing genes whose annotation suggests that they are XY pairs of genes.

X haplotype						Y haplotype				
Pairs[a]	Start	End	Dire[b]	Gene ID	Annotation	Start	End	Dire[b]	Gene ID	Annotation
1	6516	10142	-	EVM0038001	Putative ribonuclease H protein At1g65750	24808	28035	-	EVM0039001	Putative ribonuclease H protein At1g65750
						28452	29411	-	EVM0039002	None
						34621	42229	-	EVM0039003	Putative ribonuclease H protein At1g65750
	10186	11322	-	EVM0038002	None					
						43815	49568	-	EVM0039004	None
						50846	53313	-	EVM0039005	*MSL*
	13949	20791	-	EVM0038003	Membrane protein of ER body-like protein					ABSENT FROM Y 1
	20888	41226	+	EVM0038004	Adenosine deaminase-like protein					ABSENT FROM Y 2
	42680	43414	-	EVM0038005	None					ABSENT FROM Y 3
	43502	44999	-	EVM0038006	Probable serine/threonine-protein kinase PBL23					ABSENT FROM Y 4
					ABSENT FROM X 1	61451	77472	-	EVM0039006	*FERR-R*
					ABSENT FROM X 2	81909	82697	+	EVM0039007	None
2	47938	51624	+	EVM0038007	T-complex protein 1 subunit gamma(TCP)	87483	94421	+	EVM0039008	T-complex protein 1 subunit gamma (TCP)
3	69860	73840	+	EVM0038008	Chloride channel protein CLC-c(CLC)	97911	101936	+	EVM0039009	Chloride channel protein CLC-c (CLC)
					ABSENT FROM X 3	102582	105723	+	EVM0039010	Transposon
4	80892	90908	+	EVM0038009	DNA (cytosine-5)-methyltransferase 1	114011	123903	+	EVM0039011	DNA (cytosine-5)-methyltransferase 1
	96020	99449	+	EVM0038010	Probable disease resistance protein At1g15890					ABSENT FROM Y 5
5	100609	103386	+	EVM0038011	Calcium-dependent protein kinase 4	129331	136656	+	EVM0039012	Calcium-dependent protein kinase 4

	105755	106240	-	EVM0038012	None					
	109496	110467	+	EVM0038013	None					
	113188	113559	+	EVM0038014	None					
						138996	139496	-	EVM0039013	None
						146409	146780	+	EVM0039014	None
6	113610	113873	+	EVM0038015	E3 ubiquitin-protein ligase makorin	146831	147094	+	EVM0039015	E3 ubiquitin-protein ligase makorin
	115866	116422	-	EVM0038016	None					
	116471	118068	-	EVM0038017	None					
7	118349	126441	+	EVM0038018	Cleft lip and palate transmembrane protein 1 homolog	147865	155841	+	EVM0039016	Cleft lip and palate transmembrane protein 1 homolog
8	128479	133608	-	EVM0038019	Protein SUPPRESSOR OF GENE SILENCING 3	157892	163032	-	EVM0039017	Protein SUPPRESSOR OF GENE SILENCING 3
9	136821	139171	-	EVM0038020	None	167821	168045	-	EVM0039018	None
10	142264	143104	+	EVM0038021	PHD finger protein At1g33420	172166	177190	+	EVM0039019	PHD finger protein At1g33420
	143296	144731	+	EVM0038022	None					
	145597	145828	-	EVM0038023	None					
	146378	146602	-	EVM0038024	None					
	150695	155648	+	EVM0038025	PHD finger protein At1g33420					
11	156184	159201	+	EVM0038026	Peroxidase 47	177652	180652	+	EVM0039020	Peroxidase 47
12	161059	164210	-	EVM0038027	4,5-DOPA dioxygenase extradiol	182505	185706	-	EVM0039021	4,5-DOPA dioxygenase extradiol
13	166480	180086	-	EVM0038028	Probable disease resistance protein At4g27220	188011	190086	-	EVM0039022	Probable disease resistance protein At4g27220
	185375	185461	+	EVM0038029	None					
	185494	186861	-	EVM0038030	None					

	188133	190558	-	EVM0038031	Probable LRR receptor-like serine/threonine-protein kinase At1g07650					ABSENT FROM Y 6
	190717	194232	-	EVM0038032	Putative disease resistance protein RGA4					ABSENT FROM Y 7
	198390	201014	+	EVM0038033	None					
	201032	201112	-	EVM0038034	None					
	201595	204301	-	EVM0038035	None					
	205006	206255	-	EVM0038036	None					
14	209242	212680	-	EVM0038037	Probable disease resistance protein At4g27220	197721	202988	-	EVM0039023	Probable disease resistance protein At4g27220
	218494	219026	-	EVM0038038	Probable LRR receptor-like serine/threonine-protein kinase At1g07650					ABSENT FROM Y 8
15	225393	229630	-	EVM0038039	Probable disease resistance protein At4g27220	214009	218246	-	EVM0039024	Probable disease resistance protein At4g27220
	230086	231963	-	EVM0038040	None					
						220236	227934	-	EVM0039025	None
16	241933	245026	+	EVM0038041	Probable disease resistance protein At5g43730	238511	241603	+	EVM0039026	Probable disease resistance protein At5g43730

[a] XY gene pairs
[b] Direction

Supplementary Table 3. Genome resequencing statistics of the female and male *P. deltoides*.

Sample ID	Sex	Read Base	Read Number	Coverage
36-1	Female	11,649,192,900	38,830,643	27.09
40-1	Female	11,168,084,700	37,226,949	25.97
42-4	Female	12,287,930,700	40,959,769	28.58
47-4	Female	12,636,568,200	42,121,894	29.39
48-5	Female	11,102,390,400	37,007,968	25.82
50-5	Female	14,602,650,900	48,675,503	33.96
52-1	Female	11,245,898,400	37,486,328	26.15
60-8	Female	13,177,485,900	43,924,953	30.65
63-1	Female	11,296,434,900	37,654,783	26.27
66-5	Female	11,477,944,500	38,259,815	26.69
70-5	Female	10,964,830,500	36,549,435	25.50
72-6	Female	11,179,380,300	37,264,601	26.00
74-4	Female	11,733,299,700	39,110,999	27.29
77-1	Female	10,556,599,800	35,188,666	24.55
78-4	Female	11,534,989,800	38,449,966	26.83
80-4	Female	11,815,474,500	39,384,915	27.48
83-3	Female	14,071,658,400	46,905,528	32.72
87-6	Female	10,132,395,000	33,774,650	23.56
88-2	Female	11,882,076,600	39,606,922	27.63
90-5	Female	13,220,877,300	44,069,591	30.75
92-7	Female	9,888,900,000	32,963,000	23.00
93-1	Female	11,695,936,500	38,986,455	27.20
96-7	Female	11,972,586,300	39,908,621	27.84
97-3	Female	11,297,386,500	37,657,955	26.27
98-5	Female	15,165,401,700	50,551,339	35.27
22-1	Female	12,758,267,700	42,527,559	29.67
24-1	Female	11,006,373,300	36,687,911	25.60
31-1	Female	11,898,806,700	39,662,689	27.67
28-3	Female	11,370,264,300	37,900,881	26.44
7-3	Female	11,660,032,200	38,866,774	27.12
11-1	Female	11,459,820,900	38,199,403	26.65
100-2	Female	12,408,186,000	41,360,620	28.86
101-5	Female	11,580,759,900	38,602,533	26.93
102-3	Female	11,684,571,900	38,948,573	27.17
103-3	Female	11,444,420,700	38,148,069	26.61
104-1	Female	11,093,350,500	36,977,835	25.80
61-18	Female	12,642,730,800	42,142,436	29.40
S3230	Female	14,264,241,300	47,547,471	33.17
81-22	Female	11,090,319,000	36,967,730	25.79

86-13	Female	11,822,777,400	39,409,258	27.49
89-18	Female	11,622,264,300	38,740,881	27.03
94-27	Female	11,794,462,200	39,314,874	27.43
95-30	Female	11,465,142,300	38,217,141	26.66
S3016	Female	12,946,626,300	43,155,421	30.11
S3109	Female	12,148,255,800	40,494,186	28.25
S3229	Female	13,144,587,900	43,815,293	30.57
S3107	Female	12,787,382,100	42,624,607	29.74
S3700	Female	15,085,499,100	50,284,997	35.08
S3406	Female	11,765,872,500	39,219,575	27.36
31-4	Male	12,545,672,100	41,818,907	29.18
35-2	Male	11,043,030,000	36,810,100	25.68
36-3	Male	11,442,942,900	38,143,143	26.61
45-1	Male	13,935,390,600	46,451,302	32.41
47-2	Male	11,552,744,700	38,509,149	26.87
48-1	Male	11,386,119,000	37,953,730	26.48
51-1	Male	12,487,357,200	41,624,524	29.04
60-2	Male	11,990,539,200	39,968,464	27.88
62-3	Male	11,821,741,500	39,405,805	27.49
63-5	Male	11,965,007,100	39,883,357	27.83
70-1	Male	11,459,258,100	38,197,527	26.65
72-2	Male	12,000,861,900	40,002,873	27.91
74-1	Male	14,045,237,100	46,817,457	32.66
77-10	Male	11,668,235,700	38,894,119	27.14
78-2	Male	11,339,813,400	37,799,378	26.37
86-6	Male	11,183,043,900	37,276,813	26.01
87-5	Male	11,769,492,300	39,231,641	27.37
88-7	Male	11,964,074,100	39,880,247	27.82
93-4	Male	11,028,985,200	36,763,284	25.65
95-12	Male	11,846,649,600	39,488,832	27.55
96-10	Male	13,193,655,900	43,978,853	30.68
28-2	Male	12,408,126,600	41,360,422	28.86
24-3	Male	11,172,411,300	37,241,371	25.98
22-4	Male	11,966,299,200	39,887,664	27.83
100-4	Male	11,177,231,400	37,257,438	25.99
101-3	Male	10,894,545,900	36,315,153	25.34
102-9	Male	11,939,396,100	39,797,987	27.77
103-9	Male	11,884,926,600	39,616,422	27.64
104-2	Male	12,839,783,400	42,799,278	29.86
131-3	Male	13,945,134,900	46,483,783	32.43
S3301	Male	11,829,934,500	39,433,115	27.51
65-18	Male	11,610,252,900	38,700,843	27.00
66-16	Male	11,715,514,500	39,051,715	27.25

81-30	Male	11,411,807,700	38,039,359	26.54
89-28	Male	11,875,062,600	39,583,542	27.62
94-28	Male	11,835,697,200	39,452,324	27.52
S3101	Male	13,373,461,500	44,578,205	31.10
S3201	Male	14,262,937,800	47,543,126	33.17
S3244	Male	11,973,510,900	39,911,703	27.85
S3261	Male	13,581,076,200	45,270,254	31.58
S3415	Male	11,210,397,600	37,367,992	26.07
S3702	Male	14,024,592,000	46,748,640	32.62
S3804	Male	13,570,024,800	45,233,416	31.56
S3236	Male	11,486,967,600	38,289,892	26.71
S3239	Male	14,794,821,300	49,316,071	34.41
S3240	Male	13,313,584,500	44,378,615	30.96

Supplementary Table 4. Summary of genome regions containing SNPs with fully sex-linked genotype configurations.

Chromosome	Start of region	End of region	Number of SNPs.	Number of genes
XIX SLR of XY pair	120,289	161,988	315	3
XIX PAR of XY pair	17,899,199	17,909,028	78	1 (FERR)
IX (autosome)	7,729,222	7,730,262	27	1 (HEMA1)
I	50,047,869	50,053,274	5	2
XVIII	4,573,272	4,573,288	2	1
I	22,376,091	—	1	0
Contig01665	13,437	16,183	7	2
Total	435	10		

Supplementary Table 5. Sequence homology analysis for sequence containing SEMSs.

Location of SEMSs	Length of query sequence	Hit start in the YHS	Hit end in the YHS	Identity (%)	E Value
PAR	10,030	61,590	61,418	92.49	6.42E-63
PAR	10,030	61,683	61,588	93.75	8.67E-32
PAR	10,030	61,767	63,351	87.59	0
PAR	10,030	66,854	63,357	88.95	0
PAR	10,030	66,965	67,466	88.76	7.11E-172
PAR	10,030	67,601	68,223	90.21	0
PAR	10,030	68,416	69,127	92.61	0
PAR	10,030	68,460	66,820	85.42	0
PAR	10,030	69,398	69,226	93.06	1.38E-64
PAR	10,030	69,425	73,224	88.61	0
PAR	10,030	74,662	74,993	94.29	2.67E-141
PAR	10,030	78,458	75,021	89.37	0
PAR	10,030	78,712	79,523	92.16	0
PAR	10,030	79,516	80,026	92.25	0
Chr_I	5,606	49,675	47,836	89.24	0
Chr_I	5,606	49,856	49,671	89.25	1.00E-58
Chr_I	201	53,477	53,286	89.45	1.45E-63
Chr_IX	1,241	73,287	74,402	89.26	0
Chr_XVIII	217	104,531	104,747	91.71	9.29E-81
Contig01665	2,947	106,608	103,675	93.49	0

Supplementary Table 6. List of samples used for sequencing in this study.

Sample	Tissue	Library_ID	Application
P. deltoides ♀	Flower bud	T2 (June 18)_bud_1_F	lncRNA-seq
P. deltoides ♀	Flower bud	T2 (June 18)_bud_2_F	lncRNA-seq
P. deltoides ♀	Flower bud	T2 (June 18)_bud_3_F	lncRNA-seq
P. deltoides ♀	Flower bud	T3 (July 3)_bud_1_F	lncRNA-seq
P. deltoides ♀	Flower bud	T3 (July 3)_bud_2_F	lncRNA-seq
P. deltoides ♀	Flower bud	T3 (July 3)_bud_3_F	lncRNA-seq
P. deltoides ♀	Flower bud	T4 (July 18)_bud_1_F	lncRNA-seq
P. deltoides ♀	Flower bud	T4 (July 18)_bud_2_F	lncRNA-seq
P. deltoides ♀	Flower bud	T4 (July 18)_bud_3_F	lncRNA-seq
P. deltoides ♀	Descaled flower bud	T5 (August 3)_bud_1_F	lncRNA-seq
P. deltoides ♀	Descaled flower bud	T5 (August 3)_bud_2_F	lncRNA-seq
P. deltoides ♀	Descaled flower bud	T5 (August 3)_bud_3_F	lncRNA-seq
P. deltoides ♀	Descaled flower bud	T8 (December 1)_bud_1_F	lncRNA-seq
P. deltoides ♀	Descaled flower bud	T8 (December 1)_bud_2_F	lncRNA-seq
P. deltoides ♀	Descaled flower bud	T8 (December 1)_bud_3_F	lncRNA-seq
P. deltoides ♂	Flower bud	T2 (June 18)_bud_1_M	lncRNA-seq
P. deltoides ♂	Flower bud	T2 (June 18)_bud_2_M	lncRNA-seq
P. deltoides ♂	Flower bud	T2 (June 18)_bud_3_M	lncRNA-seq
P. deltoides ♂	Flower bud	T3 (July 3)_bud_1_M	lncRNA-seq
P. deltoides ♂	Flower bud	T3 (July 3)_bud_2_M	lncRNA-seq
P. deltoides ♂	Flower bud	T3 (July 3)_bud_3_M	lncRNA-seq
P. deltoides ♂	Flower bud	T4 (July 18)_bud_1_M	lncRNA-seq
P. deltoides ♂	Flower bud	T4 (July 18)_bud_2_M	lncRNA-seq
P. deltoides ♂	Flower bud	T4 (July 18)_bud_3_M	lncRNA-seq
P. deltoides ♂	Descaled flower bud	T5 (August 3)_bud_1_M	lncRNA-seq
P. deltoides ♂	Descaled flower bud	T5 (August 3)_bud_2_M	lncRNA-seq
P. deltoides ♂	Descaled flower bud	T5 (August 3)_bud_3_M	lncRNA-seq
P. deltoides ♂	Descaled flower bud	T8 (December 1)_bud_1_M	lncRNA-seq
P. deltoides ♂	Descaled flower bud	T8 (December 1)_bud_2_M	lncRNA-seq
P. deltoides ♂	Descaled flower bud	T8 (December 1)_bud_3_M	lncRNA-seq
P. deltoides ♀	Flower bud	T2 (June 18)_bud_1_F	small RNA
P. deltoides ♀	Flower bud	T2 (June 18)_bud_2_F	small RNA
P. deltoides ♀	Flower bud	T2 (June 18)_bud_3_F	small RNA
P. deltoides ♀	Descaled flower bud	T8 (December 1)_bud_1_F	small RNA
P. deltoides ♀	Descaled flower bud	T8 (December 1)_bud_2_F	small RNA
P. deltoides ♀	Descaled flower bud	T8 (December 1)_bud_3_F	small RNA
P. deltoides ♀	Descaled flower bud	T9 (January 15)_bud_1_F	small RNA
P. deltoides ♀	Descaled flower bud	T9 (January 15)_bud_2_F	small RNA
P. deltoides ♀	Descaled flower bud	T9 (January 15)_bud_3_F	small RNA
P. deltoides ♂	Flower bud	T2 (June 18)_bud_1_M	small RNA

P. deltoides ♂	Flower bud	T2 (June 18)_bud_2_M	small RNA
P. deltoides ♂	Flower bud	T2 (June 18)_bud_3_M	small RNA
P. deltoides ♂	Descaled flower bud	T8 (December 1)_bud_1_M	small RNA
P. deltoides ♂	Descaled flower bud	T8 (December 1)_bud_2_M	small RNA
P. deltoides ♂	Descaled flower bud	T8 (December 1)_bud_3_M	small RNA
P. deltoides ♂	Descaled flower bud	T9 (January 15)_bud_1_M	small RNA
P. deltoides ♂	Descaled flower bud	T9 (January 15)_bud_2_M	small RNA
P. deltoides ♂	Descaled flower bud	T9 (January 15)_bud_3_M	small RNA
P. deltoides ♀	Flower bud	T3 (July 3)_bud_1_F	DNA methylation
P. deltoides ♀	Flower bud	T3 (July 3)_bud_2_F	DNA methylation
P. deltoides ♀	Flower bud	T3 (July 3)_bud_3_F	DNA methylation
P. deltoides ♀	Descaled flower bud	T8 (December 1)_bud_1_F	DNA methylation
P. deltoides ♀	Descaled flower bud	T8 (December 1)_bud_2_F	DNA methylation
P. deltoides ♀	Descaled flower bud	T8 (December 1)_bud_3_F	DNA methylation
P. deltoides ♀	Descaled flower bud	T9 (January 15)_bud_1_F	DNA methylation
P. deltoides ♀	Descaled flower bud	T9 (January 15)_bud_2_F	DNA methylation
P. deltoides ♀	Descaled flower bud	T9 (January 15)_bud_3_F	DNA methylation
P. deltoides ♂	Flower bud	T3 (July 3)_bud_1_M	DNA methylation
P. deltoides ♂	Flower bud	T3 (July 3)_bud_2_M	DNA methylation
P. deltoides ♂	Flower bud	T3 (July 3)_bud_3_M	DNA methylation
P. deltoides ♂	Descaled flower bud	T8 (December 1)_bud_1_M	DNA methylation
P. deltoides ♂	Descaled flower bud	T8 (December 1)_bud_2_M	DNA methylation
P. deltoides ♂	Descaled flower bud	T8 (December 1)_bud_3_M	DNA methylation
P. deltoides ♂	Descaled flower bud	T9 (January 15)_bud_1_M	DNA methylation
P. deltoides ♂	Descaled flower bud	T9 (January 15)_bud_2_M	DNA methylation
P. deltoides ♂	Descaled flower bud	T9 (January 15)_bud_3_M	DNA methylation

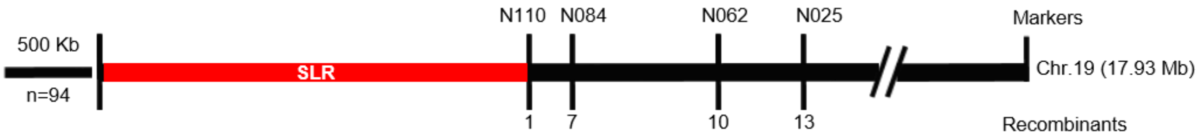

Supplementary Fig. 1. Initial mapping of sex-linked locus. A small population of 94 *P. deltoides* individuals was screened using SSR markers.

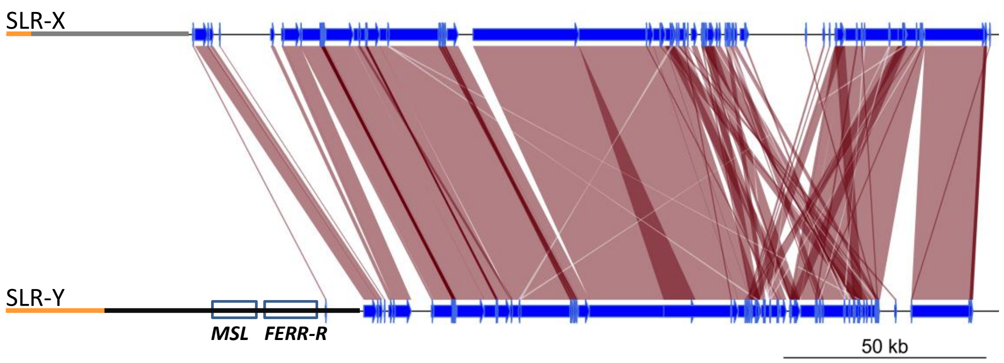

Supplementary Fig. 2. Alignment of sex-linked regions of the *P. deltoides* X and Y haplotypes. The orange lines at the left indicate telomere sequences. The blue blocks and arrows indicate sequencing showing similarity between two haplotypes. The red lines connect the regions of sequence similarity in the two haplotypes.

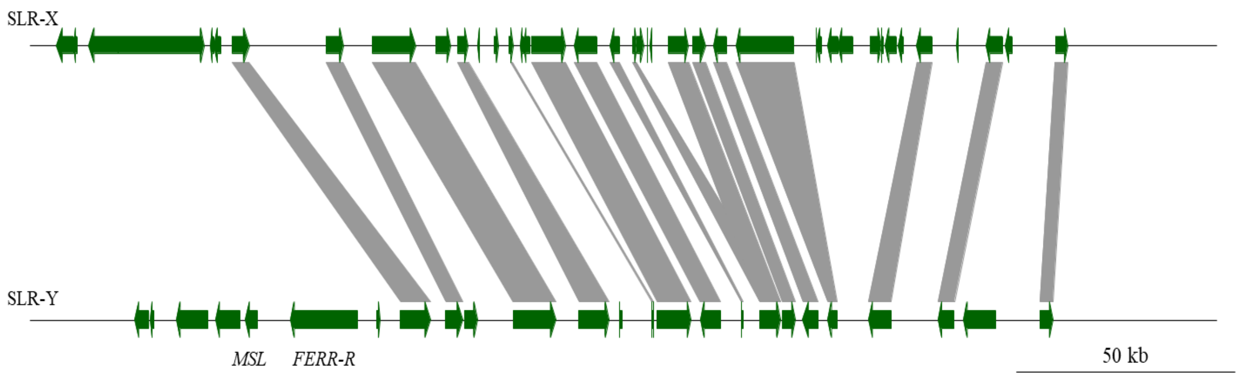

Supplementary Fig. 3. Synteny plot of the genes in haplotypes X and Y. Predicted gene models are shown in green arrows. The gray blocks indicate gene pairs between two haplotypes. The plot was generated using genoPlotR.

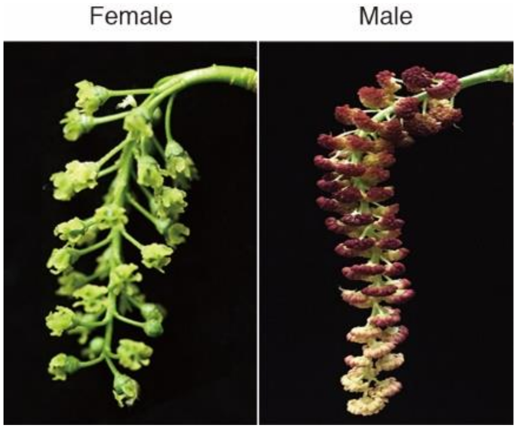

Supplementary Fig. 4. Female and male flowers of *P. deltoides*.

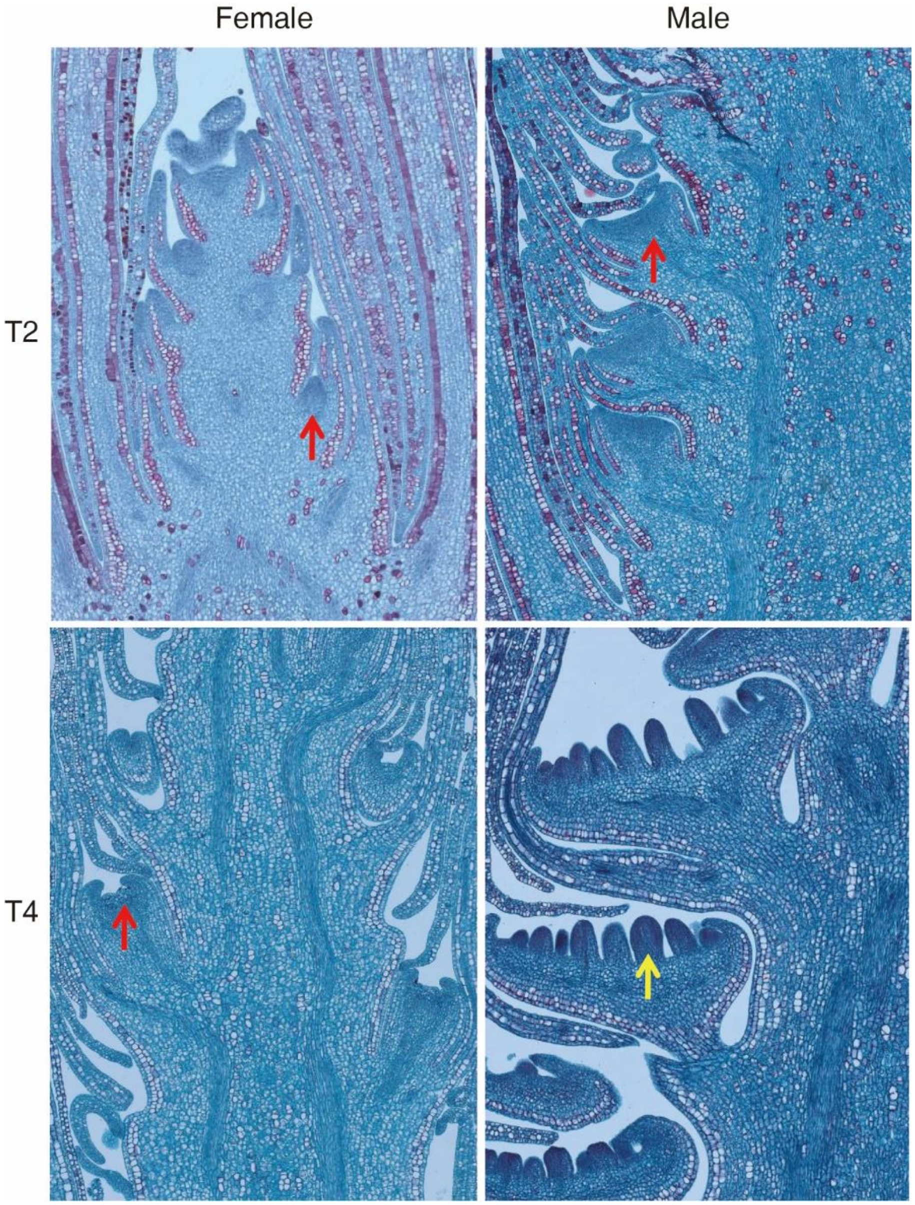

Supplementary Fig. 5. The longitudinal section of the male and female inflorescences at T2 and T4. T2 and T4 represent June 18 and July 18 respectively. The red arrows point to the florets primordium, whereas the yellow arrow denotes the anther primordium. Three independent samples were observed with similar results.

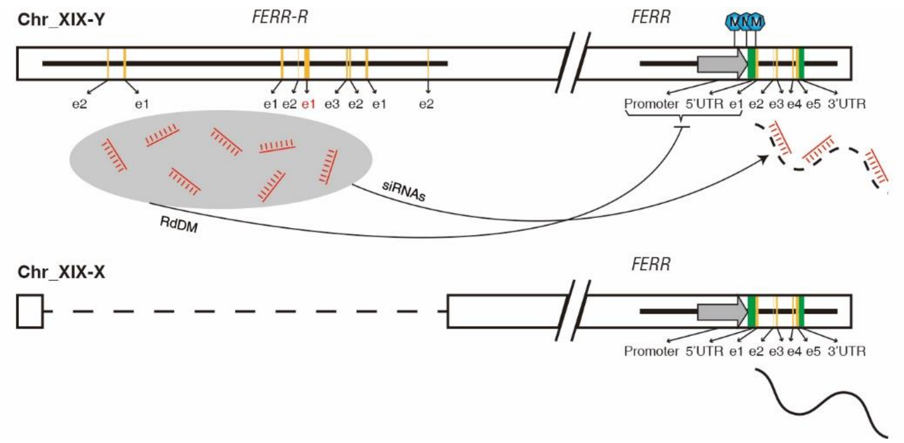

Supplementary Fig. 6. The proposed model for *FERR-R*. e1-e5 represent exons of the *FERR* gene. RdDM indicates RNA-directed DNA methylation, and ‘siRNAs’ indicates siRNA-guided mRNA cleavage. XX females do not undergo the cleavage, and female structures develop. XY males express the Y-linked *FERR-R* gene, and undergo the cleavage, suppressing development of female flowers.

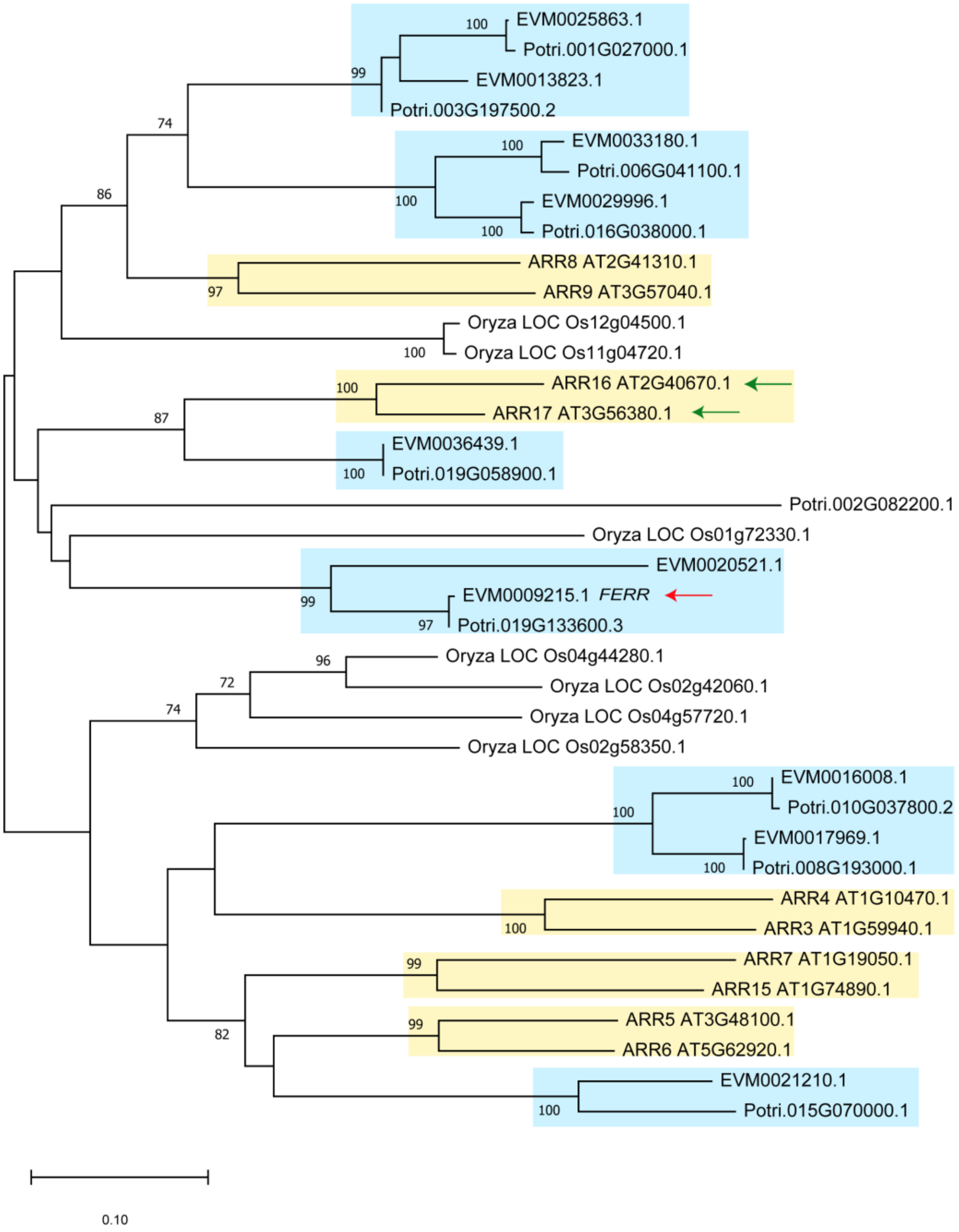

Supplementary Fig. 7. Phylogenetic analysis of type-A *RR* genes in several plants. Protein sequences of type-A *RR* genes of *P. deltoides* (indicated by names starting with EVM), *P. trichorcarpa* (names starting with Potri), *Arabidopsis thaliana* (names starting with ARR), and rice (names starting with *Oryza*) were used to construct the tree. A neighbor-joining tree was constructed using MEGA v. 10.1.7. The numbers by the branches indicate percentages out of 1,000 bootstrap replicates (only percentages higher than 70% are shown). *FERR* is indicated by a red arrow, and *ARR16* and *ARR17* by green arrows. *RR* genes of poplars and *A. thaliana* are indicated by blue and yellow boxes, respectively.

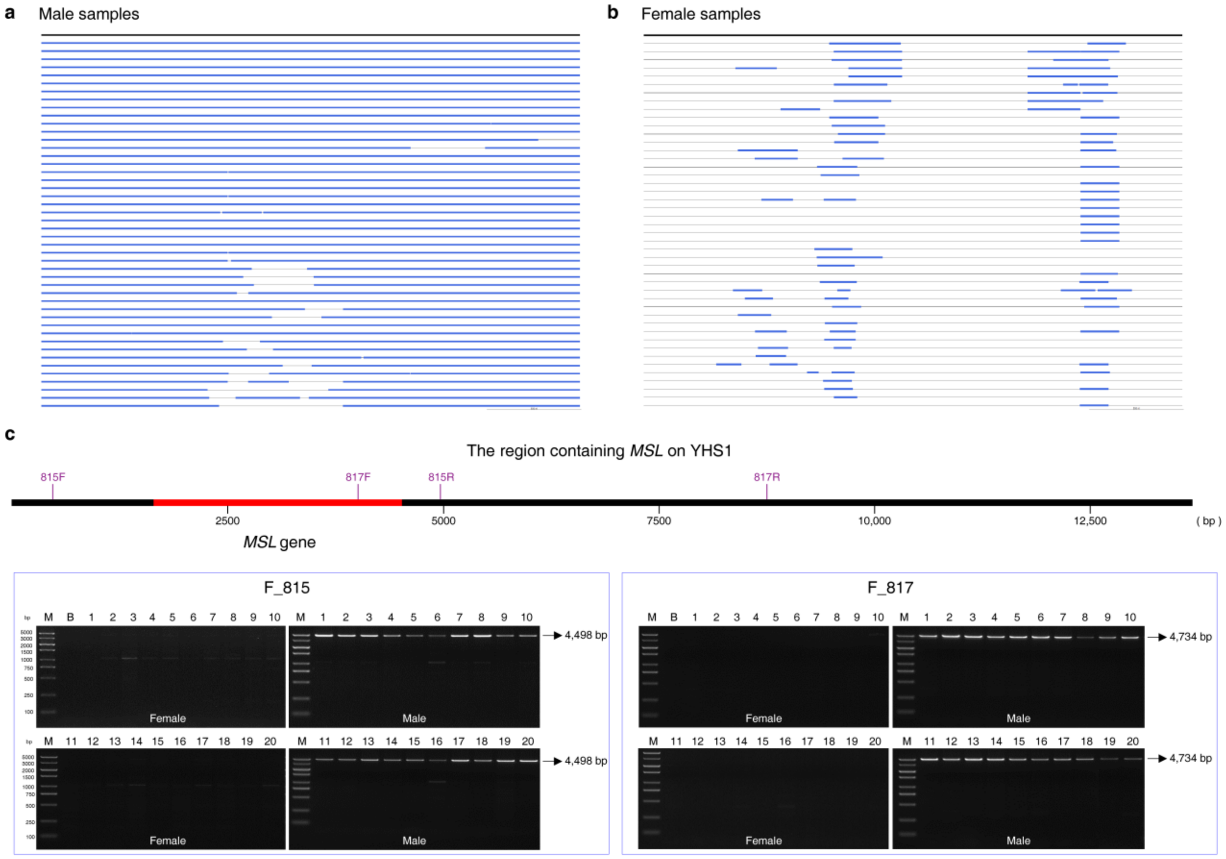

Supplementary Fig. 8. Assemblies of *MSL* sequences for the female and male GWAS samples. a Sequence assembly by raw reads from each of the males; **b** Sequence assembly by raw reads from each of the females. Raw reads matching *MSL* locus were extracted and assembled using Velvet. The derived contigs were aligned against the *MSL* sequence to show the matching positions. **c** Agarose gel electrophoresis profile for fragments F_815 and F_817 in females and males. Similar results were obtained in two independent experiments. M, molecular marker. B, blank control. Source data underlying Supplementary Figure 8c are provided as a Source Data file.

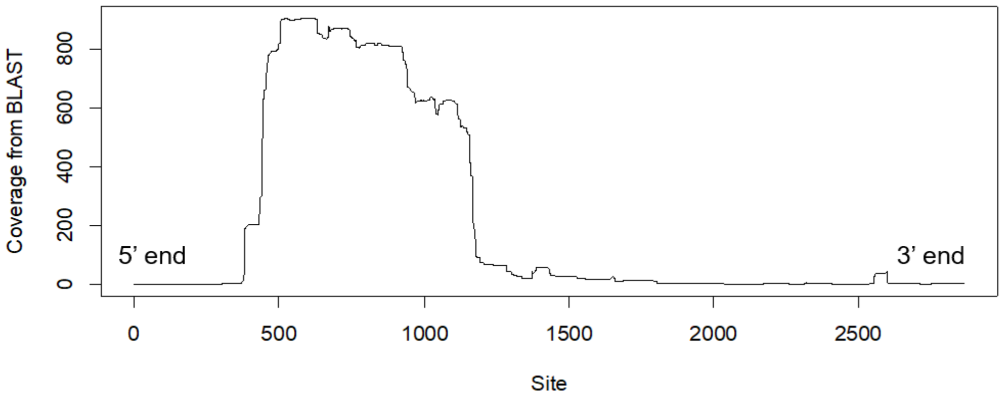

Supplementary Fig. 9. The coverage depths of *MSL* homologues along *MSL* sequence in the male *P. deltoides* genome. The x-axis shows the positions of the *MSL* sequence from 5' to 3' end. The homologues sequences show homology to *MSL* at the 5' end.

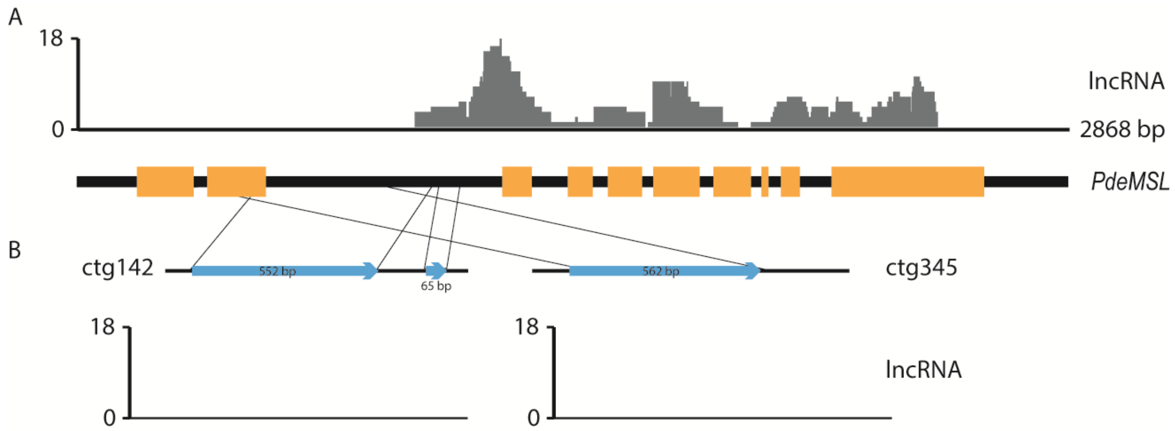

Supplementary Fig. 10. Transcription of *PdeMSL* in *P. deltoides* and transcription of the *PdeMSL* homologous segments in *P. davidiana*. a Gene structure and transcription of *PdeMSL*. The yellow boxes indicate predicted *PdeMSL* exons. The upper track visualizes the abundance of lncRNA. **b** Transcription of *PdeMSL* homologous segments in ctg345 and ctg142 in *P. davidiana*. Ctg345 resides at pericentromeric region, while ctg142 resides at peritelomeric region on chromosome 19 in genome of *P. davidiana*. The blue segments represent the *PdeMSL* homologous sequences in ctg142 and ctg345.

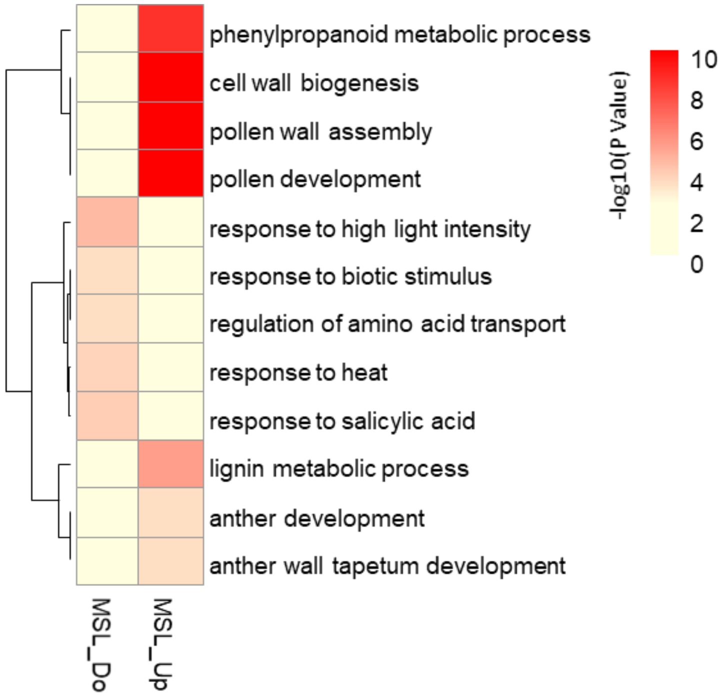

Supplementary Fig. 11. Gene ontology enrichment analysis of differentially expressed genes in transgenic Arabidopsis overexpressing *MSL*. P values were $-\log_{10}$ transformed and visualized using a heat map. Gene annotations were downloaded from TAIR (https://www.arabidopsis.org). Significantly over-represented GO terms of Biological Process category were shown in the plot.

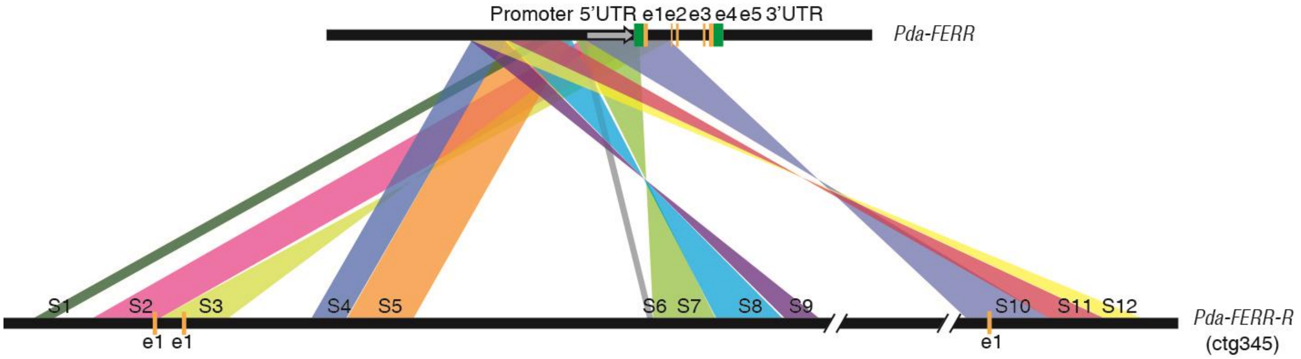

Supplementary Fig. 12. Origination of *FERR-R* in *P. davidiana*. e1-e5 represent exons of *FERR* in *P. davidiana*. S1-S12 represent the duplicated segments between *FERR-R* and *FERR* in *P. davidiana.*

Supplementary References

1. Murray, M. & Thompson, W. F. Rapid isolation of high molecular weight plant DNA. *Nucleic Acids Res.* **8**, 4321-4326 (1980).
2. Ruan, J. & Li, H. Fast and accurate long-read assembly with wtdbg2. *Nat. Methods* **17**, 155-158(2020).
3. Chin, C.-S. *et al.* Phased diploid genome assembly with single-molecule real-time sequencing. *Nat. Methods* **13**, 1050 (2016).
4. Koren, S., Walenz, B. P., Berlin, K., Miller, J. R. & Phillippy, A. M. Canu: scalable and accurate long-read assembly via adaptive *k*-mer weighting and repeat separation. *Genome Res.* **27**, 722-736 (2017).
5. Chakraborty, M., Baldwin-Brown, J.G., Long, A.D. & Emerson, J.J. Contiguous and accurate de novo assembly of metazoan genomes with modest long read coverage. *Nucleic Acids Res* **44**, e147-e147 (2016).
6. Walker, B. J. *et al.* Pilon: an integrated tool for comprehensive microbial variant detection and genome assembly improvement. *PLoS ONE* **9** (2014).
7. Servant, N. *et al.* HiC-Pro: an optimized and flexible pipeline for Hi-C data processing. *Genome Biol.* **16**, 259-259 (2015).
8. Burton, J. N. *et al.* Chromosome-scale scaffolding of *de novo* genome assemblies based on chromatin interactions. *Nat. Biotechnol.* **31**, 1119-1125 (2013).
9. Simão, F. A., Waterhouse, R. M., Ioannidis, P., Kriventseva, E. V. & Zdobnov, E. M. BUSCO: assessing genome assembly and annotation completeness with single-copy orthologs. *Bioinformatics* **31**, 3210-3212 (2015).
10. Ou, S. & Jiang, N. LTR_retriever: a highly accurate and sensitive program for identification of long terminal repeat retrotransposons. *Plant Physiol.* **176**, 1410–1422 (2018).
11. Price, A. L., Jones, N. C. & Pevzner, P. A. *De novo* identification of repeat families in large genomes. *Bioinformatics* **21**, i351-i358 (2005).
12. Edgar, R. C. & Myers, E. W. PILER: identification and classification of genomic repeats. *Bioinformatics* **21**, i152-i158 (2005).
13. Hoede, C. *et al.* PASTEC: an automatic transposable element classification tool. *PloS one* **9**, e91929 (2014).
14. Bao, W., Kojima, K. K. & Kohany, O. Repbase Update, a database of repetitive elements in

eukaryotic genomes. *Mob. DNA* **6**, 11 (2015).

15. Smit, A., Hubley, R. & Green, P. RepeatMasker Open-3.0. (1996).
16. Kim, D., Langmead, B. & Salzberg, S. L. HISAT: a fast spliced aligner with low memory requirements. *Nat. Methods* **12**, 357-360 (2015).
17. Pertea, M. *et al.* StringTie enables improved reconstruction of a transcriptome from RNASeq reads. *Nat. Biotechnol.* **33**, 290-295 (2015).
18. Haas, B. J. *et al.* De novo transcript sequence reconstruction from RNASeq using the Trinity platform for reference generation and analysis. *Nat. Protoc.* **8**, 1494 (2013).
19. Besemer, J., Lomsadze, A. & Borodovsky, M. GeneMarkS: a self-training method for prediction of gene starts in microbial genomes. Implications for finding sequence motifs in regulatory regions. *Nucleic Acids Res.* **29**, 2607-2618 (2001).
20. Haas, B. J. *et al.* Improving the *Arabidopsis* genome annotation using maximal transcript alignment assemblies. *Nucleic Acids Res.* **31**, 5654-5666 (2003).
21. Burge, C. B. & Karlin, S. Prediction of complete gene structures in human genomic DNA. *J. Mol. Biol.* **268**, 78-94 (1997).
22. Stanke, M., Schoffmann, O., Morgenstern, B. & Waack, S. Gene prediction in eukaryotes with a generalized hidden Markov model that uses hints from external sources. *BMC Bioinformatics* **7**, 62 (2006).
23. Korf, I. F. Gene finding in novel genomes. *BMC Bioinformatics* **5**, 59 (2004).
24. Keilwagen, J., Hartung, F., Paulini, M., Twardziok, S. O. & Grau, J. Combining RNASeq data and homology-based gene prediction for plants, animals and fungi. *BMC Bioinformatics* **19**, 189 (2018).
25. Allen, J. E., Pertea, M. & Salzberg, S. L. Computational gene prediction using multiple sources of evidence. *Genome Res.* **14**, 142-148 (2003).
26. Nawrocki, E. P., Kolbe, D. L. & Eddy, S. R. Infernal 1.0: inference of RNA alignments. *Bioinformatics* **25**, 1335-1337 (2009).
27. Lowe, T. M. & Eddy, S. R. tRNAscan-SE: a program for improved detection of transfer RNA genes in genomic sequence. *Nucleic Acids Res.* **25**, 955-964 (1997).
28. Birney, E., Clamp, M. & Durbin, R. GeneWise and Genomewise. *Genome Res.* **14**, 988-995 (2004).
29. She, R., Chu, S. C., Wang, K., Pei, J. & Chen, N. GenBlastA: enabling BLAST to identify

homologous gene sequences. *Genome Res.* **19**, 143-149 (2008).

30. Roach, M. J., Schmidt, S. A. & Borneman, A. R. Purge Haplotigs: allelic contig reassignment for third-gen diploid genome assemblies. *BMC Bioinformatics* **19**, 460(2018).

31. Bolger, A. M., Lohse, M. & Usadel, B. Trimmomatic: a flexible trimmer for Illumina sequence data. *Bioinformatics* **30**, 2114-2120 (2014).

32. Dobin, A. *et al.* STAR: ultrafast universal RNASeq aligner. *Bioinformatics* **29**, 15-21 (2013).

33. Anders, S. & Huber, W. Differential expression analysis for sequence count data. *Genome Boil.* **11**, 1-12 (2010).

34. Langmead, B. & Salzberg, S.L. Fast gapped-read alignment with Bowtie 2. *Nature Methods* **9**, 357-359 (2012).

35. Krueger, F. & Andrews, S. Bismark: a flexible aligner and methylation caller for Bisulfite-Seq applications. *Bioinformatics* **27**, 1571-1572 (2011).

36. Robinson JT, Thorvaldsdottir H, Wenger AM, Zehir A, Mesirov JP. Variant review with the Integrative Genomics Viewer. *Cancer Research* **77**, (2017).

37. Zerbino, D. R. & Birney, E. Velvet: Algorithms for de novo short read assembly using de Bruijn graphs. *Genome Res.* **18**(5): 821-829(2008).

38. Guy, L., Kultima, J.R. & Andersson, S.G.E. genoPlotR: comparative gene and genome visualization in R. *Bioinformatics* **26**, 2334-2335 (2010).
