# Supplementary material for: Evidences for a role of two Y-specific genes in sex determination in Populus deltoides
Source: Nat Commun. 2020 Nov 18;11:5893. doi: 10.1038/s41467-020-19559-2 (PMC7674411; doi:10.1038/s41467-020-19559-2)
Supplement: Supplementary file 4 — Description of Additional Supplementary Files [file 41467_2020_19559_MOESM4_ESM.pdf]

Description of Additional Supplementary Files

Supplementary Data 1
Gene expression data from *A. thaliana* over-expressing *FERR.*

Supplementary Data 2
MSL homology regions in *Populus* and *Salix* species

Supplementary Data 3
Gene expression data from *A. thaliana* over-expressing *MSL*.
