# Supplementary material for: Evidences for a role of two Y-specific genes in sex determination in Populus deltoides
Source: Nat Commun. 2020 Nov 18;11:5893. doi: 10.1038/s41467-020-19559-2 (PMC7674411; doi:10.1038/s41467-020-19559-2)
Supplement: Supplementary file 2 — Peer Review File [file 41467_2020_19559_MOESM2_ESM.pdf]

Reviewer #1 (Remarks to the Author):

Xue and a team including Jianquan Liu and Mark Olson here continue their work on sex chromosome evolution in Salicaceae (Zhou et al. Genome Biology 2020) which has focused on complex palindromic repeats, including the cytokinin response regulator gene ARR17 present on chromosome XIX, which is the sex chromosome in many species of the Salix/Populus clade. In the new manuscript, they report that a response regulator gene, named FERR-R, is a femaleness suppressor that generates siRNAs suppressing FERR function. The letters RR stand for response regulator; what FE stands for is not explained. In line 270, however, they report that "a recent study showed that knockout of FERR gene in female poplars [P. deltoides] converts them into males (personal communication: Dr. Niels Miller [sic] from Thünen Institute of Forest Genetics)." The person's name is Niels Müller.

I am wondering whether their FERR gene might be identical with the ARABIDOPSIS RESPONSE REGULATOR 17 gene (ARR17) that Müller focused on and knocked out, based on the discovery by Geraldes et al. (2015, in P. balsamifera) that this gene has the highest number of sex-linked single nucleotide polymorphisms and is located immediately adjacent to another sex-linked gene, namely the poplar orthologue of Arabidopsis METHYLTRANSFERASE 1 (MET1), involved in DNA methylation. I am suspecting this because Zhou et al. in their Genome Biology (2020) paper, wrote that ARR17 "is of particular interest because an ortholog of this gene has also been found to be associated with sex in Populus [24] and is therefore an excellent candidate as a sex determination gene in the Salicaceae." In short, is FERR a new discovery or a new name for ARR17?

Geraldes et al. Cronk, Recent Y chromosome divergence despite ancient origin of dioecy in poplars (Populus), Mol. Ecol., 24 (2015) 3243-3256.

Xue et al. propose that in female P. deltoides, FERR function is active due to the absence of the FERR-R gene, which is male-specific (being present only as a Y-linked copy in the YSF region [this acronym presumably means Y-specific function?] of the Y haplotype, and absent from the X-linked region). "The timing of FERR expression (only in the initiation of carpel primordia and the early development of female flower tissue) is consistent with its being a sex-determining gene." They provide no experimental support for this, but if FERR is the same as ARR17, then Müller et al. (the cited pers. comm.) with their knockout already did so.

Xue et al.'s other discovery is a male-specific expressed long non-coding RNA baptized MEl, meaning male-expressed l...? Transcripts of this locus are expressed throughout flower development, and promote maleness. Overexpression of Populus deltoides MEl in A. thaliana altered the androecium, commonly resulting in flowers with six long stamens, or seven or occasionally 8 stamens, stamens bearing two anthers, or branched stamens.

They interpret the roles of FERR and MEl as supporting the two-gene model of plant sex chromosome evolution from a pair of autosomes in an hermaphrodite ancestor, proposing that "the mechanism revealed in this study can explain the evolution of separate sexes from a monoecious ancestor by mutations in two genes with the developmental properties of FERR and MEl. The ancestor of poplar is thought to be monoecious, as the reproductive structures are catkins."

I see three problems with this interpretation. First, where is the evidence that the ancestor of poplar is monoecious? The sister genus Salix has 450 species, all dioecious, and Populus itself has 29 to 35 species, mostly dioecious. Based on parsimony, dioecy evolved early in the Salix/Populus lineage, based on molecular clocks some 35 Mya, and is still today shared by most species of the clade. Also, many sex-linked genetic markers in Populus have mapped to chromosome XIX, supporting a central role of this chromosome in sex determination throughout the genus, although in different species, the respective sex-determining loci seem to be located on different regions of chromosome XIX and seem to indicate different heterogametic sexes (Pakull 2010; Pakull et al. 2011; Paolucci et al. 2010; Gaudet 2006).

Second, why would a catkin-type inflorescence imply ancestral monoecy in Populus?

And third, when Xue et al. studied FERR and MEl in Populus davidiana, they found that FERR-R duplication occurred in a different position on chromosome 19 (in agreement with the findings I summarized above) but that Populus davidiana has no functional MEl gene, presumably due to a loss, because "inhibition of FERR by FERR-R appears sufficient for the development of androecia. Thus, MEl may no longer be essential after the FERR duplication appeared, suppressing female functions and promoting male ones."

To my mind, the sum of their own statements make clear that FERR is the single master regular gene, while MEl is one of the many other downstream genes involved in sex determination in some poplar species, but not others.

I am therefore not convinced that the present findings support the two-gene pathway towards sex chromosome evolution.

Minor comments:

Line 63: You mean ref. 4 not ref. 6

Line 112: SEMSs what does this acronym mean?

Reviewer #2 (Remarks to the Author):

17 May 2020
Review of: Xue et al. "Gene deletion and insertion control separate sexes in Poplar"

The authors examine deltoid poplar, a species with a completely sequenced genome, to determine the molecular mechanism underlying sex determination. In this the authors are following a number of similar papers that have come out recently doing similar on a number of trees and crops, such as persimmon, asparagus and grapevine. The authors propose a fairly straightforward mechanism involving the segregation of a putative femaleness gene and a putative maleness gene. The evidence for the function of these genes comes from over-expression experiments in Arabidopsis. An interesting complication comes from the fact that it is not the femaleness gene itself that segregates but a suppressor of it (which the authors suggest is actually a sort of pseudogene of the femaleness gene).

There is a lot of interesting and solid work in this paper. However much of the identification of the sex regulating genes, and their function is circumstantial, based on suggestive phenotypes in Arabidopsis (a hermaphrodite species with no close relationship with poplar). More information needs to be given here to make a better case as I explain below.

Major points
The femaleness gene (FERR) is a response regulator, which is highly plausible because many response regulators are known to have developmental effects. The authors show a very interesting regulatory link between FERR and FERR-R, the repressor. The only solid indication that this is a sex determinant, and more specifically, a female promoter, comes from transformations of FERR in arabidopsis. However, we are not told what the homologous gene is in Arabidopsis or what its mutant phenotype is. As FERR when expressed in Arabidopsis has a gynoecial phenotype it is presumably acting in the pathway of the Arabidopsis homolog of FERR, which is presumably a gynoecial developmental gene (what is the knockout phenotype in arabidopsis?). If not, then it is entirely possible that FERR is acting in pathways unrelated to what happens in poplar, and the floral phenotype is co-incidental. The

authors detail the floral phenotype in a figure but do not say what other parts of the plant are affected - we really need to know this, whether this heterologous transformation is causing general developmental disruption, including a gynoecial phenotype, or whether the phenotype is restricted to the gynoecium. One way of vastly strengthening their argument would be to report the transcriptome analysis of the Arabidopsis transformant to show that FERR is indeed affecting the correct Arabidopsis genes. This would greatly improve the case. I assume the authors have gene expression/transcriptomic data from the Arabidopsis experiments - if so why not show it?

While FERR is a nice story, albeit circumstantial (dependent on phenotypes in Arabidopsis being homologous to phenotypes in poplar), I find MEl (the maleness gene) very odd and has several problematic aspects. First of all, the central thesis of the paper that fundamental to sex determination in poplar is a pair of genes, for maleness and femaleness, working in concert. This seems to be undermined by the finding that a related poplar species has a copy of MEl, but it is not functional. Yet this species has males and females too. What then is the role (if any) of MEl in poplar? The authors hint at an answer by noting that the two species have floral differences. MEl then becomes a possible "species differentiation gene" not a "sex differentiation gene". To position the paper around a two-gene sex-determination system might therefore be misleading. Related to this is the authors claim that the lack of transcription in P. davidiana is an evolutionary loss of transcription. There is no evidence for this, it might equally be a gain of transcription in P. deltoides and MEl function might therefore be species-specific to P. deltoides. Only a survey of MEl and its transcription in a number of poplars, mapped onto a phylogeny, would answer this question. lncRNAs are well known for high transcriptional turnover: losing and gaining transcription in related species.

More fundamental is the evidence for whether MEl has any function at all in poplar. Its expression is extremely low (two orders of magnitude lower than FERR-R) and it must be near the limits of detection and near to background transcriptional noise. The authors tell us it is a lncRNA but with little evidence presented. In the suppl. table its co-ordinates are given indicating 700 bp in length, yet in the suppl. figure it is shown as 10 exons covering nearly 3000bp. What is the evidence that this is spliced, does it have a 3' poly-A tail? The authors must have these details and it would be very helpful if they were given. At these low levels of expression it is hard to see how it could be effective as a trans-acting lncRNA. The fact that it has a phenotype in Arabidopsis is a different matter as here it was overexpressed on a strong promoter and was present at presumably vastly higher level (perhaps four or five orders of magnitude higher?). It would be useful to be told what the Arabidopsis expression level was).

The only evidence that MEl is a maleness gene rather than any other gene is the Arabidopsis phenotype. This is circumstantial - we do not know whether MEl in Arabidopsis is affecting a homologous pathway as in poplar. lncRNAs are generally quite evolutionarily labile, they are formed and lost rapidly in evolutionary time. There are of course conserved lncRNAs but to conserve a highly specific lncRNA function across the c, 100 million years between poplar and Arabidopsis would be amazing if true. It would imply a highly conserved lncRNA pathway and the probable presence of a homologous lncRNA in Arabidopsis. We are not told if there is an Arabidopsis conserved homologue of this lncRNA, nor are we told whether the Arabidopsis transformants have developmental abnormalities in addition to the stamen phenotype. If so the effect of expressing the lncRNA could be affecting unknown developmental pathways different from those of poplar dioecy. It would be very interesting to repeat this transformation with (say) Nicotiana, as if the phenotype is the same then it is much less likely to be due to chance and some fundamental pathway of androecial development could have been discovered. Also, although the mode of action of MEl is unknown it would be very useful to present RNA-seq data on the Arabidopsis lines as this would add greatly to the interpretation of this experiment - if a particular relevant pathway is being affected, then the same pathway could then be looked at in poplar to test the hypothesis. There is the presentation of a hypothesis here but no testing of that hypothesis.

In summary in my view this paper would be immensely improved if we were given:

(a) more details of the structure and processing of MEl
(b) information on the homologues of FERR and MEl in Arabidopsis
(c) information on how many species MEl is expressed in: is P. deltoides the only species it is expressed in? Or is it expressed in other poplars and related plants like willows?
(d) RNA-seq data from the arabidopsis transformants. Is the FERR-R construct affecting the Arabidopsis homologue as expected? What pathway is the MEl construct affecting?
With these data it would really be possible to interpret the Arabidopsis experiments on which the manuscript hinges. Some plausible mechanism for MEl action ideally needs to be developed that could be tested by experiment.

————
Minor points
Manuscript
Abstract
l/29: “two Y genes are absent from the X” could also be mentioned that 14 X genes are absent from the Y (line 100) and therefore differ in dosage between males and females. It is not impossible that the sex determinant could be on the X and depend on a dosage effect.

l/33: “gene necessary for development of female structures”. This is a vast overclaim. The authors have nowhere demonstrated that this gene is necessary for gynoecial development. The only thing that has been demonstrated is that the gene produces an altered gynoecial phenotype in Arabidopsis. This sentence should therefore be removed.

l/85: “inherited from his sequenced”. Use of human personal pronouns very anthropomorphic for trees!

l/85: “two hemizygous fragments (which we term YHF)”: fragments usually refers to something broken or separated. Surely “sequences” would be better?

l/97: “We validated our haplotype reconstructions by amplifying and Sanger sequencing”: this worries me a bit as sex regions are often hard to assemble due to repeats. PCR-based amplification could be complicated by repeats and inverted repeats. A better way would be single molecule sequencing. The authors could say why they chose Sanger rather than a SMRT resequencing approach

l/112: “we refer to these SNPs as SEMSs” the acronym SEMS is nowhere explained. Why not call them SNPs?

l/162 and l/207: “FERR-R and MEl show male-specific expression”. This is a bit misleading. What they show is male-specific occurrence. If they occurred in females they might well be expressed in females.

l/211: “altered the androecium”: what other things were altered? Is it widely disrupting development or is it truly androecium specific?

l/247: “Many genes other than FERR-R and MEl probably function in the development of sex dimorphisms of poplars”: agreed, but how do we know that FERR-R and MEl are the critical determinants or just “other genes” with sex determination from a gene on the X via dosage effects.

l/253: “promote maleness”: fairer and more precise would be “affect the androecium in Arabidopsis” (see also comment under l. 310).

l/273: “loss of the MEl gene in P. davidiana may have occurred because”: this sentence is technically incorrect. The MEl gene is not lost in P. davidiana as the sequence is still there. My understanding from the manuscript is that it is merely not transcribed in P. davidiana. Also, it is impossible to say whether this is a loss of transcription in P. davidiana, or a gain of transcription in P. deltoides.

l/275: “MEl … male-promoting effect could be replaced by that of FERR-R, and MEl could be lost” This is a very interesting idea - however it depends on whether this is a loss of transcriptional function in P. davidiana or a gain of transcription in P. deltoides. Perhaps this could be discussed.

l/297: “deletion/insertion model”: very interesting discussion! But is there any reason why these regions have not expanded into large SDRs or whole sex chromosomes?

l/310: “MEl … producing lncRNA transcripts that promote androecium development”: this is a slight overclaim. When highly expressed in Arabidopsis it has an androecial phenotype which possesses more stamens. I recommend slight caution “appears to promote” or something like that.

Supplementary
Fig. 6 (legend): deltoids = deltoides

Reviewer #3 (Remarks to the Author):

In this report, the authors are presenting the assembly of two related poplar genome, one male and one female P. deltoides, to identify sex-specific sequences, and two sex determinants located on the Y-specific region. They proceed to the functional verification of the involvement of these two genes in sex determination. Overall, this report is well report, succinct and well organized. The figures are clear and the experiments are robust. The data presented addresses several questions marks related to sex determination in Populus species – the location of the sex determination region and the apparent versatility of this system in different species within this genus. This publication is impactful and furthers our understanding of sex determination in dioecious plants – a field that has been rapidly progressing in the last few years. The conclusions are well-supported and provide exciting new possibilities to understand the evolution of dioecy. The comparison between P, deltoides and P. davidiana is particularly interesting evolutionarily.

I have very few concerns about the data presented and would support publication of this report but I have the following major comments:

The method section is lacking entirely. I am hoping this is a mistake but, in the meantime, it is not possible to assess the validity of any of methods used, origin and pedigree of the plants analyzed or the specifics of the statistical and bioinformatic analyses. This needs to be rectified.

I am surprised by the lack of information and discussion about the potential function of FERR and MEl. Are there homologs in other species? Are there recognizable domains?

The authors performed RNA-Seq experiments and I wonder about the possibility of mapping the reads to the genomic sex-linked contigs to verify the absence of any other previously unannotated genes in those regions.

Below are more minor comments:

The authors are using two acronyms that are not intuitive: SEMS (what does this stand for exactly?), YHF (is this really necessary?). Similarly, meaning of the name of the two sex-determining genes is unclear: what do FERR and MEl stand for?

Line 62: remove “the”

Line 166: how are the different segments of FERR-R defined?

I appreciate the clarity of the model presented in Figure S5. I wonder if it would be possible to present a summary model in the main paper, summarizing the situation in P. deltoides and P. davidiana with regards to the presence of absence of FERR-R and MEl and their impact on sex.

Figure 1: How is recombination assessed if the genome of the male parent (specifically the X chromosome of the male parent) is not sequenced or known? It is difficult to assess this point without any information on the methods used. Also, would it be possible to indicate the location of the two markers within the SLR in panel B? What are the thresholds used to determine which regions are Y- or X-specific and which are not?

Figure 2: What is the significance of the two thresholds, which is used?

Figure 5: More detailed descriptions of what is shown in each panel would be useful. Which stage are the third row of pictures from? The phenotypes in B are striking. Do either overexpressing line exhibit increased or reduced seed set? What promoters are driving the expression of the transgenes?

Figure 6: Typo in deltoides

Supplemental Table 2: Please indicate which genes are FERR-R and MEl in this table

Supplementary Figure 2: What is the significance of the colors (red and blue sequence, shades of red for the alignments).

Supplementary figure 6: Can you show expression of another control lncRNA?

Isabelle Henry
UC Davis Genome Center

Reviewer #4 (Remarks to the Author):

Xue et al. identified two putative sex determination genes in poplar using comparative genomics, quantitative genetics, and transient expression experiments. They identified a small sex determination region at the telomere of chromosome 19. One of these genes represses female structures through siRNA and the other generates long non-coding RNAs that promote androecium development. I read this paper with interest, but I have a few concerns. Most importantly, neither the main text or supplement contained a methods section, making it impossible to assess the technical aspects of this manuscript including genome assembly, annotation, GWAS, and Arabidopsis transformation work. My review is therefore superficial in nature as this information is critical for evaluating the manuscript. This is especially important for the Arabidopsis work as I have no idea if genes from poplar or their Arabidopsis orthologs were overexpressed. My specific comments are outlined below making the assumption that this work is technically sound and that a methods section exists but was accidently omitted for some reason.

It is unclear how large the sex-linked region is in poplar. In line 99, 42kb of sequences were identified to be sex linked, and Figure 1 shows 299 kb, but a value is not readily provided anywhere in the text. It would also be useful to calculate the ks between paired genes in the X and Y to estimate the divergence time of these two regions. Figure 1B shows some genes between the X and Y, but a more detailed figure of synteny between these regions/haplotypes would be helpful. What about polymorphisms in the surrounding PAR?

Line 90 The difference in telomere length between the X and Y sex determination region is interesting,

but this could be due to assembly artifacts as highly repetitive regions such as the telomere often collapse during assembly. This is a relatively minor point, but this could be tested based on the sequence similarity of the telomere sequences.

Line 109. It is unclear why the female genome sequence was used for identifying SNPs co-segregating with sex. Why not use the male reference? If the SLR-Y contains sequences missing from the SLR-X, reads will not align to the female reference, skewing downstream results. Later the authors state they used the male reference to address these issues which eliminated all the non-SLR SEMSs, why not just report these results? Were any additional SEMSs identified using the male reference?

Line 161. It is possible a nonfunctional allele of a sex determination gene could have similar expression patterns to its functional counterpart, so expression alone cannot rule out these genes. Based on the downstream evidence, these three genes are likely not involved in sex, but this sentence could be reworded to reflect this (i.e. "not likely to be the sex determination genes").

Line 221. Identifying variants co-segregating with sex is not technically GWAS so this term should not be used here.

Line 256. It is unclear how loss of MEI would result in monoecious or female plants. Overexpression of MEI in Arabidopsis increased the number of stamens, suggesting this gene promotes maleness, but no knockout studies were performed to test if it is essential for male flower development. Because no methods are available, I don't know if the transformation work used the MEI gene from poplar or its Arabidopsis ortholog.

Line 270. This seems like a major finding that validates much of the work in this paper, and a personal communication is probably insufficient here. It would be useful to either reference this paper or present the actual results here. I am unsure of Nature Communications requirements, but many journals prohibit the use of personal communications of this nature.

Minor:
Some of the acronyms are not commonly used in the sex chromosome research community or are poorly defined in the text at their first use, making it a bit confusing (i.e. SEMSs, YHF, FERR)

Line 112. It is unclear what SEMS stands for.

Line 264 Both chromosome 19 and XIX are used interchangeable, but one should be used for consistency.

Line 290. It would be useful to include reference to work in papaya, which has a relatively large sex determination region located in the pericentric region.

General responses

We thank the reviewers for their detailed and stimulating comments. We have made revisions throughout the manuscript to make the experiments and reasoning clearer, as detailed below. We feel that the reviewers' suggestions have improved the manuscript, including adding new information that strengthens the evidence for our conclusions, and changing some of the abbreviations to make the meaning easier to remember (YHF is now YHS, for Y-specific hemizygous sequence), and "*MEl* (male-specifically expressed lncRNA)" is now "*MSL* (male specific lncRNA)", throughout the text. However, for clarity, we still mention the original names of these genes in the responses to the reviews below.

As there were many comments, our responses are necessarily numerous. We therefore summarize the major changes, which include adding four kinds of information, as follows:

(i) We describe evidence that the *P. deltoides FERR* gene is a member of a well-studied plant gene family, and that it is a distinct member from the *ARR16* and *ARR17* sequences (which are very similar in sequence to one another), with details shown in Supplementary Figure 7.

(ii) Evidence that, in our transgenic experiments, over-expression of the *MSL* gene in *A. thaliana* led to increased expression of a set of genes that is enriched for pollen developmental functions. Supplementary Figure 11 summarizes the results of our GO analysis.

(iii) Evidence suggesting that the different locations of the sex-determining region in Salicaceae species may correlate with the presence/absence of the *MSL* gene (Supplementary Data 2).

(iv) We also added an explanation of the point that, although *FERR-R* expression is not confined to the flower development stage when sex-determination occurs, temporal specificity is provided by *FERR*, which is expressed only during the initiation of carpel primordia and early female flower development. In such a system, it is not necessary for both interacting genes to be expressed exclusively during the sex-determination period, though both must be expressed at that stage in order to interact.

Reviewer #1:

(1) Xue and a team including Jianquan Liu and Mark Olson here continue their work on sex chromosome evolution in Salicaceae (Zhou et al. Genome Biology 2020) which has focused on complex palindromic repeats, including the cytokinin response regulator gene *ARR17* present on chromosome XIX, which is the sex chromosome in

many species of the *Salix/Populus* clade. In the new manuscript, they report that a response regulator gene, named *FERR-R*, is a femaleness suppressor that generates siRNAs suppressing *FERR* function. The letters *RR* stand for response regulator; what FE stands for is not explained. In line 270, however, they report that “a recent study showed that knockout of *FERR* gene in female poplars [*P. deltoides*] converts them into males (personal communication: Dr. Niels Müller [sic] from Thünen Institute of Forest Genetics).” The person’s name is Niels Müller.

Response: Thank you for the comments. The typo has been corrected. The sentence has been rephrased as “Finally, a recent study showed that, in *P. tremula* (in subgenus *Leuce*, like *P. davidiana*), knockout of the ortholog of the *P. deltoides FERR* gene (called *ARR17* in *P. tremula*), in female trees converted them into males[28].”, where Ref. 28 is the paper by Müller et al. 2020: A single gene underlies the dynamic evolution of poplar sex determination. *Nat. Plants* **6**, 630-637 (2020).

We also now cite the paper mentioned by the reviewer: Zhou, R. et al. A willow sex chromosome reveals convergent evolution of complex palindromic repeats. *Genome Biol.* **21**, 38(2020).

(2) I am wondering whether their *FERR* gene might be identical with the *ARABIDOPSIS RESPONSE REGULATOR 17* gene (*ARR17*) that Müller focused on and knocked out, based on the discovery by Geraldes et al. (2015, in *P. balsamifera*) that this gene has the highest number of sex-linked single nucleotide polymorphisms and is located immediately adjacent to another sex-linked gene, namely the poplar orthologue of Arabidopsis *METHYLTRANSFERASE 1* (*MET1*), involved in DNA methylation. I am suspecting this because Zhou et al. in their Genome Biology (2020) paper, wrote that *ARR17* “is of particular interest because an ortholog of this gene has also been found to be associated with sex in *Populus* [24] and is therefore an excellent candidate as a sex determination gene in the Salicaceae.” In short, is *FERR* a new discovery or a new name for *ARR17*?

Responses: The female-specifically expressed *RESPONSE REGULATOR* (*FERR*) gene (line 197 in the original manuscript) was identified as the target of siRNAs generated from a non-coding sequence in the *P. deltoides* sex determining region. *FERR* belongs to type-A *RR* gene family. Phylogenetic analysis of type-A *RR* genes (Supplementary Figure 7) shows that the *P. deltoides FERR* (*EVM0009215.1*) is not the closest homolog (the ortholog) of the *A. thaliana ARR17* or *ARR16* (the closest sequence is *EVM0036439.1*).

In contrast to the *ARR17* homolog in the previous study in *P. tremula*, which was reported to be associated with sex (sex-linked), no such association was found in *P. deltoides* when the male genome sequence was used as the reference genome (as explained in the text, use of a female genome leads to many false-positives). The *FERR* gene is located outside the *P. deltoides* sex-linked region, and is present in both sexes. Based on the differences, and the phylogeny shown above, we believe that a different gene name is justified.

The *MET1* gene was mentioned as a sex-determining candidate in previous studies in *P. trichocarpa* (Song et al. 2013; Geraldes et al. 2015). Our study clearly shows that this gene is fully sex-linked in *P. deltoides*, but is present in both X and Y haplotypes. Furthermore, its expression does not differ between the sexes. Therefore, *MET1* is not the *P. deltoides* sex determining gene.

References:

Song, Y. *et al*. Sexual dimorphic floral development in dioecious plants revealed by transcriptome, phytohormone, and DNA methylation analysis in *Populus tomentosa*. Plant Mol. Biol. **83**, 559-576(2013).

Geraldes, A. *et al*. Recent Y chromosome divergence despite ancient origin of dioecy in poplars (*Populus*). *Mol. Ecol.* **24**, 3243-3256(2015).

(3) Xue et al. propose that in female *P. deltoides*, *FERR* function is active due to the absence of the *FERR-R* gene, which is male-specific (being present only as a Y-linked copy in the YSF region [this acronym presumably means Y-specific function?] of the Y haplotype, and absent from the X-linked region). "The timing of *FERR* expression (only in the initiation of carpel primordia and the early development of female flower tissue) is consistent with its being a sex-determining gene." They provide no experimental support for this, but if *FERR* is the same as *ARR17*, then Müller et al. (the cited pers. comm.) with their knockout already did so.

Response: "YHF" stands for the Y specific hemizygous fragment (line 88 in the original manuscript). Following reviewer 2's suggestion, we have changed this to YHS (Y-specific hemizygous sequence).

FERR is specifically expressed in female flowers, and it is turned off by *FERR-R* in males. Our transformation experiments expressing *FERR* in *A. thaliana* show that it promotes pistil development. Müller et al.'s study knocked out the *P. tremula ARR17* gene, but did no overexpression experiments. Our study provides additional experimental evidence showing that the gene *FERR* is involved in sex-determination, in a different *Populus* subgenus.

(4) Xue et al.'s other discovery is a male-specific expressed long non-coding RNA baptized *MEl*, meaning male-expressed l...? Transcripts of this locus are expressed throughout flower development, and promote maleness. Overexpression of *Populus deltoides MEl* in *A. thaliana* altered the androecium, commonly resulting in flowers with six long stamens, or seven or occasionally 8 stamens, stamens bearing two anthers, or branched stamens.

They interpret the roles of *FERR* and *MEl* as supporting the two-gene model of plant sex chromosome evolution from a pair of autosomes in a hermaphrodite ancestor, proposing that "the mechanism revealed in this study can explain the evolution of separate sexes from a monoecious ancestor by mutations in two genes with the developmental properties of *FERR* and *MEl*. The ancestor of poplar is thought to be monoecious, as the reproductive structures are catkins."

I see three problems with this interpretation. First, where is the evidence that the ancestor of poplar is monoecious? The sister genus Salix has 450 species, all dioecious, and *Populus* itself has 29 to 35 species, mostly dioecious. Based on parsimony, dioecy evolved early in the *Salix/Populus* lineage, based on molecular clocks some 35 Mya, and is still today shared by most species of the clade. Also, many sex-linked genetic markers in *Populus* have mapped to chromosome XIX, supporting a central role of this chromosome in sex determination throughout the genus, although in different species, the respective sex-determining loci seem to be located on different regions of chromosome XIX and seem to indicate different heterogametic sexes (Pakull 2010; Pakull et al. 2011; Paolucci et al. 2010; Gaudet 2006). Second, why would a catkin-type inflorescence imply ancestral monoecy in *Populus*?

Responses: The name *MEl* was chosen to indicate male-specific expressed lncRNA

(line 205 in the original manuscript). This has been changed to *MSL*, for male specific lncRNA. Below, we use "*MEl/MSL*" when referring to this gene.

Poplars and willows are plants bear "catkins". Monoecy is commonly observed for these plants. However, the speculation for monoecious origin is putative. We revise the relevant discussion, see lines 283-349 in the clear copy of the revised text.

(5) And third, when Xue et al. studied *FERR* and *MEl* in *Populus davidiana*, they found that *FERR-R* duplication occurred in a different position on chromosome 19 (in agreement with the findings I summarized above) but that *Populus davidiana* has no functional *MEl* gene, presumably due to a loss, because "inhibition of *FERR* by *FERR-R* appears sufficient for the development of androecia. Thus, *MEl* may no longer be essential after the *FERR* duplication appeared, suppressing female functions and promoting male ones."
To my mind, the sum of their own statements make clear that *FERR* is the single master regular gene, while *MEl* is one of the many other downstream genes involved in sex determination in some poplar species, but not others.

Response: Thanks for the comment. Our study showed that the YHS1 (original referred as the large YHS) in *P. deltoides* contains only two genes, both of them are non-protein-coding sequences, *MEl/MSL* and *FERR-R*. *FERR-R* is the female repressor. Our transformation study showed that *MEl/MSL* promotes maleness, while having no effect on femaleness. The *MEl/MSL* gene is found in poplar genomes whose sex-determining locus is located at the peritelomeric end of chromosome XIX (for example, we also detected this gene in this location in male *P. simonii* in subgenus *Tacamahaca*, unpublished data), but not in those with their sex-determining locus in pericentromeric region that is found in subgenus *Populus*, suggesting the diverse evolution trajectory of dioecy in poplars. We performed further analysis on *MSL* and revised the relevant text intensively. In this revision, we focus on the function of this gene, and weak the discussion on its role in the evolution of diocey.

(6) I am therefore not convinced that the present findings support the two-gene pathway towards sex chromosome evolution.

Response: *MEl/MSL* was found to be involved in sex determination in *P. deltoides* based on findings: (i) the GWAS signals indicating that it is fully sex-associated; (ii) transgenic experiments indicate that its over-expression promotes male functions in multiple transgenic Arabidopsis lines. However, complete *MEl/MSL* is absent in some other poplar species. *MEl/MSL* is therefore either a new gene that evolved de novo, or it has been transposed to new locations and the duplicated sequences have been partially lost. We agree with the reviewer that whether the evolution of dioecy in poplar involves a second gene cannot be clarified based on the current data. We revised the relevant discussion, see lines 283-349 in the clear copy of the revised text.

Minor comments:
(7) Line 63: You mean ref. 4 not ref. 6

Response: Thanks. The citation has been corrected.

(8) Line 112: SEMSs what does this acronym mean?

Response: SEMSs stands for "SNPs exactly matching with sexes" (line 108 in the clear copy of the revised text).

Reviewer #2:

(1) The authors examine deltoid poplar, a species with a completely sequenced genome, to determine the molecular mechanism underlying sex determination. In this the authors are following a number of similar papers that have come out recently doing similar on a number of trees and crops, such as persimmon, asparagus and grapevine. The authors propose a fairly straightforward mechanism involving the segregation of a putative femaleness gene and a putative maleness gene. The evidence for the function of these genes comes from over-expression experiments in Arabidopsis. An interesting complication comes from the fact that it is not the femaleness gene itself that segregates but a suppressor of it (which the authors suggest is actually a sort of pseudogene of the femaleness gene).
There is a lot of interesting and solid work in this paper. However much of the identification of the sex regulating genes, and their function is circumstantial, based on suggestive phenotypes in Arabidopsis (a hermaphrodite species with no close relationship with poplar). More information needs to be given here to make a better case as I explain below.

Response: We respond to each point below.

Major points
(2) The female-specifically expressed gene (*FERR*) is a response regulator (*RR*) gene, which is highly plausible because many response regulators are known to have developmental effects. The authors show a very interesting regulatory link between *FERR* and *FERR-R*, the repressor. The only solid indication that this is a sex determinant, and more specifically, a female promoter, comes from transformations of *FERR* in *Arabidopsis*. However, we are not told what the homologous gene is in Arabidopsis or what its mutant phenotype is. As *FERR* when expressed in Arabidopsis has a gynoecial phenotype it is presumably acting in the pathway of the Arabidopsis homolog of FERR, which is presumably a gynoecial developmental gene (what is the knockout phenotype in *Arabidopsis*?). If not, then it is entirely possible that *FERR* is acting in pathways unrelated to what happens in poplar, and the floral phenotype is co-incidental. The authors detail the floral phenotype in a figure but do not say what other parts of the plant are affected - we really need to know this, whether this heterologous transformation is causing general developmental disruption, including a gynoecial phenotype, or whether the phenotype is restricted to the gynoecium. One way of vastly strengthening their argument would be to report the transcriptome analysis of the Arabidopsis transformant to show that *FERR* is indeed affecting the correct Arabidopsis genes. This would greatly improve the case. I assume the authors have gene expression/transcriptomic data from the Arabidopsis experiments - if so why not show it?

Response: In our transformation study, overexpression of *FERR* promotes pistil development, but does not affect the stamens. Very recently, Dr. Niels Müller's group from the Thünen Institute of Forest Genetics knocked out *ARR17*, the *P. tremula* homolog of *FERR*, and showed that it could be the sex determining gene. Our overexpression experiment provides additional evidence for a sex determining function of *FERR*, in a different *Populus* subgenus. We have cited Müller's paper in this revision.

FERR is a type-A *RR* gene, which has no DNA binding domain. In *A. thaliana*,

type-A *RR* genes are reported to regulate the activity of type-B *RR* genes, by a mechanism that is not very clear (Hutchison and Kieber, 2002). The *ARR3* and *ARR4*, type-A *RR* genes regulate the expression of *PHYTOCHROME B* (*phyB*) and control the circadian period of *Arabidopsis* in a cytokinin-independent manner (Salomé et al, 2006). We followed the helpful suggestion to examine the gene expression/transcriptomic data from our transgenic *A. thaliana*. We found a number of genes whose transcription is affected in our overexpression plants. However, type-A *RR* genes have a very complex regulatory network, which is not currently well characterized, and our transcriptomic data do not provide information about *FERR*'s function. We therefore list the differentially expressed genes in Supplementary Data 1. *FERR* is homologous to *A. thaliana ARR16* and *ARR17* (although not the closest homolog of these genes, see Supplementary Figure 11 in the revision). We also searched the literature for phenotypes of *arr16* or *arr17* mutants, which we list below. Loss-of-function of these genes resulted in altered plant photomorphogenesis, cell division activity or reduced root hydrotropism, but no changes in floral organs have been reported. We therefore did not add a discussion of these mutations in the section about possible *FERR* functions, but we cited these in the discussion of the *P. tremula* study that detected a sex-determining effect.

- The hypocotyl length of *arr16* seedlings were significantly shorter in dark, while it was significantly higher than that of wild type in white light (Srivastava et al., 2019).
- Single *arr16* or *arr17* mutants, which were generated by using CRISPR/Cas9, showed a moderate increase in the total leaf epidermal cell number, and this phenotype was enhanced in *arr16 arr17* double mutant. Besides, *arr16 arr17* double mutations significantly increased the stomatal number in cotyledons, while the cotyledon area was not changed (Vatén et al., 2018).
- Compared to Col-0, *arr16 arr17* double mutant displayed drastically decreased cortex cell number in the root meristem and significantly reduced root hydrotropism (Chang et al., 2019).

In our over-expression experiment, no developmental disruption was observed in the *FERR*-overexpressing *Arabidopsis* comparing to the wild type (Figure 1).

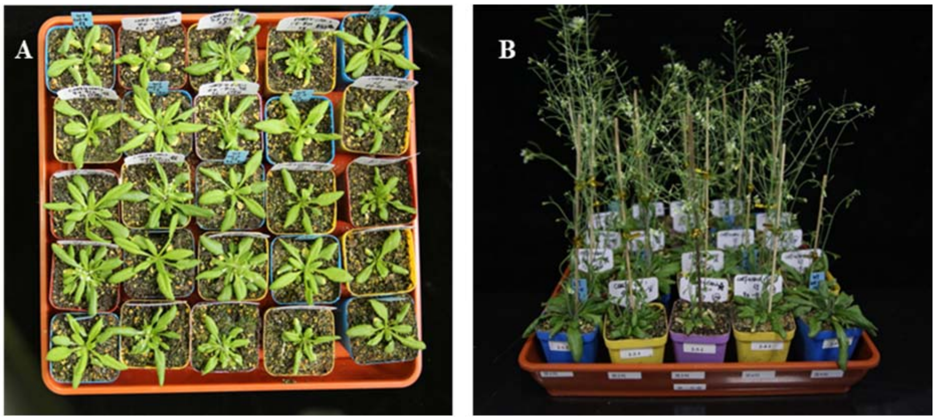

Figure 1. Overexpression of *FERR* in *Arabidopsis*. (A) Seedlings at 30 days post-germination (dpg). (B) Seedlings at 44 dpg. The pots with blue and white plastic tags indicate WT and transgenic plants, respectively.

References:

Hutchison, C, E. & Kieber, J, J. Cytokinin signaling in *Arabidopsis*. *Plant Cell* **14**, S47-S59(2002).

Chang, J. *et al*. Asymmetric distribution of cytokinins determines root hydrotropism in *Arabidopsis thaliana*. *Cell Res.* **29**, 984-993(2019).

Salomé, P. A., To, J. P., Kieber, J. J. & McClung, C. R. *Arabidopsis* response regulators ARR3 and ARR4 play cytokinin-independent roles in the control of circadian period. *Plant Cell* **18**, 55-69(2006).

Srivastava, A. K., Dutta, S., & Chattopadhyay, S. MYC2 regulates *ARR16*, a component of cytokinin signaling pathways, in *Arabidopsis* seedling development. *Plant Direct* **3**, e00177(2019).

Vatén, A., Soyars, C. L., Tarr, P. T., Nimchuk, Z. L., & Bergmann, D. C. Modulation of asymmetric division diversity through cytokinin and SPEECHLESS regulatory interactions in the *Arabidopsis* stomatal lineage. *Dev. Cell* **47**, 53-66(2018).

(3) While *FERR* is a nice story, albeit circumstantial (dependent on phenotypes in Arabidopsis being homologous to phenotypes in poplar), I find *MEl* (the maleness gene) very odd and has several problematic aspects. First of all, the central thesis of the paper that fundamental to sex determination in poplar is a pair of genes, for maleness and femaleness, working in concert. This seems to be undermined by the finding that a related poplar species has a copy of *MEl*, but it is not functional. Yet this species has males and females too. What then is the role (if any) of *MEl* in poplar? The authors hint at an answer by noting that the two species have floral differences. *MEl* then becomes a possible "species differentiation gene" not a "sex differentiation gene". To position the paper around a two-gene sex-determination system might therefore be misleading. Related to this is the authors claim that the lack of transcription in *P. davidiana* is an evolutionary loss of transcription. There is no evidence for this, it might equally be a gain of transcription in *P. deltoides* and *MEl* function might therefore be species-specific to *P. deltoides*. Only a survey of *MEl* and its transcription in a number of poplars, mapped onto a phylogeny, would answer this question. lncRNAs are well known for high transcriptional turnover: losing and gaining transcription in related species.

Response: We surveyed the in-house and publically available genome assemblies of *Populus* species, and we now provide this information in Supplementary Data 2. Like *P. deltoides* (subgenus *Aigeiros*), *P. simonii* and *P. trichocarpa* (in subgenus *Tacamahaca*), have male heterogamety (XY systems) and a sex-determining (SD) locus at the peritelomeric end of chromosome XIX. A complete copy of *MEl*/*MSL* is present in the sequenced *P. simonii* male. This gene is therefore not specific to *P. deltoides*. No complete *MEl*/*MSL* is detected in the female *P. trichocarpa* sequence, but it is unknown whether males have this gene since no male *P. trichocarpa* has yet been sequenced.

In the other sequenced poplars and willows, the SD locus is located in a different position on chromosome XIX, or on another chromosome. In these species, only homologous sequences to partial *MEl*/*MSL* were detected.

Furthermore, our study showed that *MEl/MSL* is a hemizygous gene in a Y-specific hemizygous sequence, and its expression is consistently detected. Our transformation study shows that it promotes the development of stamens, but does not affect plant growth or pistil development, suggesting that it is a maleness promoter. We agree that we should weak its role in the evolution of dioecy, and just focus on its function.

(4) More fundamental is the evidence for whether *MEl* has any function at all in

poplar. Its expression is extremely low (two orders of magnitude lower than *FERR-R*) and it must be near the limits of detection and near to background transcriptional noise. The authors tell us it is a lncRNA but with little evidence presented. In the suppl. table its co-ordinates are given indicating 700 bp in length, yet in the suppl. figure it is shown as 10 exons covering nearly 3000bp. What is the evidence that this is spliced, does it have a 3' poly-A tail? The authors must have these details and it would be very helpful if they were given. At these low levels of expression, it is hard to see how it could be effective as a trans-acting lncRNA. The fact that it has a phenotype in Arabidopsis is a different matter as here it was overexpressed on a strong promoter and was present at presumably vastly higher level (perhaps four or five orders of magnitude higher?). It would be useful to be told what the Arabidopsis expression level was).

Response: In this study, *MEl/MSL* cannot be detected using general RNA-Seq technology using oligo dT to enrich mRNA, indicating the transcripts of *MEl/MSL* do not have 3' poly-A tails. We therefore used strand-specific lncRNA-Seq to sequence the transcripts, which confirmed that the transcripts are lncRNAs (as described in our manuscript). The expression of *MEl/MSL* is detected in male flower buds (from developmental stages T1-T9, Figure 3C). In our revision, we now mention that RNAseq data from our *A. thaliana* overexpression plants estimates 5 to 20-fold higher expression than in poplar flower buds.

(5) The only evidence that *MEl* is a maleness gene rather than any other gene is the Arabidopsis phenotype. This is circumstantial - we do not know whether *MEl* in Arabidopsis is affecting a homologous pathway as in poplar. lncRNAs are generally quite evolutionarily labile, they are formed and lost rapidly in evolutionary time. There are of course conserved lncRNAs but to conserve a highly specific lncRNA function across the c, 100 million years between poplar and Arabidopsis would be amazing if true. It would imply a highly conserved lncRNA pathway and the probable presence of a homologous lncRNA in Arabidopsis. We are not told if there is an Arabidopsis conserved homologue of this lncRNA, nor are we told whether the Arabidopsis transformants have developmental abnormalities in addition to the stamen phenotype. If so the effect of expressing the lncRNA could be affecting unknown developmental pathways different from those of poplar dioecy. It would be very interesting to repeat this transformation with (say) Nicotiana, as if the phenotype is the same then it is much less likely to be due to chance and some fundamental pathway of androecial development could have been discovered. Also, although the mode of action of *MEl* is unknown it would be very useful to present RNA-seq data on the Arabidopsis lines as this would add greatly to the interpretation of this experiment - if a particular relevant pathway is being affected, then the same pathway could then be looked at in poplar to test the hypothesis. There is the presentation of a hypothesis here but no testing of that hypothesis.

Response: Thanks for this comment. We performed further analyses on this gene. Base on a *de novo* repeat library constructed from *P. deltoides* genome sequences, *MSL* is annotated as a transposal element belonging to LTR/Gypsy transposon family. A number of partial *MSL* homologous sequences are found in the *P. deltoides* genome, located either on chromosome XIX, but not in the YHS1, or on other chromosomes (Supplementary Data 2). These homologous sequences show homology with the 5' end of *MSL* sequence (Supplementary Fig. 9). The complete *MSL* is also detected in a male *P. simonii* (resides on YHS at the peritelomeric end of chromosome XIX). By

contrast, the other poplar and willow species have only partial homologous sequences (Supplementary Data 2). In genomes of *A. thaliana* and *Oryza sativa*, *MSL* sequence is completely absent. Transposable elements have been reported to generate lncRNAs in many species (references listed below). Our analysis showed that *MSL* is an LTR/Gypsy transposal element producing lncRNA transcripts. lncRNA may function in several modes, including generating siRNAs that regulate other genes, blocking the function of other siRNA/miRNA as sponges, or having direct regulatory effects on gene transcription. Our experiments do not permit a conclusion about the precise mechanism. We are currently performing transformation experiments in poplar, but these will take time.

References:

Johnson R, Guigó R. The RIDL hypothesis: transposable elements as functional domains of long noncoding RNAs. *RNA (New York, NY)* **20**, 959-976 (2014).

Wang J, *et al.* Primate-specific endogenous retrovirus-driven transcription defines naive-like stem cells. *Nature* **516**, 405-409 (2014).

Zhao T, *et al.* LncRNAs in polyploid cotton interspecific hybrids are derived from transposon neofunctionalization. *Genome Biol* **19**, 195 (2018).

Carlevaro-Fita J, Polidori T, Das M, Navarro C, Zoller TI, Johnson R. Ancient exapted transposable elements promote nuclear enrichment of human long noncoding RNAs. *Genome Res* **29**, 208-222 (2019).

(6) In summary in my view this paper would be immensely improved if we were given:
(a) more details of the structure and processing of *MEl*

Response: The relevant information has been added in the revised text (lines 230-255, as explained in the response above).

(b) information on the homologues of *FERR* and *MEl* in Arabidopsis

Response: Phylogenetic analysis shows that *FERR* is a homologous gene of *ARR16* and *ARR17* in *Arabidopsis*, while no homolog of *MEl/MSL* is detected in *Arabidopsis*.

(c) information on how many species *MEl* is expressed in: is *P. deltoides* the only species it is expressed in? Or is it expressed in other poplars and related plants like willows?

Response: Thank you for this suggestion, which we have followed and we provide this information in Supplementary Data 2. We searched in our own and publically available data, and detected a complete copy of *MEl*/*MSL* only in one other species, *P. simonii*. Partial sequences were, however, detected in other poplar species.

(d) RNA-seq data from the *Arabidopsis* transformants. Is the *FERR-R* construct affecting the Arabidopsis homologue as expected? What pathway is the *MEl* construct affecting?
With these data it would really be possible to interpret the *Arabidopsis* experiments on

which the manuscript hinges. Some plausible mechanism for *MEl* action ideally needs to be developed that could be tested by experiment.

Response: *FERR* gene (not *FERR-R*) was over expressed in our *A. thaliana* experiments. As showed in Supplementary Figure 7, *FERR* is not the closest homologous gene to *ARR16* and *ARR17* in *P. deltoides* (the closest one is *EVM0036439.1*, which is not associated with individuals' sexes). *FERR* is not in the fully sex-linked region, and is present in both sexes. The expression of this gene is blocked by *FERR-R* in male poplar, but not in females. As mentioned above, these type-A *RR* genes have no DNA binding domain, and probably function through protein interaction and modification. However, limited progress has currently been made in the characterization of the regulatory network(s) of type-A *RR* genes, and our transcriptomic data alone cannot clarify the regulatory network of *FERR*. Our revised manuscript reports a list of the genes that are differentially expressed in the overexpression lines, for both *FERR* and *MSL* (Supplementary Data 1 and 3). We also performed GO enrichment analysis using both these gene sets. Genes involved in the process of pollen development are significantly enriched in *A. thaliana* genes that were up-regulated in *A. thaliana* over-expressing the *MEl/MSL* gene. This information is now mentioned in the text. However, direct targets in *A. thaliana* are still unknown.

Finally, we also now mention that searches for *arr16* and *arr17 A. thaliana* mutants revealed no effects on flower development in the literatures.

Minor points

(7) l/29: "two Y genes are absent from the X" could also be mentioned that 14 X genes are absent from the Y (line 100) and therefore differ in dosage between males and females. It is not impossible that the sex determinant could be on the X and depend on a dosage effect.

Response: These X genes are absent from the Y, but they are individual-specific, not the consistently found in the population of the species, as a whole. No GWAS signals associated with individuals' sexes were detected in these genes.

(8) l/33: "gene necessary for development of female structures". This is a vast over claim. The authors have nowhere demonstrated that this gene is necessary for gynoecial development. The only thing that has been demonstrated is that the gene produces an altered gynoecial phenotype in Arabidopsis. This sentence should therefore be removed.

Response: We have revised the sentence to read "that block expression of a female-specifically expressed gene".

(9) l/85: "inherited from his sequenced". Use of human personal pronouns very anthropomorphic for trees!

Response: We have made the correction. Thanks.

(10) l/85: "two hemizygous fragments (which we term YHF)": fragments usually refers to something broken or separated. Surely "sequences" would be better?

Response: Thanks for the suggestion. We have changed the term to YHS (Y-specific hemizygous sequence).

(11) l/97: “We validated our haplotype reconstructions by amplifying and Sanger sequencing”: this worries me a bit as sex regions are often hard to assemble due to repeats. PCR-based amplification could be complicated by repeats and inverted repeats. A better way would be single molecule sequencing. The authors could say why they chose Sanger rather than a SMRT resequencing approach

Response: We obtained the sequence in this region based on SMRT sequencing. Our purpose was to test the haplotype reconstruction, and the Sanger sequencing yielded an exact match. The PCR amplification was also used to validate that YHS regions are present only in males, by using a larger sample size, 20 females and 20 males. This text has been clarified.

(12) l/112: “we refer to these SNPs as SEMSs” the acronym SEMS is nowhere explained. Why not call them SNPs?

Response: We use SEMSs to distinguish SNPs with genotypes matching the individuals’ sexes under male heterogamety, in other words SNPs that are homozygous in all females in our samples, but heterozygous in all the males, from other SNPs whose genotypes do not match individuals’ sexes. This text has been clarified.

(13) l/162 and l/207: “*FERR-R* and *MEl* show male-specific expression”. This is a bit misleading. What they show is male-specific occurrence. If they occurred in females, they might well be expressed in females.

Response: These sentences have been revised.

(14) l/211: “altered the androecium”: what other things were altered? Is it widely disrupting development or is it truly androecium specific?

Response: The revised text makes clear that no other phenotypes were affected, and that the effects are androecium specific.

(16) l/247: “Many genes other than *FERR-R* and *MEl* probably function in the development of sex dimorphisms of poplars”: agreed, but how do we know that *FERR-R* and *MEl* are the critical determinants or just “other genes” with sex determination from a gene on the X via dosage effects.

Response: *FERR-R* and *MEl/MSL* are the only genes associated with individuals’ sexes in our GWAS population, using coverage analysis. Some other genes with both X- and Y-linked copies were detected in the GWAS population, using SNP analysis to detect complete sex-linkage (as shown in Figure 1). However, the later sections of our manuscript describes two types of relevant evidence, (i) expression data that make these protein-coding genes less likely as sex-determination candidates than the two hemizygous non-protein-coding genes, and (ii) RNA-Seq and transgenic experiments whose results point to sex-determining functions.

(17) l/253: “promote maleness”: fairer and more precise would be “affect the androecium in Arabidopsis” (see also comment under l. 310).

Response: Thanks for the suggestion, which we have adopted.

(18) l/273: “loss of the *MEl* gene in *P. davidiana* may have occurred because”: this sentence is technically incorrect. The *MEl* gene is not lost in *P. davidiana* as the sequence is still there. My understanding from the manuscript is that it is merely not transcribed in *P. davidiana*. Also, it is impossible to say whether this is a loss of transcription in *P. davidiana*, or a gain of transcription in *P. deltoides*.

Response: We have removed this sentence.

(19) l/275: “*MEl* … male-promoting effect could be replaced by that of *FERR-R*, and *MEl* could be lost” This is a very interesting idea - however it depends on whether this is a loss of transcriptional function in *P. davidiana* or a gain of transcription in *P. deltoides*. Perhaps this could be discussed.

Response: Thanks. The proposed evolution trajectory was discussed following the reviewer’s suggestion.

(20) l/297: “deletion/insertion model”: very interesting discussion! But is there any reason why these regions have not expanded into large SDRs or whole sex chromosomes?

Response: The observation that the fully sex-linked region has not expanded into a large SDR suggests that the region has either not been fully sex-linked for a long enough evolutionary time for this to occur, or that no sexually antagonistic polymorphism became established in the PAR to select for an expanded non-recombining region.

(21) l/310: “*MEl* … producing lncRNA transcripts that promote androecium development”: this is a slight over claim. When highly expressed in Arabidopsis it has an androecial phenotype which possesses more stamens. I recommend slight caution “appears to promote” or something like that.

Response: This sentence has been revised as suggested.

(22) Fig. 6 (legend): *deltoids* = *deltoides*

Response: Thanks. The typo has been corrected.

Reviewer #3:

(1) In this report, the authors are presenting the assembly of two related poplar genome, one male and one female *P. deltoides*, to identify sex-specific sequences, and two sex determinants located on the Y-specific region. They proceed to the functional verification of the involvement of these two genes in sex determination. Overall, this report is well report, succinct and well organized. The figures are clear and the experiments are robust. The data presented addresses several questions marks related to sex determination in *Populus* species – the location of the sex determination region and the apparent versatility of this system in different species within this genus. This publication is impactful and furthers our understanding of sex determination in dioecious plants – a field that has been rapidly progressing in the last few years. The conclusions are well-supported and provide exciting new possibilities to understand the evolution of dioecy. The comparison between *P, deltoides* and *P. davidiana* is particularly interesting evolutionarily.

Response: Thanks for the positive comments.

(2) I have very few concerns about the data presented and would support publication of this report but I have the following major comments:
The method section is lacking entirely. I am hoping this is a mistake but, in the meantime, it is not possible to assess the validity of any of methods used, origin and pedigree of the plants analyzed or the specifics of the statistical and bioinformatic analyses. This needs to be rectified.

Response: We apologize for the missing method section. We have double checked to make sure all the sections are uploaded in the new submission.

(3) I am surprised by the lack of information and discussion about the potential function of *FERR* and *MEl*. Are there homologs in other species? Are there recognizable domains?

Response: Thanks for this comment. Our revised manuscript now explains that *FERR* is a type-A *RR* gene, and provides some information about this plant gene family (lines 113-118, 213-215 in the clear copy of the revised text and Supplementary Fig.7). The type-A *RR* genes have a conserved *RR* domain and are reported to negatively regulate cytokinin signaling pathway (Hellmann et al., 2010).
We performed further analyses on *MEL/MSL*. Base on a *de novo* repeat library constructed from *P. deltoides* genome sequences, *MSL* is annotated as a transposal element belonging to LTR/Gypsy transposon family. A number of partial *MSL* homologous sequences are found in the *P. deltoides* genome, located either on chromosome XIX, but not in the YHS1, or on other chromosomes (Supplementary Data 2). These homologous sequences show homology with the 5' end of *MSL* sequence (Supplementary Fig. 9). The complete *MSL* is also detected in a male *P. simonii* (resides on YHS at the peritelomeric end of chromosome XIX). By contrast, the other poplar and willow species have only partial homologous sequences (Supplementary Data 2). In genomes of *A. thaliana* and *Oryza sativa*, *MSL* sequence is completely absent. Transposable elements have been reported to generate lncRNAs in many species.

Reference:

Hellmann, E., Gruhn, N. & Heyl A. The more, the merrier: cytokinin signaling

beyond Arabidopsis. *Plant Signal. Behave*. **5**, 1384-1390 (2010).
Johnson R, Guigó R. The RIDL hypothesis: transposable elements as functional domains of long noncoding RNAs. *RNA (New York, NY)* **20**, 959-976 (2014).
Wang J*, et al.* Primate-specific endogenous retrovirus-driven transcription defines naive-like stem cells. *Nature* **516**, 405-409 (2014).
Zhao T*, et al.* LncRNAs in polyploid cotton interspecific hybrids are derived from transposon neofunctionalization. *Genome Biol* **19**, 195 (2018).
Carlevaro-Fita J, Polidori T, Das M, Navarro C, Zoller TI, Johnson R. Ancient exapted transposable elements promote nuclear enrichment of human long noncoding RNAs. *Genome Res* **29**, 208-222 (2019).

(4) The authors performed RNA-Seq experiments and I wonder about the possibility of mapping the reads to the genomic sex-linked contigs to verify the absence of any other previously unannotated genes in those regions.

Response: Thanks for the comments. We performed the analysis, but no additional genes were found to associate with sexes at population level.

Below are more minor comments:
(5) The authors are using two acronyms that are not intuitive: SEMS (what does this stand for exactly?), YHF (is this really necessary?). Similarly, meaning of the name of the two sex-determining genes is unclear: what do *FERR* and *MEl* stand for?

Response: SEMS stands for SNPs exactly matching with sexes (please see line 108 in the clear copy of the revised text). *FERR* stands for female-specifically expressed response regulator (please see line 113 in the clear copy of the revised text). We originally use *MEl/MSL* to stand for male-specific expressed lncRNA, and YHF to stand for Y-specific hemizygous fragment. Following the suggestion of reviewer 2, we changed “YHF” to “YHS (Y-specific hemizygous sequence)”, and “*MEl*” into “*MSL* (male specific lncRNA)”.

(6) Line 62: remove “the”

Response: Done.

(7) Line 166: how are the different segments of *FERR-R* defined?

Response: These segments were defined by blast searches (High Scoring Pairs, HSP). See our revised Methods section.

(8) I appreciate the clarity of the model presented in Figure S5. I wonder if it would be possible to present a summary model in the main paper, summarizing the situation in *P. deltoides* and *P. davidiana* with regards to the presence of absence of *FERR-R* and *MEl* and their impact on sex.

Response: This is a good suggestion. However, we feel that more data on *MEl/MSL* in other poplar species are needed before a summary model could be supported. This is the goal of our next paper.

(9) Figure 1: How is recombination assessed if the genome of the male parent (specifically the X chromosome of the male parent) is not sequenced or known? It is

difficult to assess this point without any information on the methods used. Also, would it be possible to indicate the location of the two markers within the SLR in panel B? What are the thresholds used to determine which regions are Y- or X-specific and which are not?

Response: The confusion is caused by the absence of our Methods section in our first submission, as this is, of course, explained in that section. We designed SSR markers based on the genome sequence of a female tree. We genotyped these SSRs in the progeny in our mapping population. In the revised manuscript, we added the locations of two SSR makers, N293 and N283, shown in panel B of Figure 1. The haplotype reconstruction was conducted following the pipeline described in the Methods (lines 65-83).

(10) Figure 2: What is the significance of the two thresholds, which is used?

Response: The black dashed line above the x-axis indicate the cut-off P value = 1e-9 (corresponding Bonferroni significance = 0.01). The red line at the top of each diagram indicate cut-off P value = 1e-137 (corresponding to Bonferroni significance = 1e-130). GWAS signals completely associated with sexes are detected using threshold above the red line. The legend has been revised to make this clearer.

(11) Figure 5: More detailed descriptions of what is shown in each panel would be useful. Which stage are the third row of pictures from? The phenotypes in B are striking. Do either overexpressing line exhibit increased or reduced seed set? What promoters are driving the expression of the transgenes?

Response: Following the reviewer's suggestion, we have added more detailed descriptions in the figure legend. Please note that it is difficult to characterize the precise stage of flowers in the third row from the top, due to the abnormal floral structure. However, by relying on the developmental stage of petals, stamens and sepals, we classified these flowers as late stages 15-16.

(12) Figure 6: Typo in *deltoides*

Response: Thanks. The typo has been corrected.

(13) Supplemental Table 2: Please indicate which genes are *FERR-R* and *MEl/MSL* in this table.

Response: Done.

(14) Supplementary Figure 2: What is the significance of the colors (red and blue sequence, shades of red for the alignments).

Response: The Figure legend has been revised to clarify the information.

(15) Supplementary figure 6: Can you show expression of another control lncRNA?

Response: Another lncRNA could potentially serve as a reference to make sure that *MEl/MSL* is reliably detected. However, the expression levels of lncRNAs vary greatly, making the choice of a reference difficult. Our experiment detected expression in male flower buds in developmental stages T1-T9 (Figure 3C), indicating reliable detection, albeit at low expression levels. Low expression does not imply that

a gene is unimportant in development. We explained above that estimating expression levels is difficult in the early development stages of flower buds. Whether *MEl/MSL* has higher expression in very early male flower buds is therefore unclear.

Reviewer #4:

(1) Xue et al. identified two putative sex determination genes in poplar using comparative genomics, quantitative genetics, and transient expression experiments. They identified a small sex determination region at the telomere of chromosome 19. One of these genes represses female structures through siRNA and the other generates long non-coding RNAs that promote androecium development. I read this paper with interest, but I have a few concerns. Most importantly, neither the main text or supplement contained a methods section, making it impossible to assess the technical aspects of this manuscript including genome assembly, annotation, GWAS, and Arabidopsis transformation work. My review is therefore superficial in nature as this information is critical for evaluating the manuscript. This is especially important for the Arabidopsis work as I have no idea if genes from poplar or their Arabidopsis orthologs were overexpressed. My specific comments are outlined below making the assumption that this work is technically sound and that a methods section exists but was accidently omitted for some reason.

Response: We apologize for the mistake of not including the Methods section. It is included in our new submission, and explains that our transgenic experiment used vectors to transform two poplar gene, *FERR* and *MEl/MSL* into *A. thaliana*.

(2) It is unclear how large the sex-linked region is in poplar. In line 99, 42kb of sequences were identified to be sex linked, and Figure 1 shows 299 kb, but a value is not readily provided anywhere in the text. It would also be useful to calculate the ks between paired genes in the X and Y to estimate the divergence time of these two regions. Figure 1B shows some genes between the X and Y, but a more detailed figure of synteny between these regions/haplotypes would be helpful. What about polymorphisms in the surrounding PAR?

Response: The size of the sex-linked region is 299 kb, as indicated in line 81 in the original version. We genotyped a full-sibling population using SSR markers. Marker N362 is located at the boundary of the fully sex-linked region. We amplified 8 fragments of the hemizygous (YHS) region in 20 males and 20 females in order to test the male specificity of the YHS regions. The PCR products from the sequenced male (42 kb) were also re-sequenced by Sanger sequencing to further validate the haplotype reconstruction.

The nucleotide divergence for synonymous sites (Ks values) for gene pairs present in both the X and Y haplotypes range from 0 to 0.148, with higher values at the telomeric end, declining towards the PAR boundary. The overall unweighted average is 0.0027, and the mean weighted by the number of synonymous sites is 2.5%, both much smaller than the mean Ks values (0.302) of “salicoid” duplication gene pairs.

Supplementary table 2 now provides information about the locations of the genes in the X and Y haplotypes shown in Figure 1B. The synteny information is also summarized in the figure below, made with the genoPlotR software. The telomeric end is at the left. The upper haplotype is the X and the lower one is the Y (in which the YHS1 hemizygous sequence is evident). We added this figure as Supplementary Figure 3.

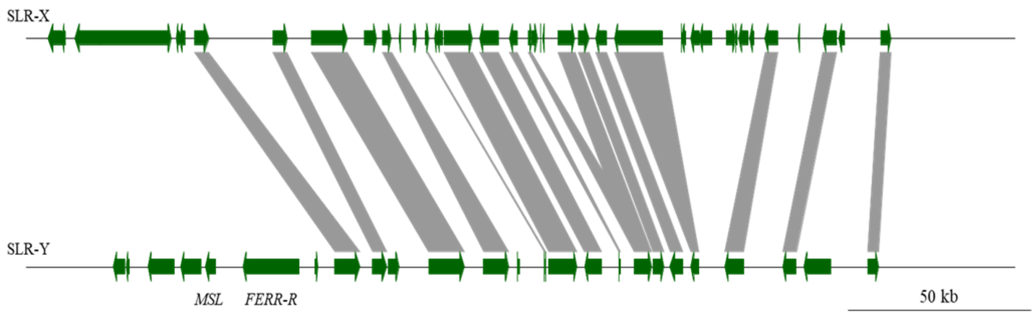

SNP density is expected to be higher in the fully sex-linked region than the PAR only if divergence between the Y and X haplotypes is large; in such cases, it can be used to determine the PAR boundary. In *P. deltoides*, polymorphism levels do not differ greatly between the region we infer to be fully sex-linked, and the PAR. Using our natural population sample to estimate SNP densities, the region between the telomeric end and the genetically mapped N362 marker has an estimated SNP density of 63.5/kb, and that in the PAR region beyond marker N362 is similar (62.6/kb). This suggests that divergence between the Y and X haplotypes is small.

(3) Line 90 The difference in telomere length between the X and Y sex determination region is interesting, but this could be due to assembly artifacts as highly repetitive regions such as the telomere often collapse during assembly. This is a relatively minor point, but this could be tested based on the sequence similarity of the telomere sequences.

Response: In our genome assembly, two contigs (of size 104 kb for the X haplotype and 141 kb for the Y one) include the two haplotypes' respective telomeres. We mapped raw PacBio raw reads onto these two contigs, which showed that both contigs are well supported by PacBio reads.

(4) Line 109. It is unclear why the female genome sequence was used for identifying SNPs co-segregating with sex. Why not use the male reference? If the SLR-Y contains sequences missing from the SLR-X, reads will not align to the female reference, skewing downstream results. Later the authors state they used the male reference to address these issues which eliminated all the non-SLR SEMSs, why not just report these results? Were any additional SEMSs identified using the male reference?

Response: In previous studies (Geraldes et al., 2015; Song et al., 2015; Sanderson et al., 2019), the sequenced female *P. trichocarpa* genome was used as references. We did this comparison to show that using the female poplar genome as reference produces false positive signals, and to show that using the male genome as the reference is important for accurate mapping of sex-linked variants.

References:

Geraldes, A. et al. Recent Y chromosome divergence despite ancient origin of dioecy in poplars (*Populus*). *Mol. Ecol.* **24**, 3243-3256 (2015).

Sanderson, B. J., Wang, L., Tiffin, P., Wu, Z. & Olson, M. S. Sex-biased gene expression in flowers, but not leaves, reveals secondary sexual dimorphism in *Populus balsamifera*. *New Phytol.* **221**, 527-539 (2019).

Song, Y., Tian, M., Ci, D. & Zhang, D. Methylation of microRNA genes regulates gene expression in bisexual flower development in andromonoecious poplar. *J. Exp. Bot*. **66**, 1891-1905 (2015).

(5) Line 161. It is possible a nonfunctional allele of a sex determination gene could have similar expression patterns to its functional counterpart, so expression alone cannot rule out these genes. Based on the downstream evidence, these three genes are likely not involved in sex, but this sentence could be reworded to reflect this (i.e. “not likely to be the sex determination genes”).

Response: The sentence has been revised as suggested.

(7) Line 221. Identifying variants co-segregating with sex is not technically GWAS so this term should not be used here.

Response: In our analysis, we used GEMMA software to analyze associations of SNPs or coverage with individuals’ sexes. Sexes were transformed to 0 (female) and 1 (male) before analysis. The Online Methods in the new submission described the analysis.

(8) Line 256. It is unclear how loss of *MEl* would result in monoecious or female plants. Overexpression of *MEl* in Arabidopsis increased the number of stamens, suggesting this gene promotes maleness, but no knockout studies were performed to test if it is essential for male flower development. Because no methods are available, I don’t know if the transformation work used the *MEl* gene from poplar or its Arabidopsis ortholog.

Responses: Before the *FERR-R* gene arose by the duplication that we discovered, loss of *MEl/MSL* would have resulted in a female genotype.

We transformed the poplar *MEl/MSL* gene into *A. thaliana*. Our Methods section explains the experiment in full.

(9) Line 270. This seems like a major finding that validates much of the work in this paper, and a personal communication is probably insufficient here. It would be useful to either reference this paper or present the actual results here. I am unsure of Nature Communications requirements, but many journals prohibit the use of personal communications of this nature.

Response: The study mentioned was published too recently to be cited in our original manuscript. We now cite this paper (Müller N A, Kersten B, Montalvão A P L, et al. A single gene underlies the dynamic evolution of poplar sex determination[J]. Nature Plants, 2020: 1-8).

Minor:
(10) Some of the acronyms are not commonly used in the sex chromosome research community or are poorly defined in the text at their first use, making it a bit confusing (i.e. SEMSs, YHF, *FERR*)

Response: We have defined all of these acronyms in the revised manuscript.

(11) Line 112. It is unclear what SEMS stands for.

Response: In the revised manuscript we define this more clearly than before.

(12) Line 264. Both chromosome 19 and XIX are used interchangeable, but one should be used for consistency.

Response: Thanks for noticing this inconsistency. The revised manuscript uses chromosome XIX throughout.

(13) Line 290. It would be useful to include reference to work in papaya, which has a relatively large sex determination region located in the pericentric region.

Response: We cite references on the studies of the papaya sex chromosomes (please see line 357 in the clear copy of the revised text).

[Editor: Reviewer #1 is unavailable. We asked Reviewer #2 to comment your responses to this reviewer.]

Reviewer #2 (Remarks to the Author):

The revised manuscript has taken into account most of the points raised by the review. The big exception concerns greater details of the genes in other systems. Relevant to this is the fact that since the reviews were returned two very interesting papers have been published that are relevant to this paper.
Zhou et al. (2020). Sequencing and Analysis of the Sex Determination Region of Populus trichocarpa. Genes, 11(8), 843.
Müller, Niels A., et al. "A single gene underlies the dynamic evolution of poplar sex determination." Nature Plants (2020): 1-8.
The Muller et al. paper is particularly interesting as it provides experimental proof that FERR (which Muller calls ARR17) is a femaleness factor. It therefore corroborates the findings here. The authors cite this paper but do not adequately discuss it. In short the Muller paper is on P. tremula (which is very closely related to P. davidiana) and postulates a single gene mechanism. Xue et al. also find a single gene mechanism in P. davidiana (FERR is active but not MSL) so is consistent. The fact that these two studies are consistent, and that P. davidiana and P. tremula are closely related (both in sect. Leuce) needs to be stated. It is of great importance than these two important studies (Muller et al. and Xue et al.) are brought together. The fact that they are consistent will advance the field.

There are three major issues to be addressed:
(1) Relevant published work not addressed fully. I have a special interest in this field and I have read most if not all the recent papers on poplar sex determination, and I don't think the relevant literature on poplar sex determination is adequately introduced or discussed here. To give the background to this paper, the Muller paper (at least) should be mentioned in the introduction. It not only provides experimental evidence for the action of FERR but the P. tremula that they investigate is very closely related to P. davidiana, which the authors investigate here. There has been a lot of work on poplar sex-determination with many authors mentioning FERR (under different names - ARR17 and RR9), including the full characterization of sex determination in P. tremula by Muller, but poplar is hardly mentioned in the introduction. The work on other species makes Xue et al.'s work more interesting, not less interesting!

(2) Names. Part of the problem is that recent papers have used different names for the FERR gene - this needs to be clearly synonymized and made clear, otherwise it is very confusing. I think the authors make an excellent contribution by renaming this gene FERR (a good name!), but it should be made clear that there has already been a lot of work on this same gene (under the names ARR17 [in Geraldes et al. 2015 and Muller et al. 2020] and PbRR9 [used in Brautigam et al., 2017 and Zhou et al., 2020]). Proposing a new name is good, but it will be really confusing if the old names for this gene in poplar research are not given.

(3) Two-gene vs single gene sex determination. This needs to be made clear. A novel part of this study on P. deltoides is that it argued to be a two gene system (FERR and MSL). However this is in direct opposition to Muller et al. who show experimentally that P. tremula (sect. Leuce) is a 1 gene system (FERR on = female, FERR off = male). Their experiment excludes any role for MSL. However, I think this is actually completely consistent with the work here as the authors show that P. davidiana is a single gene system too (no MSL activity in P. davidiana, only FERR). So I think it would be fairer to say that although you have exciting evidence of a two gene system in P. deltoides, you results also confirm a single gene system in sect. Leuce (i.e. for P. davidiana and P. tremula). This should be directly discussed: Muller has put forward a single gene model and your results need to be discussed in the light of this - I think it is very interesting and exciting that we appear to have a two-gene and a single-gene system in two different sections of poplar.

Specific points:
l.71-73 "In this study, we cloned the sex determining genes in P. deltoides, and show that previously proposed candidate genes19-23 are not involved".
>This is not the case. Many of the published studies mentioned here (19-23), and some not mentioned, like Muller et al., refer to ARR17 or RR9 (synonyms for FERR) which is definitely involved. It should be stated that FERR is also known as popARR17 (Muller) and as PbRR9 (Zhou). The presence of this gene (as ARR17) was first identified as a sex candidate by Geraldes et al., and experimentally proved to be a sex determinant by Muller et al. The gene (as PbRR9) was suggested as the sex determinant by Brautigam and recently by the diFazio group (Zhou et al.). Therefore this study represents not a discovery of FERR but an important experimental confirmation of the role of FERR/ARR17/RR9 in sex determination using the heterologous Arabidopsis system. The new discovery in this paper is the male element MSL (which is totally novel). The prior work on FERR needs to be properly acknowledged.

l.115 "We named it FERR (female-specifically expressed RESPONSE REGULATOR), based on evidence for this its function described below"
>This is a good name but it should be mentioned that it is a synonym of ARR17 (Muller) and RR9 (Zhou) - it is a new name for a much studied gene.

l.151-2 "We named the FERR duplicate FERR-R, standing for its inferred FERR repressor function"
>This is a good name but it should be noted that it is called "ARR17 inverted repeat" in Muller's study.

l.165 "Four stages of sex organ abortion are recognized". I suggest replacing "abortion" with "development". There is no abortion involved.

l.219 "Phylogenetic analysis of type-A RR genes (Supplementary Fig. 7) shows, however, that the P.deltoides FERR (EVM0009215.1) is not the orthlolog of ARR17".
>I do not understand this. Suppl fig 7 shows that EVM0009215.1 is identical to Potri.019G058900.1 which is the gene Muller calls ARR17 in his study.

l. 268 "P. davidiana belongs to the same subgenus as P. tremula and P. tremuloides, whose sex-determining regions both map to the the pericentromeric region of chromosome XIX".
>This is an important point, and it is worth discussing that the results from P. davidiana are consistent with those from P. tremula.

l. 281 "The different locations in species in the two subgenera supports the hypothesis that their dioecy evolved independently."
>This is the same conclusion made by Muller et al. for P. tremula versus P. trichocarpa. Again, it is important to mention that your work supports this conclusion with the closely related P. davidiana

l.283 "The complete MSL sequence is absent in P. davidiana."
>So therefore in P. davidiana (an aspen) sex determination is a single gene system. This supports the experimental finding by Muller et al. that sex determination in P. tremula (a European aspen) is single gene. This is very important as we have a possible two-gene system in P. deltoides, but a single gene system in aspens.

l.312 "producing a population with cosexuals and males. This state (termed androdioecy) is, however, extremely rare"
>The problem here is that dioecy in the Salicaceae appears to have evolved through the monoecy pathway. So unisexual flowers probably existed before the origin of dioecy, making single gene sex determination quite possible. This is what the Muller group argue in a recent paper.

l.350 "MSL cannot, however, be essential for male functions in all Salicaceae species, given that other poplar and willow species had only partial sequences."

>This would be a good place to discuss the finding of Muller et al in P. tremula (sect. Leuce) which experimentally shows a single gene sex determination system (which you confirm with P. davidiana).

Reviewer #3 (Remarks to the Author):

I have read this new version of the manuscript by Xue et al and found it improved substantially. In my opinion, the biggest question that remains is whether or not MSL should be considered as a sex-determinant or not. The authors have already toned down their conclusions in this regard but a more direct discussion of the fact that this is unclear would be useful. Particularly, the abstract suggests that both genes are sex determinants in P. deltoides.

I have the following questions:
Lines 315: the authors suggest that, in order for a gene to be a candidate sex determinant, it must exhibit consistently different expression in male vs female developing flowers. Given that the flowers differentiate early, isn't it possible that differential expression only early would be sufficient for sex determination? Most of the genes analyzed exhibit differential expression between male and female flowers at some, if not most developmental stages.

The recent paper by Muller et al is highly consistent with the results presented here but not entirely. A direct discussion of the differences would be welcome and could provide a broader view of sex determination in populus as a whole.

Methods. The methods are overall complete but the details are very sparse. Here are specific questions:
- How is the differential expression analysis performed in A. thaliana? Which genome / transcriptome were the reads mapped to? How many duplicates? What were the results?
- What parameters were used for all of the bioinformatic analyses performed (mapping, SNP detection, genotyping etc).
- Line 37: what constitutes a read of "valid interaction pair" (for the HiC), what criteria were used, what thresholds or parameters?
- Line 48: Did all three teams always obtain the same results regarding the sex of the trees? What happened if not?
- In lines 19 and 20 of the methods section, the authors mention that they harvested both scaled and descaled flowers at T5 as a way to assess the effect of leaving the scales for the earlier samples. The results suggest that these is large effect to descaling the flower, why is that not discussed?
- The are many small typos / mistakes in this section. Lines 21 and 22, remove "the" in front of "sex determination", lines 88 "conservatism" isn't correct, line 171 "conducted" isn't correct
- Line 93, what are MNPs?
- Line 99: how were the thresholds of 0, 1-2 and >3 derived?
- Lines 102-104: Where are the results of these realignments shown?
- Line 130: Which kits were used for the various library preps?
- Line 135: How was the rRNA/tRNA contamination removed?
- Where are the results of the DE-Seq analyses summarized? How many genes were differentially expressed (both in poplar and in A. thaliana)?
- There are three large excel files that contain data but no further explanation about what the data represents.
- The authors mention the use of BUSCO to evaluate completeness of the genomic assemblies (line 38), but where are the results of these analyses?

Reviewer #4 (Remarks to the Author):

The authors have addressed my previous comments and I appreciate their detailed responses.
The results from Muller et al. Nature Plants, 2020 and this manuscript are largely congruent, and both studies provide strong evidence that a partial duplication of ARR17/FERR-R controls sex determination in poplar and both papers suggest a similar mechanism of regulation via siRNA.
Xue et al. suggest a second sex determination gene (MEI/MSL) promotes androecium development through long non-coding RNAs. The evidence for this is somewhat weak, and I am not convinced this second gene is involved in or is essential for sex determination. The authors agree that this is not conclusive given the current data (based on their reviewer responses) but this is not clearly laid out in the manuscript. I suggest the authors revise the manuscript to better reflect the ambiguity of whether MEI/MSL is essential for sex determination in P. deltoides.

Reviewer #2:

The revised manuscript has taken into account most of the points raised by the review. The big exception concerns greater details of the genes in other systems. Relevant to this is the fact that since the reviews were returned two very interesting papers have been published that are relevant to this paper.

Zhou et al. (2020). Sequencing and Analysis of the Sex Determination Region of *Populus trichocarpa*. Genes, 11(8), 843.

Müller, Niels A., et al. "A single gene underlies the dynamic evolution of poplar sex determination." Nature Plants (2020): 1-8.

The Müller et al. paper is particularly interesting as it provides experimental proof that *FERR* (which Müller calls *ARR17*) is a femaleness factor. It therefore corroborates the findings here. The authors cite this paper but do not adequately discuss it. In short the Müller paper is on *P. tremula* (which is very closely related to *P. davidiana*) and postulates a single gene mechanism. Xue et al. also find a single gene mechanism in *P. davidiana* (*FERR* is active but not *MSL*) so is consistent. The fact that these two studies are consistent, and that *P. davidiana* and *P. tremula* are closely related (both in sect. Leuce) needs to be stated. It is of great importance than these two important studies (Müller et al. and Xue et al.) are brought together. The fact that they are consistent will advance the field.

Response: The Discussion section of our revised manuscript now highlights the fact that *P. davidiana* and P. *tremula* are from the same subgenus/section (see line 280-282, 315-316, 363-364). We also discuss these published papers in lines 67-73.

There are three major issues to be addressed:

(1) Relevant published work not addressed fully. I have a special interest in this field and I have read most if not all the recent papers on poplar sex determination, and I don't think the relevant literature on poplar sex determination is adequately introduced or discussed here. To give the background to this paper, the Müller paper (at least) should be mentioned in the introduction. It not only provides experimental evidence for the action of *FERR* but the *P. tremula* that they investigate is very closely related to *P. davidiana*, which the authors investigate here. There has been a lot of work on poplar sex-determination with many authors mentioning *FERR* (under different names-*ARR17* and *RR9*), including the full characterization of sex determination in *P. tremula* by Müller, but poplar is hardly mentioned in the introduction. The work on other species makes Xue et al.'s work more interesting, not less interesting!

Response: We have added some further relevant citations, and more fully discuss the paper by Muller et al. on poplar sex determination in the Introduction, in lines 71-73.

(2) Names. Part of the problem is that recent papers have used different names for the FERR gene - this needs to be clearly synonymized and made clear, otherwise it is very confusing. I think the authors make an excellent contribution by renaming this

gene *FERR* (a good name!), but it should be made clear that there has already been a lot of work on this same gene (under the names *ARR17* [in Geraldes et al. 2015 and Müller et al. 2020] and *PbRR9* [used in Brautigam et al., 2017 and Zhou et al., 2020]). Proposing a new name is good, but it will be really confusing if the old names for this gene in poplar research are not given.

Response: The reason we did not change this gene's name to *ARR17*, as was used for it in another poplar species, *P. tremula*, even though they are orthologs, is that our phylogenetic analysis shows that, in neither species is this gene is orthologous to the *A. thaliana ARR17* gene. For clarity, it therefore seems best to avoid the name *ARR17*, and we therefore used the name *FERR*. The revised manuscript now explains this. Following the reviewer's suggestion, we have added names of similar genes that have been found in other poplars in the Results section of this revision (lines 122-123 and 225-227).

(3) Two-gene vs single gene sex determination. This needs to be made clear. A novel part of this study on *P. deltoides* is that it argued to be a two gene system (*FERR* and *MSL*). However this is in direct opposition to Müller et al. who show experimentally that *P. tremula* (sect. *Leuce*) is a 1 gene system (*FERR* on = female, *FERR* off = male). Their experiment excludes any role for MSL. However, I think this is actually completely consistent with the work here as the authors show that *P. davidiana* is a single gene system too (no MSL activity in *P. davidiana*, only *FERR*). So I think it would be fairer to say that although you have exciting evidence of a two gene system in *P. deltoides*, you results also confirm a single gene system in sect. *Leuce* (i.e. for *P. davidiana* and *P. tremula*). This should be directly discussed: Müller has put forward a single gene model and your results need to be discussed in the light of this - I think it is very interesting and exciting that we appear to have a two-gene and a single-gene system in two different sections of poplar.

Response: Our results on *P. davidiana* in this manuscript are certainly consistent with the paper by Müller et al. on *P. tremula*, in that both these species have sex-determining loci in the centromere-proximal part of chromosome XIX, both have a duplicated *ARR17*-like gene in that region, and both species lack one of the genes (MSL) that is present within the same male-hemizygous region in two species of a different section, *P. deltoides*, and *P. simonii*. The possibility that different sections of *Populus* have different systems (one-gene and potentially two-gene systems), involving different duplications of the *FERR* gene, into different locations on chromosome XIX, makes *Populus* particularly interesting for studying the evolution of sex determination. We have revised the Discussion to make this point. See lines 315-318 and 361-368.

Specific points:

(4) l.71-73 "In this study, we cloned the sex determining genes in *P. deltoides*, and show that previously proposed candidate genes19-23 are not involved".

>This is not the case. Many of the published studies mentioned here (19-23), and

some not mentioned, like Müller et al., refer to *ARR17* or *RR9* (synonyms for *FERR*) which is definitely involved. It should be stated that FERR is also known as *popARR17* (Müller) and as *PbRR9* (Zhou). The presence of this gene (as *ARR17*) was first identified as a sex candidate by Geraldes et al., and experimentally proved to be a sex determinant by Müller et al. The gene (as *PbRR9*) was suggested as the sex determinant by Brautigam and recently by the diFazio group (Zhou et al.). Therefore, this study represents not a discovery of *FERR* but an important experimental confirmation of the role of *FERR/ARR17/RR9* in sex determination using the heterologous Arabidopsis system. The new discovery in this paper is the male element MSL (which is totally novel). The prior work on *FERR* needs to be properly acknowledged.

Response: This section has been revised to include more information (lines 67-73).

(5) l.115 "We named it *FERR* (female-specifically expressed RESPONSE REGULATOR), based on evidence for this its function described below"
>This is a good name but it should be mentioned that it is a synonym of *ARR17* (Müller) and *RR9* (Zhou) - it is a new name for a much studied gene.

Response: We revised the related contents to "A recent study demonstrated involvement of such a gene in female functions and sex-determination in *P. tremula*[24], and named the *FERR* ortholog *ARR17*[24]" (lines 122-123) and "*FERR*-like genes resembling *A. thaliana ARR17* were among candidate sex determinants in several previous studies of poplar and willow species (*PbRR9* in *P. balsamifera*[21,37], *PtRR9* or *PtRR11* in *P. trichocarpa*[38], *ARR17* in *P. tremula*[24], *RR* in *Salix purpurea*[28])" (lines 225-227)."

(6) l.151-2 "We named the *FERR* duplicate *FERR-R*, standing for its inferred *FERR* repressor function"
>This is a good name but it should be noted that it is called "*ARR17* inverted repeat" in Müller's study.

Response: When describing Müller's study, we corrected the name as suggested.

(7) l.165 "Four stages of sex organ abortion are recognized". I suggest replacing "abortion" with "development". There is no abortion involved.

Response: Done.

(8) l.219 "Phylogenetic analysis of type-A *RR* genes (Supplementary Fig. 7) shows, however, that the *P. deltoides FERR* (EVM0009215.1) is not the orthlolog of *ARR17*".
>I do not understand this. Suppl fig 7 shows that EVM0009215.1 is identical to Potri.019G058900.1 which is the gene Müller calls *ARR17* in his study.

Response: The tree does not show the *P. tremula* sequence identified by Müller et al. 2020. The *P. trichocarpa* gene Potri.019G058900.1 is in our phylogeny in Suppl fig 7, and it is shown as identical with EVM00036469.1, close to the *A. thaliana* ones called

ARR17 (and *ARR16*) as shown with green arrows in the figure. The *P. deltoides* sequence that we call *FERR* is indicated with the name and a red arrow. It is close to a different *P. trichocarpa* sequence, Potri.019G133600.3. The sentence has been revised to “Phylogenetic analysis of type-A *RR* genes (Supplementary Fig. 7) shows, however, that neither the *P. deltoides FERR* (EVM0009215.1) nor *P. trichocarpa FERR* (Potri.019G133600.3) is orthologous to the *A. thaliana ARR17* gene (the closest sequence is another gene in this family, EVM0036439.1)”.

(9) l. 268 “*P. davidiana* belongs to the same subgenus as *P. tremula* and *P. tremuloides*, whose sex-determining regions both map to the the pericentromeric region of chromosome XIX”.
>This is an important point, and it is worth discussing that the results from *P. davidiana* are consistent with those from *P. tremula*.

Response: Thanks. We revised this sentence to include discuss this, as follows: “*P. deltoides* belongs to subgenus *Aigeiros* in the genus *Populus*. To test whether the hemizygous YHS1 region is present in other poplars, we sequenced the genome of a male *P. davidiana*, in the same subgenus, *Leuce,* as *P. tremula* and *P. tremuloides* (in an earlier-branching section of *Populus* than *Aigeiros*[44]); sex-determining regions of all three subgenus *Leuce* species map to the pericentromeric region of chromosome XIX[17,29]” (see lines 279-283).

(10) l. 281 “The different locations in species in the two subgenera supports the hypothesis that their dioecy evolved independently.”
>This is the same conclusion made by Müller et al. for *P. tremula* versus *P. trichocarpa*. Again, it is important to mention that your work supports this conclusion with the closely related *P. davidiana*.

Response: We have made the suggested revisions.

(11) l.283 “The complete MSL sequence is absent in *P. davidiana*.”
>So therefore in *P. davidiana* (an aspen) sex determination is a single gene system. This supports the experimental finding by Müller et al. that sex determination in *P. tremula* (a European aspen) is single gene. This is very important as we have a possible two-gene system in *P. deltoides*, but a single gene system in aspens.

Response: Thanks. We have highlighted the similarity with *P. tremula* in this sentence in the new revision. “Neither *P. davidiana,* nor *P. tremula* has a complete *MSL* sequence.”

(12) l.312 “producing a population with cosexuals and males. This state (termed androdioecy) is, however, extremely rare”
>The problem here is that dioecy in the Salicaceae appears to have evolved through the monoecy pathway. So unisexual flowers probably existed before the origin of dioecy, making single gene sex determination quite possible. This is what the Müller

group argue in a recent paper.

Response: We agree that dioecious plants bearing catkins probably evolved from monoecious ancestors. Our argument does not, however, depend on whether the cosexual ancestor was monoecious or had prefect flowers. A mutation abolishing female flowers in a monoecious ancestor would have produced a population with monoecious cosexual individuals and males. This is an androdioecious population, not a dioecious one (similarly, a male-sterility mutation in a monoecious ancestral population creates gynrodioecy, not dioecy)

(13) l.350 "*MSL* cannot, however, be essential for male functions in all Salicaceae species, given that other poplar and willow species had only partial sequences."
>This would be a good place to discuss the finding of Müller et al in *P. tremula* (sect. *Leuce*) which experimentally shows a single gene sex determination system (which you confirm with *P. davidiana*).

Response: Thanks. We revised this sentence to read "*MSL* cannot, however, be essential for male functions in all *Salicaceae* species, given that other poplar species have only partial sequences, and that the knockout of a single gene in *P. tremula*, *ARR17,* converted female trees into males[29]". The single gene sex determination system is also discussed in the relevant context.

Reviewer #3:

(1) I have read this new version of the manuscript by Xue et al and found it improved substantially. In my opinion, the biggest question that remains is whether or not MSL should be considered as a sex-determinant or not. The authors have already toned down their conclusions in this regard but a more direct discussion of the fact that this is unclear would be useful. Particularly, the abstract suggests that both genes are sex determinants in *P. deltoides*.

Response: Thanks for the comments. *MSL* was found to be involved in sex determination in *P. deltoides* based on two findings: (i) it is found only in males (fully sex-associated), indicating complete Y-linkage; (ii) transgenic experiments indicate that its over-expression promoted male functions in multiple transgenic *A. thaliana* lines. The absent of complete *MSL* sequence in *P. davidiana* and *P. tremula* indicates that *MSL* cannot be essential for male functions in all Salicaceae species, as we explained. At the suggestion of the reviewers, we have included more information and discussion of Müller *et al.*'s 2020 paper, including the possibility that, while some poplar species have a single-gene system, species in a different section may have a two-gene system.

I have the following questions:

(2) Lines 315: the authors suggest that, in order for a gene to be a candidate sex determinant, it must exhibit consistently different expression in male vs female developing flowers. Given that the flowers differentiate early, isn't it possible that differential expression only early would be sufficient for sex determination? Most of the genes analyzed exhibit differential expression between male and female flowers at some, if not most developmental stages.

Response: Here we were emphasizing that there is no consistent sex difference. We rephrased the relevant sentence as "RT-PCR bioassays showed that none of these genes has expression limited to flower tissue, and none shows a consistent sex difference in expression".

(3) The recent paper by Müller et al is highly consistent with the results presented here but not entirely. A direct discussion of the differences would be welcome and could provide a broader view of sex determination in populus as a whole.

Response: As explained above, we have made revisions to mention this paper in several places. See lines 71-73, 290-292, 315-318 and 361-368.

Methods. The methods are overall complete but the details are very sparse. Here are specific questions:

(4) How is the differential expression analysis performed in *A. thaliana*? Which genome / transcriptome were the reads mapped to? How many duplicates? What were

the results?

Response: The updated Methods section (lines 193-200) provides the following information.
The differential expressed analysis in *A. thaliana* was performed using the same pipeline as for *Populus*. The reads were mapped to the *A. thaliana* genome sequence Araport11 (https://araport.org/). Four and eight biological replicates were collected for *FERR* and *MSL* transgenic plants, respectively. For each *FERR* or *MSL* transgenic experiment, three biological replicates of *A. thaliana* Col-0 plants were collected as wild-type controls. The results of these RNAseq data are summarized in Supplemental Data 1 and Data 3. The raw data have been deposited NCBI SRA under accession PRJNA659408.

(5) What parameters were used for all of the bioinformatic analyses performed (mapping, SNP detection, genotyping etc).

Response: We checked all the sections about bioinformatics analysis and revised some paragraphs to provide more details about the parameters, especially in the sections “GWAS analysis of sex determination” and “Quantitative analysis of Illumina reads”.

(6) Line 37: what constitutes a read of “valid interaction pair” (for the HiC), what criteria were used, what thresholds or parameters?

Response: The Method section lines 36-42 describing the HiC analysis was revised and more details were added, as follows. Interactions were identified using HiC-Pro (Servant, N. et al. 2015. Genome Biol. 16, 259-259). The *HindIII* restriction enzyme was used for library construction and read pair filtering. Uniquely mapped paired reads were screened using HiC-Pro’s default parameters, and grouped into several categories based on 1) distance between read ends, 2) mapping directions of read ends, and 3) distance of read ends to restriction enzyme sites. We excluded the following categories of interaction pairs “dangling ends”, “self-circle ligation”, “re-ligation”, “dumped pairs (outside of the expected range)”. The remaining reads were considered “valid” pairs.

(7) Line 48: Did all three teams always obtain the same results regarding the sex of the trees? What happened if not?

Response: For the few trees where results from three teams differed, further branches were collected to definitively determine the sex. This information has been added in the Method section (lines 54-55).

(8) In lines 19 and 20 of the methods section, the authors mention that they harvested both scaled and descaled flowers at T5 as a way to assess the effect of leaving the scales for the earlier samples. The results suggest that these is large effect to descaling the flower, why is that not discussed?

Response: We have added a brief discussion of the effect of de-scaling in this revised version (see lines 216-221 in main text). This was not discussed previously, to avoid lengthening the manuscript.

(9) The are many small typos / mistakes in this section. Lines 21 and 22, remove “the” in front of “sex determination”, lines 88 “conservatism” isn’t correct, line 171 “conducted” isn’t correct

Response: Thanks. The typos and mistakes have been corrected. “conservatism” was changed in to “conservation” and “conducted” in line 171 (in last version) was changed in to “collected”. We also double-checked the whole manuscript for other typos and corrected them.

(10) Line 93, what are MNPs?

Response: MNPs indicates Multiple Nucleotide Polymorphisms. In the revised method section, we removed the abbreviation of this term.

(11) Line 99: how were the thresholds of 0, 1-2 and >=3 derived?

Response: Our read-coverage based GWAS was done to check whether the presence and absence of the genomic fragments is associated with the sexes of individuals, because simply sequencing a few males and females cannot test whether this sequence difference is confined to those individuals, or a species-wide difference that reliably detects a sex-determining region. The thresholds used in the association study group the windows’ read coverage into three categories: 0 for no read, 1 for coverage 1-2, and 2 for coverage >=3 (indicating a reliable number of reads, a widely used threshold to screen reliable mapping to a reference genome sequence). In our analysis, only the primary (best) locations of the reads were included in read counting.

We revised this sentence to “To test whether the presence or absence of the genomic fragments is associated with individuals’ sexes, the windows were grouped into three read coverage categories: 0 for no read, 1 for coverage 1-2, and 2 for coverage >=3 (indicating a reliable number of reads, a widely used threshold to screen reliable mapping to a reference genome sequence). Only primary (best) locations were selected for read counting.” See lines 108-112 in the Method section.

This threshold is based on numbers of mapped reads rather than mapping quality. BWA-MEM software was used to map the reads. In this software, mapping quality indicate the possibility of reads are mapped to the reference with multiple locations. As for the length of reads, most of reads are uniquely mapped. For the reads with multiple locations, only the primary locations were counted with each window.

(12) Lines 102-104: Where are the results of these realignments shown?

Response: The re-alignment results are presented in Supplementary Figures 8A and 8B. This sentence was revised to include the information. See lines 247-250 in the main text.

(13) Line 130: Which kits were used for the various library preps?

Response: The relevant paragraph was revised to include information about the kits. "Libraries were constructed using TruSeq RNA Library Prep Kit v2 (mRNA), TruSeq Stranded Total RNA Library Prep Kit(lncRNA), TruSeq Small RNA Library Preparation Kit (small RNA) and TruSeq Methyl Capture EPIC Library Prep Kit (DNA methylation) following the manufacturers' instructions". See lines 142-145 in the Method section.

(14) Line 135: How was the rRNA/tRNA contamination removed?

Response: The sentence has been revised to "rRNA/tRNA contaminants were also removed by mapping of the reads to rRNA/tRNA sequences from public databases. The rRNA sequences of all plant species were downloaded from the SILVA rRNA database (https://www.arb-silva.de , release 104), and the tRNA sequences of *Arabidopsis thaliana* and *P. trichocarpa* were downloaded from tRNAdb (http://trna.bioinf.uni-leipzig.de/DataOutput/)." See lines 149-153 in the Method section.

(15) Where are the results of the DE-Seq analyses summarized? How many genes were differentially expressed (both in poplar and in *A. thaliana*)?

Response: The DE-seq analysis are summarized in the files Supplementary Data 1 and 3. The numbers of differentially expressed genes are shown in Supplementary Table 7 in this revision. This table is mentioned in lines 242-243 and 274-275 in the main text, and lines 197-199 of the Method section

(16) There are three large excel files that contain data but no further explanation about what the data represents.

Response: The three large Excel files are supplementary Data files. Supplementary Data 1 and 3 give the gene expression data from *A. thaliana* lines over-expressing *FERR* and *MSL*, respectively. Data 2 gives the numbers of regions with homology to *MSL* found in the published reference genome sequences of *Populus* and *Salix* species. This information is in the sentences where the Data files are cited for the first time. Supplementary Data 1(lines 242-243); Supplementary Data 2(lines 255); Supplementary Data 3(lines 274-275).

(17) The authors mention the use of BUSCO to evaluate completeness of the genomic assemblies (line 38), but where are the results of these analyses?

Response: The results are shown in Supplementary Table 2 in this revision. We revised the result section in include this information as in lines 85-87. "BUSCO analyses showed that 93.5% and 96.1% of plant conserved single-copy genes are complete in the female and male assemblies, respectively (Supplementary Table 2)".

Reviewer #4:

The authors have addressed my previous comments and I appreciate their detailed responses.

The results from Müller et al. Nature Plants, 2020 and this manuscript are largely congruent, and both studies provide strong evidence that a partial duplication of *ARR17*/*FERR-R* controls sex determination in poplar and both papers suggest a similar mechanism of regulation via siRNA.

Xue et al. suggest a second sex determination gene (*MEI/MSL*) promotes androecium development through long non-coding RNAs. The evidence for this is somewhat weak, and I am not convinced this second gene is involved in or is essential for sex determination. The authors agree that this is not conclusive given the current data (based on their reviewer responses) but this is not clearly laid out in the manuscript. I suggest the authors revise the manuscript to better reflect the ambiguity of whether *MEI/MSL* is essential for sex determination in *P. deltoides*.

Response: Thanks for the comments. *MSL* was suggested to be involved in sex determination in *P. deltoides* basing on two findings: (i) it is fully sex-linked; (ii) its over-expression promoted male functions in transgenic *A. thaliana*. Analyzing the homologous sequences in genome of *P. deltoides* shows that when it transposes to other locations, its 3' end sequence was found to have lost (Supplementary Figure 9). Complete *MSL* was detected in male *P. deltoides* and male *P. simonii* (subgenus *Tacamahaca*). The retention of a complete sequence of such an insertion for a long evolutionary time, corresponding to the divergence of two subgenera, is unexpected, and could signify that it has a plant function. In *P. davidiana* and *P. tremula*, whose sex determining locus positioned in the centromeric region, the 3' end sequence was found to have lost. The absence of complete *MSL* sequence in *P. davidiana* and *P. tremula* indicates that *MSL* cannot be essential for male functions in all Salicaceae species (as explained in the manuscript). As suggested by the reviewers, our revision includes more information and discussion of Müller et al.'s 2020 paper (see responses above). We revised the related section to include more discussion about role of *MSL* in sex determination. See lines 67-73, 319-334 and 360-367 in the main text.

Reviewer #2 (Remarks to the Author):

The authors have addressed all my comments and I think the manuscript is much improved. I have no further comments.

Reviewer #3 (Remarks to the Author):

The revised manuscript is much improved in the few critical aspects that were pointed out by the reviewers earlier. The discussion of previous literature, particularly, now seem adequate and the method section is more complete. I have no further comments and look forward to seeing this work published.
